# Polyserine-mediated targeting of FAF2/UBXD8 ameliorates tau aggregation

**DOI:** 10.1016/j.neuron.2025.08.002

**Published:** 2025-09-02

**Authors:** Meaghan Van Alstyne, Georgia Brown, Vanessa L. Nguyen, Mani Ramaswami, Charles A. Hoeffer, Roy Parker

**Affiliations:** a Department of Biochemistry, University of Colorado Boulder, CO, USA; b Howard Hughes Medical Institute, University of Colorado, Boulder, CO, USA; c Trinity College Institute of Neuroscience, School of Genetics and Microbiology, Smurfit Institute of Genetics and School of Natural Sciences, Trinity College Dublin, Dublin, Ireland; d Department of Integrative Physiology, University of Colorado Boulder, CO, USA; e BioFrontiers Institute, University of Colorado Boulder, CO, USA

**Keywords:** tau, protein aggregation, UBXD8, FAF2, polyserine, tauopathy, neurodegeneration

## Abstract

Tau aggregation is a hallmark of several neurodegenerative disorders and gain of toxic functions of misfolded tau species are linked to pathobiology. Herein, we identified proteins that limit tau aggregation when targeted to tau aggregates by polyserine domains. Polyserine targeting was most effective at mitigating tau aggregation when fused to the VCP adaptor protein, FAF2/UBXD8. Surprisingly, FAF2/UBXD8 suppresses tau aggregation independent of VCP, but does require ubiquitination, membrane localization and a UBX domain. Validation in animal models demonstrated that polyserine-targeted FAF2/UBXD8 rescues tau-induced neurodegeneration in Drosophila. Further, delivery of targeted FAF2/UBXD8 reduced gliosis, seeding capacity and insoluble tau levels in PS19 tau transgenic mice while improving contextual fear conditioning. Collectively, our findings highlight polyserine as a tau targeting strategy and identify targeted FAF2/UBXD8 as a potent suppressor of tau pathology.

**eTOC Blurb:**

Tau aggregation is a hallmark of several neurodegenerative disorders. Van Alstyne and colleagues identify polyserine targeting as a strategy to enrich fusion proteins with tau aggregates. Utilizing this approach, FAF2/UBXD8 is characterized as a suppressor of tau pathology across Drosophila and mouse animal models which is enhanced by polyserine-mediated targeting.

## Introduction

Tau inclusions are the defining hallmark of a family of neurodegenerative diseases termed tauopathies.^1^ Tau can adopt numerous disease-linked conformations that form fibrillar aggregates and possess prion-like characteristics facilitating self-propagation and spread from cell to cell via seeding.^2^ Importantly, the formation of pathogenic tau species has been causally linked to disease. Namely, mutations in tau that can promote aggregation lead to familial forms of frontotemporal dementia (FTD) and corresponding transgenic mouse models reflect aspects of disease.^2^ Additionally, delivery of patient derived seeding-competent tau in mouse models recapitulates neurotoxicity observed in tauopathies and the reduction of tau in mouse models of disease is neuroprotective.^3,4^

Protein chaperones and disaggregases can resolve misfolded toxic proteins and provide opportunity for refolding or proteolysis.^5^ For example, the yeast AAA+ ATPase Hsp104 has been re-engineered to utilize its potent disaggregase activity against several disease-linked protein aggregates including amyloid-beta (Aβ), α-synuclein, Fused in Sarcoma (FUS) and Transactive response DNA binding protein 43 (TDP-43).^5,6^ Nuclear import receptors such as transportins can also have chaperone activity mitigating aggregation of FUS and TDP-43 RNA-binding proteins.^7–10^ Underlining the importance of such cellular pathways in disease, mutations in the primary human AAA+ ATPase vasolin-containing protein (VCP) have been linked to TDP-43 accumulation in inclusion body myopathy associated with Paget disease of bone and frontotemporal dementia (IBMPFD) and amyotrophic lateral sclerosis (ALS).^11,12^ Moreover, a hypomorphic mutation in VCP is associated with tau aggregate formation in vacuolar tauopathy and VCP can disassemble tau aggregates in cellular systems.^13,14^ Thus, identifying proteins that prevent or resolve aggregation of tau could both inform on cellular mechanisms affecting tau aggregation and provide insight into therapeutic strategies.

We previously identified polyserine (polySer) domains in nuclear speckle proteins as necessary and sufficient for mislocalization to tau aggregates, which occurs in cellular models, animal models and tauopathy patient samples.^15^ In cell models, we showed that polySer containing proteins establish cytoplasmic assemblies that serve as preferred sites for tau aggregate growth and polySer levels correlate with the extent of tau aggregation in cell models.^16^ Moreover, overexpression of polySer exacerbates development of tau pathology in transgenic mice.^17^ Thus, polySer domains facilitate interaction with tau and could play a role in tau aggregation in disease contexts.

Herein, we sought to repurpose the interaction between polySer domains and tau aggregates to suppress tau aggregation. We utilized polySer as a peptide motif to target candidate proteins to tau seeds and/or aggregates to prevent further growth. We observed that transportin-3 (TNPO3), VCP, and most potently the VCP adaptor protein FAF2/UBXD8 can suppress tau aggregation in a manner augmented by polySer-based targeting. PolySer-targeted FAF2/UBXD8 (FAF2_PS_) utilizes ubiquitin recognition and membrane localization to limit tau aggregation but acts independently of VCP. Mechanistically, FAF2_PS_ is rapidly recruited to tau seeds and prevents incorporation of cellular tau into larger aggregates. Furthermore, FAF2_PS_ rescues tau-driven degeneration in Drosophila and improves tau pathology and hippocampal-dependent memory deficits in a mouse model. Thus, our findings highlight polySer as a versatile targeting motif and identify FAF2_PS_ as a potent suppressor of tau pathology.

## Results

### Polyserine is sufficient to target exogenous proteins to tau aggregates

We previously showed polySer stretches are sufficient to target a Halo reporter protein to tau aggregates in a length-dependent manner.^16^ To assess the utility of polySer-based targeting of a broader range of proteins, we generated multiple Halo-tagged fusion proteins with or without a polySer based targeting motif. These targeting motifs consisted of either a stretch of 42-polySer (PS_42_), or a C-terminal fragment of SRRM2 (PS_SRRM2_) containing two polySer stretches of 25 and 42-serines (Figure S1A).^16^

**Figure 1.**
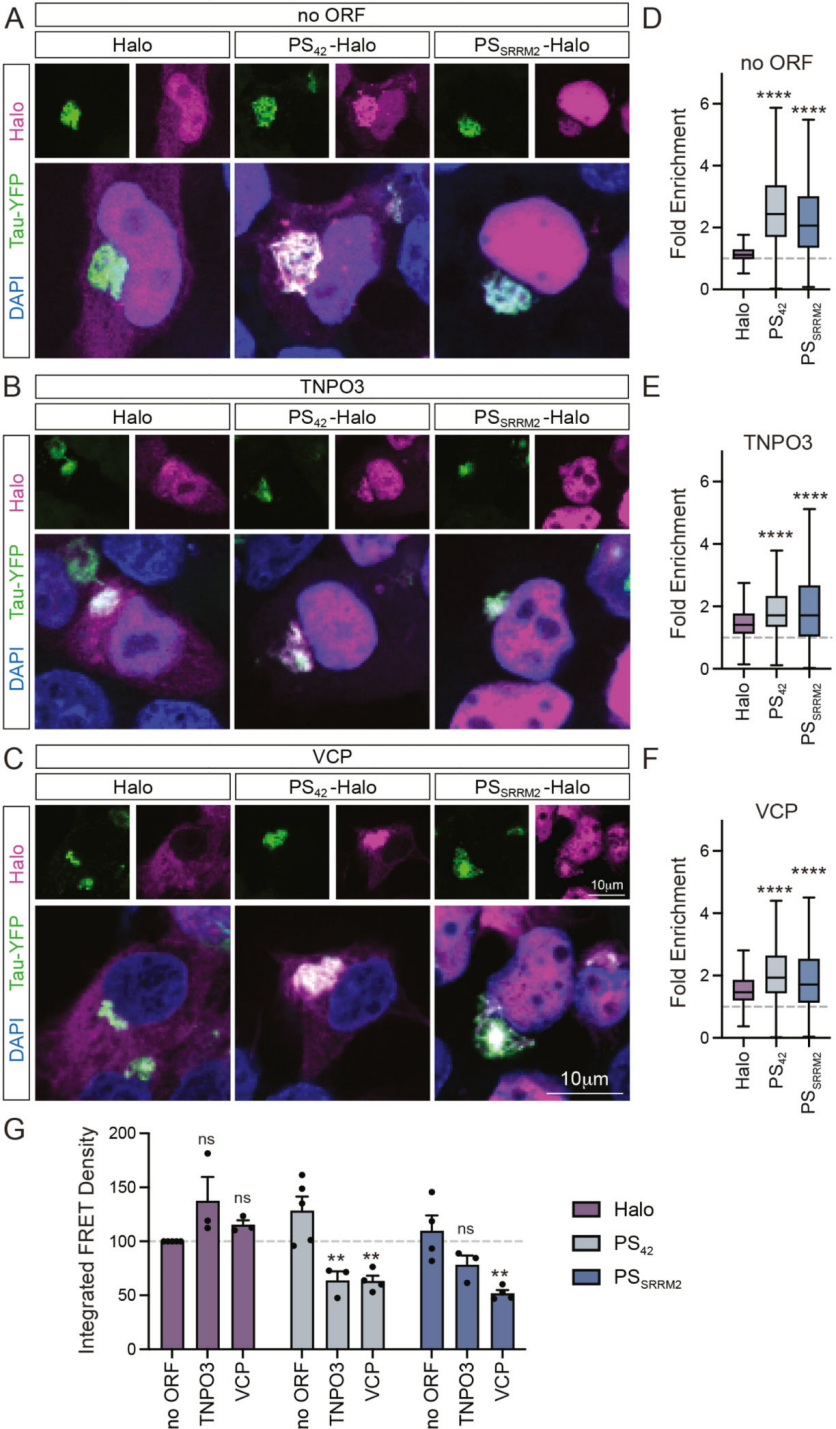
Polyserine based motifs target a range of exogenous proteins to tau aggregates. (**A-C**) Fluorescence imaging of DAPI (blue), tau-YFP (green) and Halo (magenta) in tau biosensor cells transfected with Halo, 42-polySer (PS_42_) or SRRM2-Ct (PS_SRRM2_) constructs without an upstream ORF (A), or with TNPO3 (B) or VCP (C). (**D-F**) Quantification of fold enrichment of Halo signal in cytoplasmic tau aggregates relative to cytoplasm remainder as in (A-C). Boxplot whiskers by Tukey’s method. no ORF: Halo(n=619), PS_42_(n=1222), PS_SRRM2_(n=878); TNPO3: Halo(n=370), PS_42_(n=1107), PS_SRRM2_(n=1029); VCP: Halo(n=491), PS_42_(n=468), PS_SRRM_2(n=432) cells quantified from n=3 biological replicates. One-way ANOVA and Dunnett’s multiple comparisons test (MCT). (**G**) Integrated FRET density (IFD) of top 10% of Halo+ cells via flow cytometry of biosensor cells following pre-transfection of Halo, PS_42_ or PS_SRRM2_ constructs without an upstream ORF or with TNPO3 or VCP at 24 hours post-seeding with clarified tau brain homogenate. One-way ANOVA.

Candidate fusion proteins with potential to modulate tau aggregation were selected. These proteins were HSPA8 – an Hsp70 family chaperone which has been shown to possess tau disaggregation properties in coordination with J-domain proteins *in vitro*,^18,19^ LC3B – an autophagy factor involved in autophagosome formation,^20^ the nuclear import receptors TNPO1 and TNPO3, which could limit tau aggregation analogous to activity towards FUS and TDP-43,^7,8,10^ and VCP – a segregase that can disassemble tau aggregates (Figure S1B,C).^13,14^ As LC3B is subject to post-translational cleavage and lipidation at the C-terminus the polySer motif and Halo-tag were added to the N-terminus while all other fusion proteins were C-terminally tagged (Figure S1C). All fusion proteins were detected following transfection in HEK293T tau biosensor cells that express a tau repeat domain (RD) fragment with a P301S mutation fused to CFP and YFP^21^ and did not affect tau protein levels (Figure S1D-I). Importantly, all fusion proteins showed increased enrichment in tau aggregates when targeted with polySer (Figure 1A-F, S2A-F). Therefore, a polySer motif present at either the N or C-terminus is sufficient to target a range of exogenous proteins to the local environment of tau aggregates.

### Targeting of fusion proteins augments effects on tau aggregation

To assess whether overexpression of Halo-tagged proteins in targeted or non-targeted forms modulate tau aggregation, we utilized tau biosensor cells and performed flow cytometry for CFP/YFP FRET as a measure of tau aggregation with an additional gating step to assess effects only in Halo+ cells.^16,21^ Constructs expressing Halo-tagged fusion proteins were transfected into tau biosensor cells before seeding with clarified brain homogenate from tau transgenic mice (Figure S2G). Consistent with prior results, we observed overexpression of polySer alone modestly increases tau aggregation reported as the integrated FRET density (IFD) – a product of the median FRET fluorescence intensity and percent of FRET positive cells.^16^ Expression of TNPO3 and VCP reduced aggregation when fused to polySer motifs, but had no effect without targeting (Figure 1G). HSPA8, TNPO1, and LC3B did not reduce aggregation in either circumstance (data not shown). Taken together, these findings indicate addition of polySer targeting motifs can increase the ability of exogenously expressed proteins to limit tau aggregation.

### The VCP adaptor protein FAF2/UBXD8 potently reduces tau aggregation

**Figure 2.**
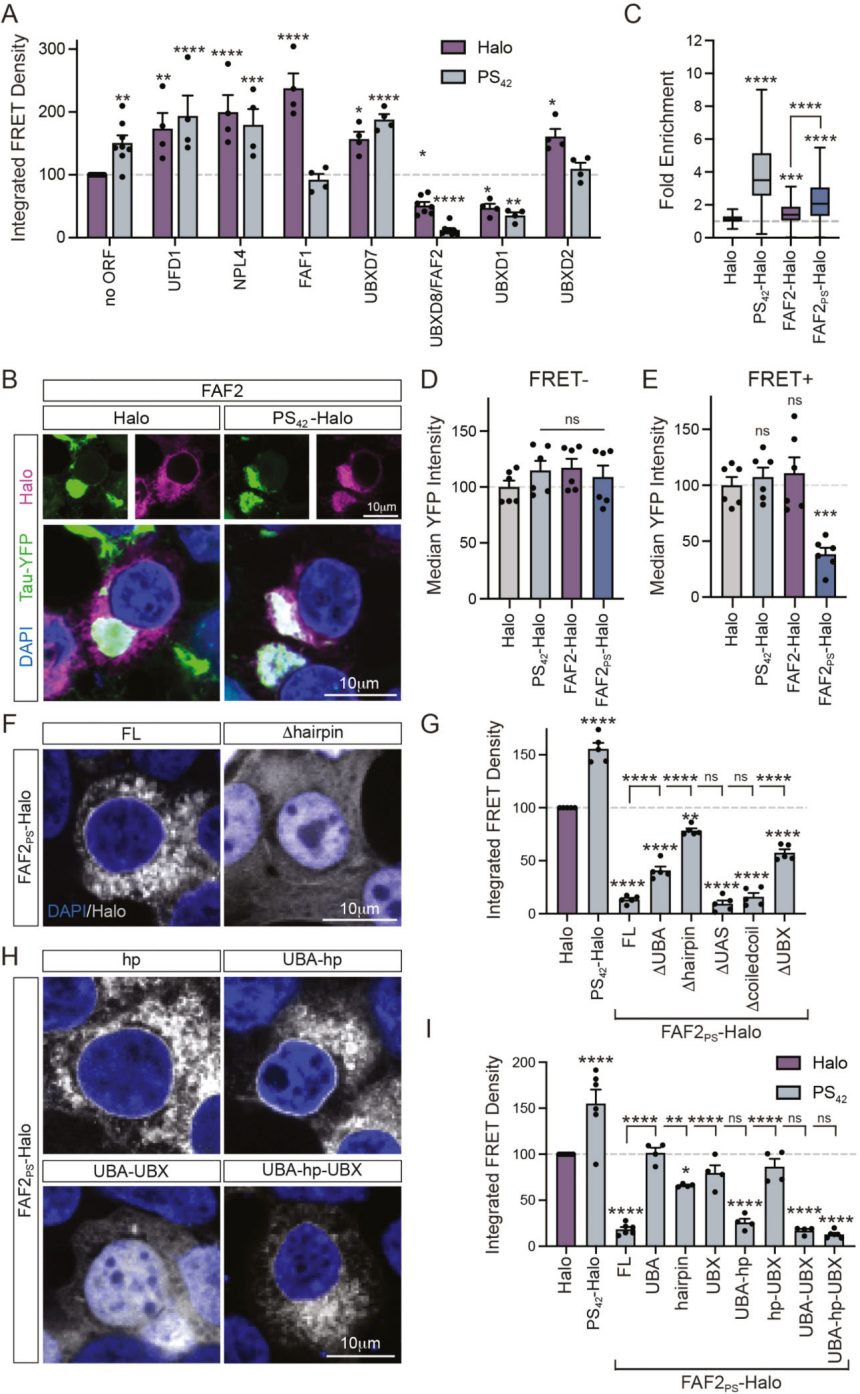
UBXD8/FAF2 amelioration of tau aggregation is enhanced by polyserine targeting. (**A**) IFD of top 10% of Halo+ cells via flow cytometry of biosensor cells following pre-transfection of Halo or PS_42_ tagged constructs without an upstream ORF or with VCP adaptors at 24 hours post-seeding with clarified tau brain homogenate and normalized to Halo control. no ORF(n=8), FAF2/UBXD8(n=7), all others(n=4). Statistics relative to no ORF control for Halo or PS_42_ groups with one-way ANOVA and Dunnett’s MCT. (**B**) Fluorescence imaging of DAPI (blue), tau-YFP (green) and Halo (magenta) in tau biosensor cells co-transfected with clarified tau brain homogenate and FAF2-Halo or FAF2_PS_-Halo. (**C**) Quantification of fold enrichment of Halo signal in cytoplasmic tau aggregates in tau biosensor cells transfected with Halo, PS_42_-Halo, FAF2-Halo and FAF2_PS_-Halo and clarified tau brain homogenate. Boxplot whiskers by Tukey’s method. Halo(n=501), PS_42_(n=366), FAF2-Halo(n=543), FAF2_PS_-Halo(n=553) cells quantified from n=3 biological replicates. One-way ANOVA and Tukey’s MCT. (**D**) Median YFP intensity in FRET-cells normalized to Halo control. One-way ANOVA and Dunnett’s MCT. n=6. (**E**) Median YFP intensity in FRET+ cells normalized to Halo control. One-way ANOVA and Dunnett’s MCT. n=6. (**F**) Fluorescence imaging of DAPI (blue) and Halo (grey) in tau biosensor cells transfected with PS_42_-fused full-length (FL) FAF2 or hairpin deletion mutant. (**G**) IFD of top 10% of tau biosensor cells pre-transfected with Halo, PS_42_-Halo and FAF2_PS_-Halo FL or deletion mutants seeded with tau brain homogenate and normalized to Halo. One-way ANOVA and Tukey’s MCT. n=5. (**H**) Fluorescence imaging of DAPI (blue) and Halo (grey) in tau biosensor cells transfected with FAF2 fragments fused with PS_42_ and Halo as in Figure S5G (see also Figure S5I). (**I**) IFD of top 10% of Halo+ tau biosensor cells pre-transfected with Halo, PS_42_-Halo and FAF2 fragments as detailed in Figure S5G seeded with clarified tau brain homogenate and normalized to Halo control. One-way ANOVA and Tukey’s MCT. Halo, PS_42_-Halo, FL, UBA-hpUBX n=6; UBA, hairpin, UBX, UBA-hp, hp-UBX, UBA-UBX n=4.

VCP is a segregase that interacts with ubiquitinated cargo through a number of adaptors and utilizes ATP hydrolysis to extract proteins from complexes.^22^ As VCP is an abundant protein, we hypothesized that targeting a VCP adaptor to tau aggregates with polySer might limit tau aggregation to a greater extent than VCP itself. Given this, we generated Halo and PS_42_-Halo tagged forms of VCP adaptor proteins with both ubiquitin and VCP binding domains (UFD1, NPL4, FAF1, UBXD7, FAF2/UBXD8) or only VCP binding domains (UBXD1 and UBXD2) (Figure S3A). We performed flow cytometry following transfection of targeted and non-targeted constructs, seeding with brain homogenate and evaluated effects only in transfected cells by gating for the top 10% of highly expressing Halo+ cells (Figure 2A).

This experiment identified two classes of VCP adaptors that affected tau aggregation. First, we observed several adaptors including UFD1, NPL4, FAF1, UBXD7 and UBXD2 increased tau aggregation when overexpressed, often independent of whether they were fused to polySer domains (Figure 2A). The second class of adaptors consisting of FAF2 and UBXD1 suppressed tau aggregation (Figure 2A).

Targeting with polySer enhanced the effects of FAF2 as overexpression led to a 50% reduction in tau aggregation while polySer targeted FAF2 (FAF2_PS_) led to a ~85% reduction (Figure 2A). Consistent with other fusion proteins, the addition of a polySer motif to FAF2 increased the fold enrichment in tau aggregates quantified by fluorescence imaging (Figure 2B,C). Addition of polySer to UBXD1 did not have as pronounced of an effect but also trended towards increased suppression of tau aggregation as compared to UBXD1 alone (Figure 2A).

To evaluate whether polySer increased the efficacy of FAF2 through increased targeting to tau as opposed to providing a longer flexible linker between the C-terminus of FAF2 and a Halo tag, we generated a construct with a 42-residue repeat of the protein sequence “SGGGG” in place of polySer (Figure S3B). Importantly, this fusion protein had no significant enrichment in tau aggregates relative to FAF2-Halo and did not show improved suppression of tau aggregation (Figure S3C-E). Thus, these results are consistent with polySer targeting conferring the improved inhibition of tau aggregate formation.

One method through which FAF2_PS_ could affect tau aggregation is by altering tau levels. However, we observe that FAF2 or FAF2_PS_ does not reduce total tau monomer levels measured by Western blot when expressed in unseeded biosensor cells that lack tau aggregates (Figure S3F,G).

To examine if FAF2_PS_ altered tau levels in cells with aggregates, we seeded tau biosensor cells expressing FAF2_PS_, and assessed tau protein levels by YFP signal through flow cytometry. Importantly, we observed FAF2_PS_ expressing cells with aggregates (FRET+) showed lower tau levels than cells without aggregates (FRET-) (Figure S3H,I). The effect of FAF2_PS_ on tau levels is also illustrated by the median YFP signal, which is reduced by FAF2_PS_ specifically in cells with tau aggregates (FRET+) and unchanged in cells without tau aggregates (FRET-) (Figure 2D,E). This reduction in tau protein levels suggests FAF2_PS_ specifically promotes degradation of aggregate-competent tau.

Collectively, this data identifies FAF2 as a VCP adaptor with the unique capability of not only preventing tau aggregation but also reducing tau levels in cells with aggregated tau species with polySer-mediated targeting enhancing effects.

### FAF2 suppresses tau aggregate formation without increasing seeding capacity

Since VCP can disassemble larger tau aggregates at the expense of creating more seeding competent species,^14^ FAF2 could act through analogous mechanisms. To address this possibility, we performed re-infection experiments by transfecting lysates from cells expressing Halo-tagged constructs and seeded to form tau aggregates back into tau biosensor cells and measured seeding capacity by flow cytometry (Figure S4A). We observed that lysates from FAF2_PS_ expressing cells had reduced seeding capacity relative to controls (Figure S4B).

However, as there are fewer aggregates in FAF2_PS_ expressing cells under these conditions, we seeded biosensor cells to pre-form aggregates over 24 hours prior to transfection of FAF2_PS_ or control constructs (Figure S4C to better evaluate the potential for FAF2 to generate increased seeding competent species. polySer targeting was similarly effective on pre-formed aggregates as when seeding and plasmids transfection are performed concomitantly (Figure S4D,E). Under these conditions we observe a similar extent of aggregation across groups at 48 and 72 hours post-seeding (Figure S4F). In re-infection experiments from lysates generated in this manner, we observe no significant differences in the seeding capacity of lysates with or without FAF2 constructs (Figure S4G). Thus, FAF2 does not reverse existing tau aggregates or increase seeding competent tau species suggesting it acts distinctly from VCP.

### FAF2/UBXD8 mitigates tau aggregation through ubiquitin recognition, membrane localization and the UBX domain

To understand how FAF2 might limit tau aggregation we considered FAF2 properties and function. FAF2 is a cytosolic facing monotopic membrane protein that can localize to the ER and lipid droplets (Figure S5A).^23,24^ At the ER specifically, FAF2 forms complexes with additional factors involved in ER-associated degradation of terminally misfolded membrane and secretory proteins through VCP and proteasomal degradation.^25^

To determine what features of FAF2_PS_ are involved in suppressing tau aggregation, we generated Halo-tagged deletion mutants (Figure S5B). Expression of constructs was validated by Western blot and localization monitored by fluorescence imaging (Figure 2F, S5C,D). Removal of the membrane insertion hairpin domain resulted in a shift from a membrane-like to diffuse cytoplasmic localization (Figure 2F) whereas all other deletions localized similarly to full-length protein (Figure S5D).

We evaluated activity of each deletion mutant on tau aggregation. Deletion of the ubiquitin-associated (UBA), hairpin, and ubiquitin regulator X (UBX) domains significantly reduced FAF2_PS_ inhibition of tau aggregation (Figure 2G). Functionally, UBA domains facilitate ubiquitin binding and UBX domains adopt ubiquitin-like folds that serve as VCP binding sites.^26,27^ In contrast, the UAS and coiled-coil domains were dispensable for this function (Figure 2G).

To assess which domains were minimally required, we designed constructs expressing FAF2 fragments of each required domain individually, pairwise, or together (Figure S5G). Western blotting validated expression and fluorescence imaging demonstrated the hairpin domain alone is required and sufficient for membrane-like localization (Figure 2H, S5H,I). Evaluation of the activity of FAF2 fragments on tau aggregation supported several conclusions. First, the three functional domains (UBA-hp-UBX) suppress tau aggregation as effectively as full-length (FL) FAF2 (Figure 2I). Second, while UBA and UBX domains do not have effects on their own, when together (UBA-UBX) they strongly suppress tau aggregation despite the subcellular localization differing due to loss of the hairpin domain (Figure 2I,H). Lastly, the hairpin domain (hp) has a small but significant suppressive effect on tau aggregation independently, which is stronger when combined with the UBA domain (UBA-hp).

Collectively, these results demonstrate FAF2 UBA, hairpin, and UBX domains are sufficient to suppress tau aggregation when targeted with polySer. Further, they suggest at least two mechanisms through which FAF2_PS_ may suppress tau aggregation. The first relies on the hairpin domain which can function independently and the second requires cooperation of the UBA and UBX domains.

### FAF2/UBXD8 suppression of tau aggregation is independent of VCP

**Figure 3.**
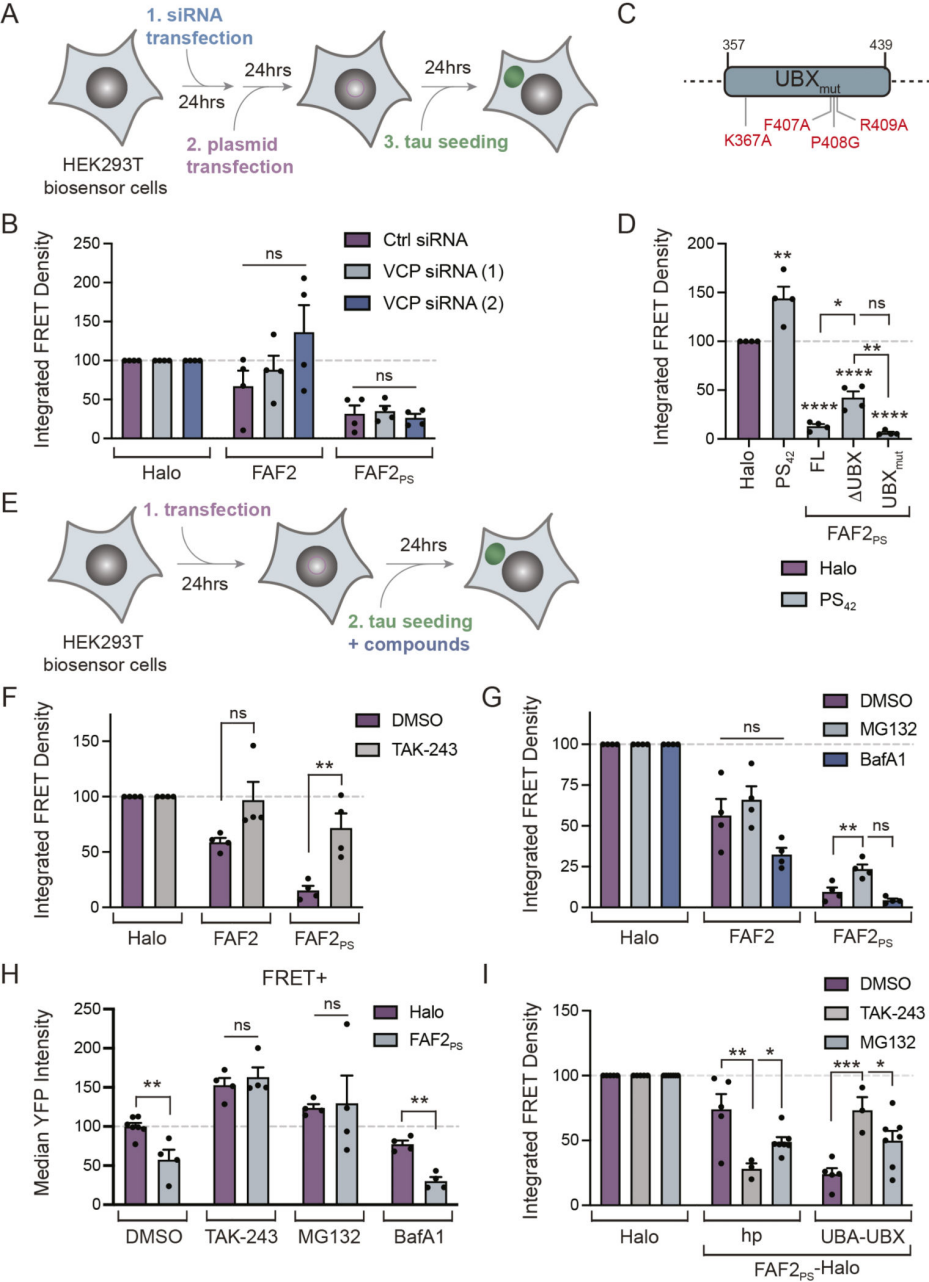
FAF2/UBXD8 suppression of tau aggregation requires ubiquitination and proteasome activity. (**A**) Timeline for experiments with siRNA in (B). (**B**) IFD of top 10% of Halo+ tau biosensor cells transfected with control or two VCP siRNAs, Halo-tagged FAF2 or FAF2_PS_ and clarified tau brain homogenate. Statistics across FAF2 or FAF2_PS_ groups with one-way ANOVA and Tukey’s MCT. n=4. (**C**) FAF2 UBX domain mutations to abolish VCP binding. (**D**) IFD of the top 10% of Halo+ tau biosensor cells transfected with Halo, PS_42_, and polySer-targeted FAF2 either full-length (FL), UBX deletion (ΔUBX) or mutated UBX domain (UBX_mut_) as described in (C) and seeded with tau brain homogenate. One-way ANOVA with Tukey’s MCT. n=4. (**E**) Timeline for experiments with compound addition in (F-I). (**F**) IFD of top 10% of Halo+ tau biosensor cells pre-transfected with Halo-tagged FAF2 or FAF2_PS_, seeded with tau brain homogenate, treated with DMSO or TAK-243 [0.2 μM], and normalized to Halo control. Statistics within FAF2 or FAF2_PS_ treatment groups with unpaired t-test. n=4. (**G**) IFD of the top 10% of Halo+ tau biosensor cells pre-transfected with Halo-tagged FAF2 or FAF2_PS_, seeded with clarified tau brain homogenate, treated with DMSO, MG132 [5 μM] and BafA1 [50 nM] normalized to Halo control. Statistics between FAF2 or FAF2_PS_ treatment groups with one-way ANOVA with Tukey’s MCT. n=4. (**H**) Median YFP intensity as a measure of tau levels in the top 10% of Halo+ cells treated with DMSO, TAK-243 [0.2 μM], MG132 [5 μM] and BafA1 [50 nM] that are FRET+ normalized to Halo control. Unpaired t-test. Halo (DMSO) n=7, all others n=4. (**I**) IFD of top 10% of Halo+ tau biosensor cells pre-transfected with Halo, or FAF2_PS_-Halo fragments containing the hairpin (hp) domain or the UBA and UBX (UBA-UBX) domains as in Figure 2H,I. Cells were seeded with tau brain homogenate and treated with DMSO (n=5), TAK-243 [0.2 μM] (n=3) or MG132 [5 μM] (n=7) and normalized to Halo control. Statistics within Halo, hp or UBA-UBX groups with one-way ANOVA and Holm-Sidak’s MCT.

Since FAF2 is known to function as a VCP adaptor, we examined whether FAF2_PS_ effects on tau aggregation were mediated through VCP. Surprisingly, three observations demonstrated FAF2_PS_ suppresses tau aggregation independent of VCP. First, treatment of cells with VCP inhibitors (DBeQ, CB-5083 and NMS-873) did not reverse FAF2_PS_ suppression of tau aggregation and had varied, but non-significant, effects on FAF2 (Figure S6A). Second, knockdown of VCP by 90% with two siRNAs did not alter effects on tau aggregation (Figure 3A-B). Efficient VCP knockdown also did not alter tau levels (Figure S6B-D). Third, a UBX mutant with four point mutations (UBX_mut_) that prevent VCP binding did not alter effects on tau aggregation (Figure 3C,D).^23^ We validated expression and lack of changes in tau levels by Western blot (Figure S6E) as well as confirmed the loss of VCP-FAF2 interaction with deletion (ΔUBX) or mutation (UBX_mut_) of the UBX domain by co-immunoprecipitation (Figure S6F). Taken together, these observations support FAF2_PS_ suppression of tau aggregation is independent of VCP and suggest an alternative role for the UBX domain independent of VCP binding.

In addition to VCP, the Hsp70 chaperone network can also play a critical role in disaggregation and protein homeostasis in cooperation with additional cellular chaperones^28^. Thus, we evaluated whether the effect of FAF2_PS_ on tau aggregation required Hsp70 activity. We observed that FAF2_PS_ effects were not altered upon addition of Hsp70 inhibitors (Figure S6G) suggesting downstream mechanisms are also independent of Hsp70.

### FAF2/UBXD8 suppression of tau aggregation requires ubiquitination and proteasome function

As the UBA domain was required for FAF2_PS_ suppression of tau aggregation, we evaluated if ubiquitination is necessary. We treated cells at the time of tau seeding with TAK-243, an inhibitor of the E1 ubiquitin-activating enzyme (UAE) (Figure 3E). TAK-243 limited global ubiquitination without significantly altering tau levels (Figure S7A-C). TAK-243 reversed effects of FAF2_PS_ on tau aggregation when normalized to DMSO controls and showed a similar, but not significant trend, for FAF2 (Figure 3F). Thus, FAF2_PS_ requires active protein ubiquitination to prevent tau aggregation, consistent with the UBA domain acting through binding ubiquitinated proteins.

As FAF2_PS_ requires ubiquitination activity and reduces tau levels in cells with aggregates, we next examined if degradation is mediated by the proteasome or autophagy.^29^ We treated biosensor cells transfected with Halo control or FAF2 constructs with the proteasomal inhibitor MG132, or the autophagy inhibitor BafA1 at the time of seeding (Figure 3E). We validated that MG132 and BafA1 had anticipated effects on the levels of ubiquitinated proteins as well as autophagy markers p62 and LC3B without altering tau levels (Figure S7D-I).

Proteasomal inhibition led to a partial reversal of FAF2_PS_ effects on tau aggregation while autophagy inhibition had no impact (Figure 3G). Additionally, the reduction in tau levels – as monitored by the median YFP intensity – in FRET+ cells expressing FAF2_PS_ was reversed by MG132 and TAK-243 treatment but not by BafA1 relative to DMSO-treated controls (Figure 3H). Thus, FAF2_PS_ facilitates ubiquitin-dependent proteasomal degradation of tau in FRET+ cells, which contributes to the suppression of tau aggregation.

To assess domains of FAF2_PS_ acting through the ubiquitin-proteasome system, we expressed FAF2_PS_ fragments containing either the hairpin alone (hp) or the UBA and UBX domains (UBA-UBX) which can suppress tau aggregation (Figure 2I) with UAE and proteasome inhibition. Interestingly, we observed that UBA-UBX suppression of tau aggregation was reversed upon inhibition of ubiquitination or the proteasome while the effects of the hairpin were improved. These findings are consistent with FAF2_PS_ suppression of tau aggregation being mediated through at least two mechanisms with activity of the UBA-UBX domains being dependent on both ubiquitination and the proteasome while the hairpin domain can act independently through alternative mechanisms.

### Polyserine targeted FAF2/UBXD8 binds and prevents growth of tau seeds

**Figure 4.**
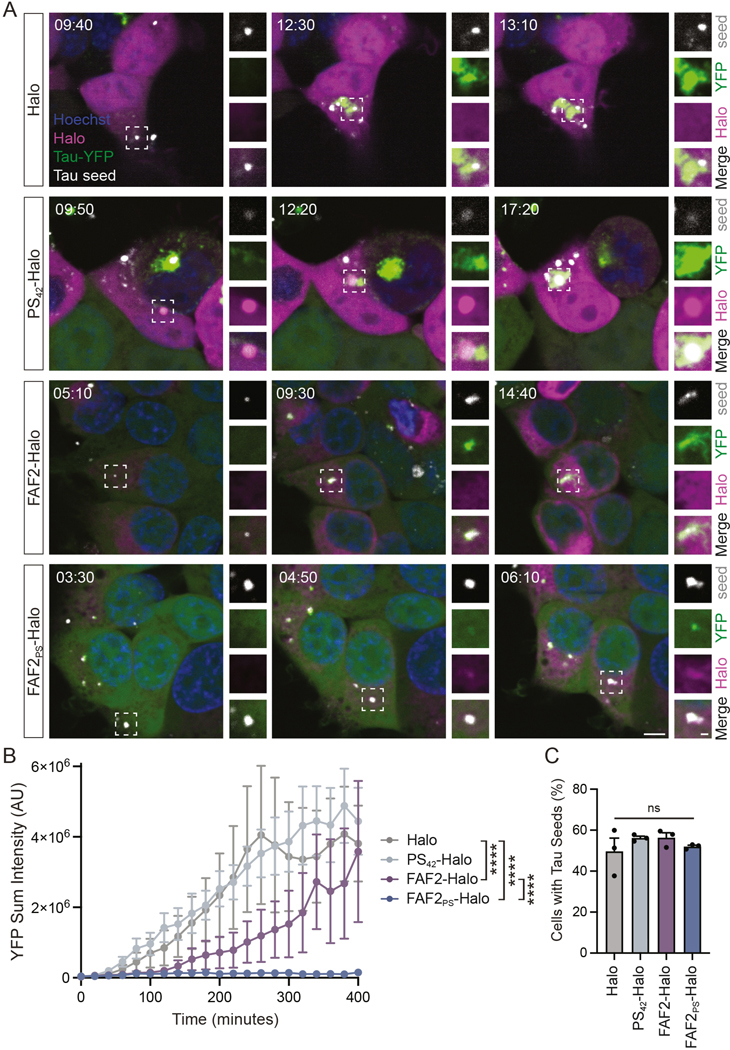
FAF2/UBXD8 prevents growth of tau aggregates while tau seeds persist. (**A**) Stills from live imaging experiments in tau biosensor cells transfected with Halo, PS_42_-Halo, FAF2-Halo or FAF2_PS_-Halo and fluorescently labeled tau seeds. (**B**) Quantification of YFP sum intensity of a tau aggregate in Halo+ cells transfected with constructs as in (A) with T=0 defined as the first frame where a tau seed enters cell. Two-way ANOVA with Tukey’s MCT. n=10 cells per condition. (**C**) Percent of HEK293T WT cells containing fluorescently labeled tau seeds 24 hours post-seeding by flow cytometry in top 10% of Halo+ cells pre-transfected with constructs as in (A). One-way ANOVA with Tukey’s MCT. n=3.

To further examine how FAF2_PS_ disrupts the process of tau aggregation, we performed live imaging in tau biosensor cells transfected with fluorescently labeled tau seeds generated from fibrillized recombinant tau (Figure 4A). We observe tau seeds enter cells and nucleate aggregation of YFP-tau which accumulates over time and remains associated with the exogenous seed (Figure 4A) (Video S1). Consistent with our previous reports, cells expressing polySer form self-assemblies that associate with aggregating tau (Figure 4A).^16^ Further underlining the association of pathogenic tau and polySer domains we observe that polyserine assemblies enrich tau seeds (Figure 4A) (Video S2).

To determine how FAF2 may affect rates of tau aggregate growth, we quantified the sum intensity of YFP-tau in an aggregate associated with a labeled tau seed following cell entry. We observed a striking effect where tau seeds in cells expressing FAF2_PS_ did not appreciably grow or incorporate YFP-tau beyond the area of the seed itself (Figure 4B). This inhibition is coincident with FAF2_PS_ co-localizing with tau seeds (Figure 4A) (Video S3). FAF2 also reduces the rate of tau aggregate growth, but not as effectively as FAF2_PS_ (Figure 4B). The reduced effect of FAF2 is potentially due to reduced association with tau seeds as FAF2 enrichment with exogenous seeds is not observed without polySer targeting (Figure 4A,B) (Video S4).

Interestingly, we observed labeled tau seeds persist throughout the length of this analysis indicating FAF2_PS_ does not increase turnover of tau seeds but rather prevents seeds from nucleating further aggregation in the cytoplasm. To investigate this without the confounding factor of seeded tau aggregates, we performed flow cytometry on wild-type HEK293T cells transfected with control or FAF2 constructs 24 hours prior to seeding with fluorescent seeds. With filtering for the highest 10% of Halo expressing cells we observed no differences in the number of cells with tau seeds 24 hours after lipofection (Figure 4C).

Taken together, these results show polySer enables interaction with tau seeds where FAF2_PS_ prevents growth of an aggregate by limiting incorporation of cellular tau.

### FAF2/UBXD8 suppresses degenerative phenotypes in Drosophila eye

**Figure 5.**
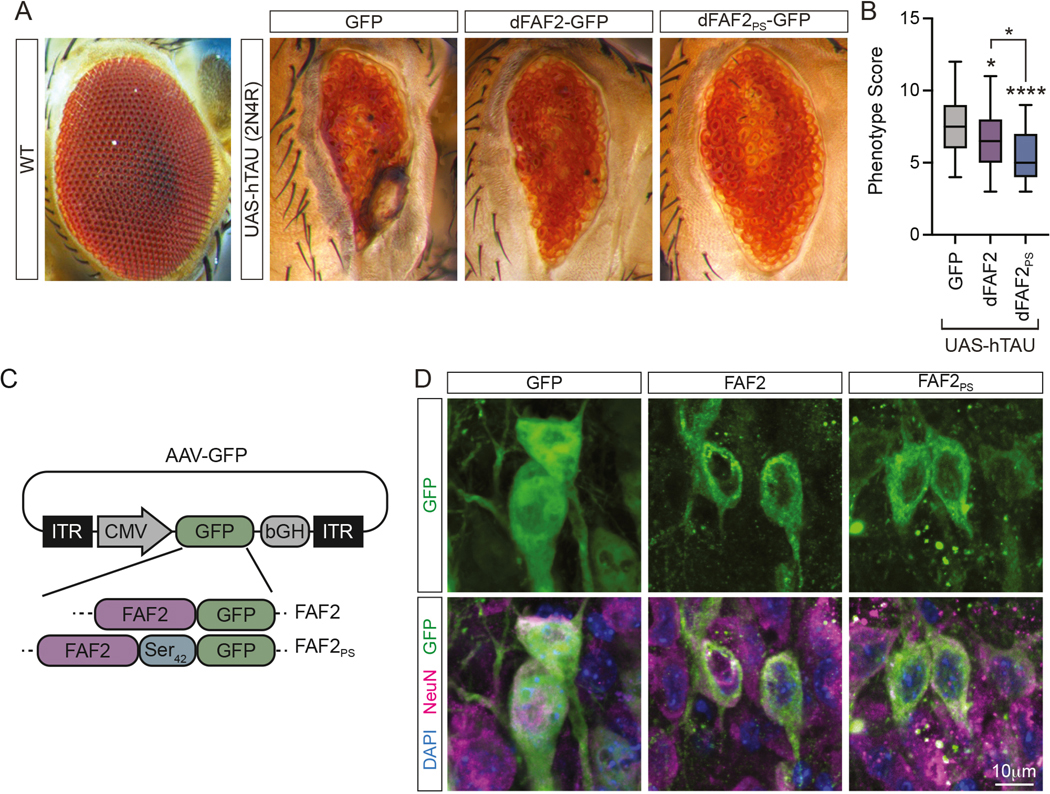
FAF2/UBXD8 suppresses degeneration in a Drosophila tau transgenic model and can be delivered by AAV9 in a mouse model. (**A**) Representative images of Drosophila eyes from one-day old flies ectopically expressing human 2N4R tau and either GFP, FAF2-GFP or FAF2_PS_-GFP. (**B**) Quantification of severity of eye degeneration for groups in (A) as mean phenotypic score calculated based on presence and severity of pre-defined morphological aberrations (see Methods). One-way ANOVA and Tukey’s MCT. GFP n=32, dFAF2-GFP n=40, dFAF2–42PS-GFP n=46. (**C**) AAV vectors used to deliver GFP, FAF2-GFP and FAF2_PS_-GFP to WT and PS19 tau transgenic mice. (**D**) Immunostaining of GFP (green), NeuN (magenta) and DAPI (blue) in the CA3 hippocampal region of 6-month-old PS19 tau transgenic mice injected with AAV9 as in (C).

We next tested whether the effects of FAF2_PS_ on tau aggregation in cells were relevant in animal models of disease. Drosophila not only show tau-induced neurodegeneration, but also have conserved homologs of FAF2/UBXD8.^30–33^ We created transgenic Drosophila lines with Gal4-responsive expression of GFP, Drosophila FAF2 (dFAF2)-GFP or dFAF2_PS_-GFP under regulation of the Upstream Activation Sequence (UAS) and combined these with a previously characterized UAS-2N4R WT human tau line (Figure 5A).^30^ Following crossing of each double UAS-transgene line with an eye-specific GMR-GAL4 line to induce expression of GFP, dFAF2, dFAF2_PS_ and human tau in the fly eye, western blot analysis validated expression of transgenes and showed no significant differences in phosphorylated (AT8 and AT180) or total tau levels across lines (Figure S8A-H). Next, we examined the effect of FAF2 targeting on tau-induced degenerative phenotypes in the fly eye utilizing an adapted scoring system^34^ (Figure S8I). We observed that dFAF2 expression partially rescued tau-driven neurodegeneration, which was further improved with polySer targeting (Figure 5B). Thus, FAF2 can reduce tau-mediated neurodegeneration in an animal model of disease.

### AAV9-mediated expression of polyserine targeted FAF2/UBXD8 in a mouse tauopathy model

To determine whether FAF2 activity towards tau aggregation was effective in a mammalian model of tauopathy, we used AAV9 to deliver transgenes to wild-type (WT) or tau transgenic mice that harbor one copy of a transgene expressing 1N4R human tau with a P301S mutation driven by the mouse prion promotor (PS19).^35^ We generated AAV9 encoding GFP as a negative control as well as GFP-tagged FAF2 and FAF2_PS_ which we delivered at postnatal day 1 (P1) by intracerebroventricular (ICV) injection (Figure 5C).

To quantify transgene expression, we performed RT-qPCR on RNA isolated from spinal cord and detected comparable levels across groups using GFP or human FAF2 specific primers (Figure S9A,B). Importantly, we detected no differences in hMAPT mRNA levels indicating AAV-delivery and expression does not alter tau transgene expression (Figure S9C). We also performed immunostaining and observed GFP and NeuN positive hippocampal neurons demonstrating AAV9 successfully transduced neuronal subtypes of high relevance for tau pathology in PS19 mice (Figure 5D, S9D,E).

We next monitored WT and PS19 animals injected with AAV9 to assess any overt toxicities associated with expression. AAV9-injected animals did not show significant changes in survival, alterations in weight, or consistent changes in accelerating rotarod performance with AAV9-FAF2 or AAV9-FAF2_PS_ relative to AAV9-GFP animals from four to ten-months-old (Figure S10A-F). This demonstrates fusion of polySer to FAF2 prevents impariments in weight and rotarod performance previously described due to overexpression of polySer alone.^17^ Furthermore, we did not observe Purkinje cell loss or changes in cross-sectional area of cerebellum with AAV9-FAF2_PS_ in contrast to loss of these neurons observed with polySer alone at six-months-old (Figure S11A,B).^17^

Collectively, these results support AAV9 effectively transduces relevant hippocampal neurons and fusion of polySer to FAF2 neutralizes toxic effects of polySer alone.

### FAF2/UBXD8 suppresses features of tau pathology in PS19 tau transgenic mice

We next evaluated effects of AAV9-delivery on pathological features of the PS19 mouse model.

**Figure 6.**
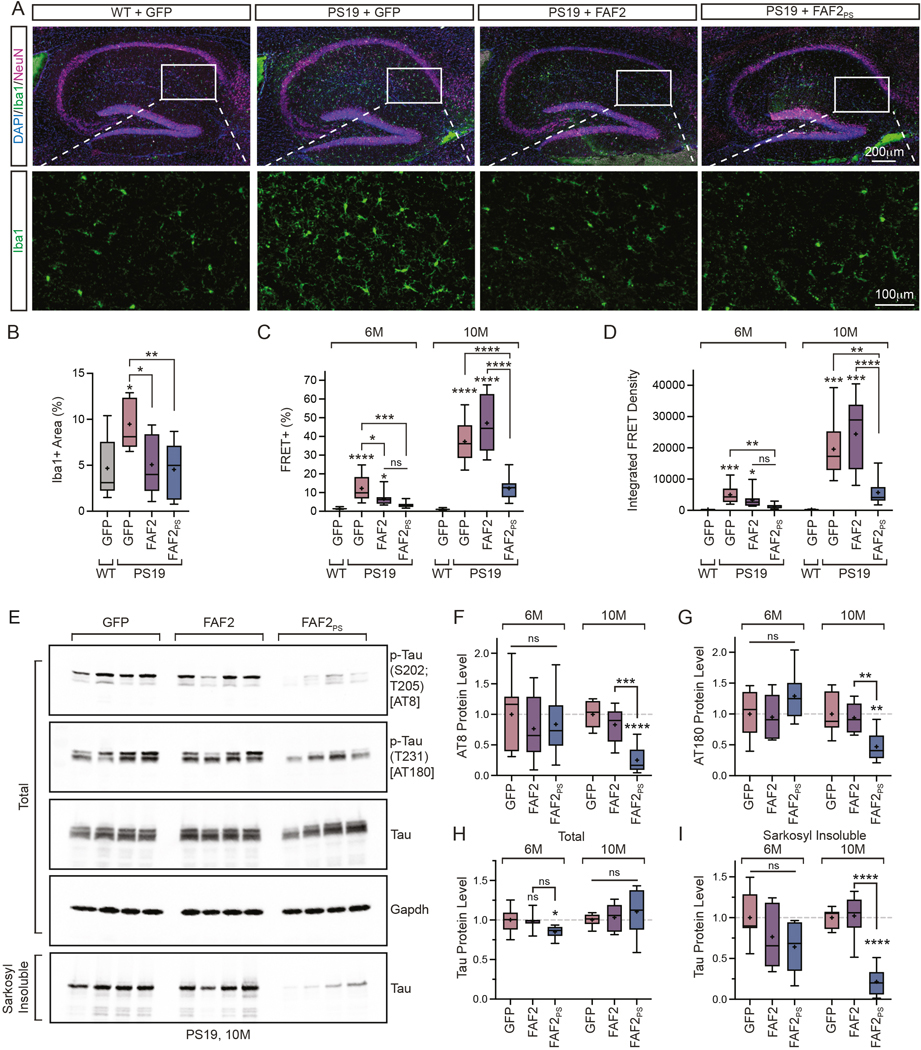
AAV9-mediated delivery of polyserine FAF2 suppresses pathology and seeding in a mouse tauopathy model. (**A**) Immunostaining of DAPI (blue), Iba1 (green) and NeuN (magenta) in hippocampi of WT or PS19 animals injected with AAV9-GFP, AAV9-FAF2 or AAV9-FAF2_PS_. (**B**) Quantification of percent area of Iba1 coverage in hippocampus of groups in (A). One-way ANOVA and Tukey’s MCT. n=3 sections per animal averaged for n=9 animals per group. (**C**) Percentage of FRET+ tau biosensor cells via flow cytometry transfected with sarkosyl-insoluble brain extracts from 6-month (6M) or 10-month (10M) old animals in groups as in (A). One-way ANOVA and Tukey’s MCT for each timepoint. 6M: n=9 animals per group. (**D**) IFD by flow cytometry of tau biosensor cells transfected with sarkosyl insoluble brain extracts as in (C). One-way ANOVA and Tukey’s MCT for each timepoint. n=9 animals per group. (**E**) Western blot of phosphorylated tau (AT8 and AT180), total tau and GAPDH in total brain extracts and total tau in sarkosyl-insoluble extracts from AAV9-GFP, AAV9-FAF2 or AAV9-FAF2_PS_ injected PS19 mice at 10-months-old. (**F-I**) Quantification of AT8 phospho-tau (F), AT180 phospho-tau (G), total tau (H) or sarkosyl insoluble tau (I) protein levels normalized to GAPDH as in (E) and Figures S12A-C relative to GFP control. One-way ANOVA and Tukey’s MCT for each timepoint. n=8 animals per group.

Previous characterization reported increased microglial activation in the hippocampus of PS19 animals as an early feature of the disease model.^35^ We quantified the percent area of the hippocampus with positive for the microglial marker Iba1 at six-months-old (Figure 6A).^36^ Consistent with prior findings, we observed increased microglia in GFP-injected PS19 animals compared to WT (Figure 6B). Importantly, we observed Iba1+ microglia were significantly reduced in FAF2 and FAF2_PS_ animals, indicating reduced neuroinflammation (Figure 6B).

Another marker of tau pathology is seeding activity of brain extracts.^21^ We performed sarkosyl fractionation on extracts from AAV9-injected animals at six and ten-months-old. Next, we lipofected total, sarkosyl soluble and sarkosyl insoluble fractions into tau biosensor cells and measured seeding capacity by flow cytometry. As expected, we did not observe seeding activity in samples from WT animals (Figure 6C,D, S11C,D). Additionally, while we observed FRET+ tau aggregates in cells transfected with total PS19 extracts they were absent from soluble fractions validating that seeding-competent species were isolated in insoluble fractions (Figure S11C,D).

Strikingly, at six-months-old we observed both total and sarkosyl insoluble fractions from FAF2_PS_ treated PS19 mice showed reduced FRET+ cells and IFD (Figure 6C,D, S11C). We also observed a reduction in FRET+ cells from FAF2 animals (Figure 6C). At ten-months-old, FAF2_PS_ animals maintained reduced seeding activity in total and sarkosyl insoluble brain extracts relative to GFP controls (Figure 6D, S11D). Further, at this later disease stage, seeding activity in FAF2_PS_ animals was significantly lower than FAF2 highlighting the role of polySer in enhancing effects of FAF2 on limiting seeding-competent tau species.

Lastly, to directly assess potential changes in tau, we analyzed protein levels of tau in brain extracts of PS19 animals at six and ten-months-old (Figure 6E, S12A-C). We monitored phosphorylated tau with AT8 and AT180 antibodies and detected no changes at six-months-old, but did observe pronounced reduction at ten-months-old specifically in FAF2_PS_ animals (Figure 6F,G). At six-months-old there was a small reduction in total tau levels in FAF2_PS_ treated animals which was not persistent at ten-months-old (Figure 6H). Lastly, while there were no significant changes at six-months-old, by ten-months-old we observed a dramatic reduction in sarkosyl insoluble tau levels in FAF2_PS_ brains (Figure 6I).

**Figure 7.**
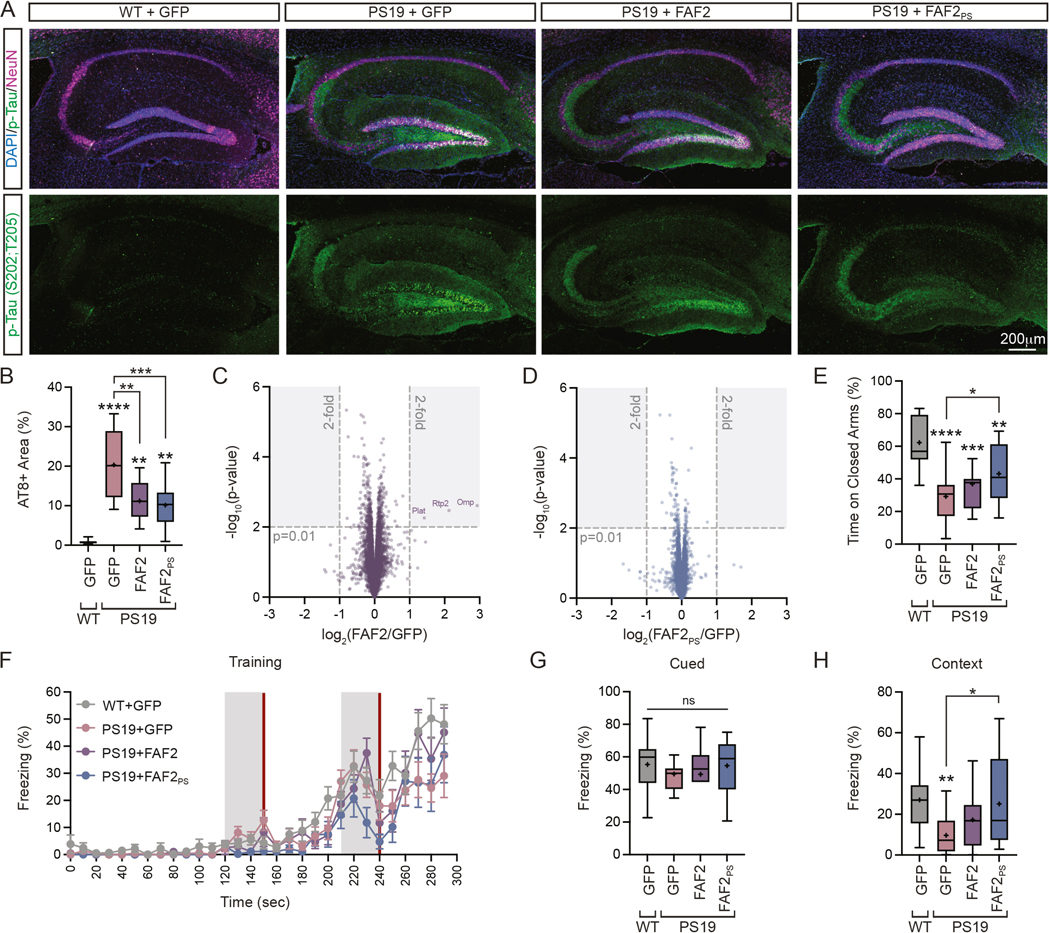
Polyserine-targeted FAF2/UBXD8 reduces tau levels and improves contextual fear conditioning response in a mouse tauopathy model. (**A**) Immunostaining of DAPI (blue), phosphorylated tau (AH36) (green) and NeuN (magenta) in hippocampi of WT or PS19 animals injected with AAV9-GFP, AAV9-FAF2 or AAV9-FAF2_PS_. (**B**) Quantification of AH36+ hippocampal area as in (A). One-way ANOVA and Tukey’s MCT. n=3 sections per animal averaged for n=10 animals per group. (**C**) Volcano plot of protein expression by Tandem mass tag pro (TMTpro) mass spectrometry in brain extracts from AAV9-FAF2 animals compared to AAV9-GFP at 6-months-old. Shaded areas represent fold change >2 and p-value >0.01. (**D**) Volcano plot of protein expression by TMTpro mass spectrometry in brain extracts from AAV9-FAF2_PS_ animals compared to AAV9-GFP at 6-months-old. Shaded areas represent fold change >2 and p-value above 0.01. (**E**) Percent of time spent on closed arms during elevated plus maze assay for 10-month-old animals as in (A). Boxplot whiskers by Tukey’s method. One-way ANOVA. WT+GFP(n=16), PS19+GFP(n=18), PS19+FAF2(n=11), PS19+FAF2_PS_(n=12). (**F**) Percent freezing during training period of fear conditioning assay for animals as in (E). Grey boxes denote cued stimulus (tone) and red line denotes aversive stimulus (foot shock). WT+GFP(n=18), PS19+GFP(n=19), PS19+FAF2(n=11), PS19+FAF2_PS_(n=11). (**G**) Average percent of time spent freezing during periods of cued stimulus for animals as in (F). Boxplot whiskers by Tukey’s method. One-way ANOVA and Tukey’s MCT. WT+GFP(n=18), PS19+GFP(n=19), PS19+FAF2(n=11), PS19+FAF2_PS_(n=11). (**I**) Average percent of time spent freezing during contextual testing for animals in (F). Boxplot whiskers by Tukey’s method. One-way ANOVA and Tukey’s MCT. WT+GFP(n=18), PS19+GFP(n=19), PS19+FAF2(n=11), PS19+FAF2_PS_(n=11).

Next, to gain more precise analysis of a specific brain region, we performed immunostaining and quantification of phosphorylated tau (S202,T205) in the hippocampus. Here we observed a significant increase in GFP-injected PS19 animals relative to WT controls which was effectively corrected with both FAF2 and FAF2_PS_ (Figure 7A,B).

Collectively, these findings demonstrate FAF2_PS_ expression reduces key features of tau pathology *in vivo*. Further, consistent with results in cellular models, FAF2 has some suppressive effects on tau pathology, but these are greatly enhanced with polySer-based targeting.

### FAF2/UBXD8 expression does not induce widespread proteomic changes in the mammalian brain

To assess the specificity of FAF2 and FAF2PS expression, we performed mass spectrometry analysis of brain homogenate from AAV-injected animals at six-months-old (Table S1). Analysis of over 7500 proteins showed 3 which changed upon FAF2 expression relative to GFP controls meeting a cutoff of a 2-fold change and p-value >0.01 (Figure 7C) and none in FAF2_PS_ expressing animals (Figure 7D). Thus, FAF2_PS_ expression does not induce widespread proteomic changes *in vivo*.

### FAF2/UBXD8 improves elevated plus maze and contextual fear conditioning responses in tau transgenic mice

We next set out to assess whether corrected measures of tau pathology in PS19 mice by FAF2_PS_ were accompanied by behavioral and cognitive improvements.

First, we tested ten-month-old animals on the elevated plus maze assay which has been previously reported to display tau transgene-dependent changes in performance^37^. While we observed no differences in distance moved (Figure S12D), we saw a significant reduction in the time spent on closed arms in PS19 GFP-injected animals relative to WT (Figure 7E). Further, we observed a significant increase in FAF2_PS_ treated animals indicating correction of the reduced anxiety phenotype observed in PS19 mice at late stages.

Next, to evaluate associative learning, we performed fear conditioning in WT and PS19 animals which have been previous shown to display impairments in contextual fear memory.^37–40^ First, we observed responses across all AAV9-injected groups to paired auditory cues and electric foot shock during the training period (Figure 7F) suggesting learning was comparable and allowing assessment of memory dependent effects. The subsequent day, we analyzed fear memory through freezing response to cued and contextual stimuli, which largely depend on the amygdala and hippocampus, respectively.^41–43^ We observed no significant differences across groups in the freezing response of animals prior to cued stimuli (Figure S12E) or during cued stimuli consistent with applicable brain regions being less affected by tau pathology in this model (Figure 7G). Importantly, in context testing – which is impaired in GFP injected PS19 animals relative to WT – we observed a significant increase in the freezing response of FAF2_PS_ treated mice relative to PS19 GFP controls (Figure 7H).

Thus, FAF2_PS_ delivery rescues reduced anxiety as well as contextual fear conditioning response – a measure of hippocampus-dependent memory in tau transgenic mice. These effects are consistent with a functional benefit to tau transgenic animals upon FAF2_PS_ expression concurrent with the attenuation of tau pathology.

## Discussion

In this study we demonstrate a polySer domain can be used to target multiple proteins – including VCP, TNPO3, and FAF2 – to tau aggregates and thereby reduce their formation (Figure 1,2). This identifies a new and flexible approach for probing the biology of tau aggregation and potentially developing new therapeutic approaches by increasing the effectiveness of proteins that inhibit tau aggregation. For example, while overexpressed FAF2 can suppress tau aggregation, this activity is strongly increased by the addition of a polySer targeting module (Figure 2). This approach is agnostic to the cargo used as all proteins we have tagged with polySer enrich in tau aggregates and a polySer tag is functional when located at the N or C terminus giving flexibility in protein design (Figures 1,2).

FAF2 emerges as a potent suppressor of tau aggregation demonstrating effectiveness in both tau biosensor cells and in animal models of disease. We observed FAF2_PS_ reduced tau aggregation by approximately 85% in cell models, with untagged FAF2 having a more modest effect (Figure 2). Consistently, others have reported that FAF2 knockout in biosensor cells leads to increased tau aggregation.^44^ Furthermore, overexpression of Drosophila FAF2 in flies – with or without polySer targeting – effectively mitigated degeneration driven by a human tau transgene (Figure 5). Most significantly, we observe AAV9 delivery of FAF2_PS_ in the PS19 mouse model attenuated microgliosis, reduced phosphorylated and insoluble tau levels, hindered the production of tau seeds, restored decreased anxiety and conferred a cognitive benefit in contextual fear conditioning consistent with improved hippocampal function (Figure 6 and 7). While FAF2 alone can rescue select defects such as microgliosis and seeding capacity at early stages, the addition of polySer increases efficacy. Collectively, these observations underscore the effectiveness of FAF2_PS_ in reducing tau-driven pathology *in vivo*.

Our results suggest FAF2_PS_ can work through multiple mechanisms to reduce tau aggregation. First, we observe cells that express FAF2_PS_ and contain tau aggregates have reduced tau levels through a mechanism driven by ubiquitination and proteasomal degradation (Figure 3). This could occur by FAF2_PS_ promoting interaction of the proteasome with ubiquitinated tau. However, we cannot rule out the possibility that ubiquitination of a different protein indirectly leads to FAF2_PS_ dependent degradation of tau. Importantly, suppressive activity of the UBA and UBX domains require ubiquitination and proteasome function, while the hairpin domain does not and can act via an independent mechanism that contributes to overall suppression of tau aggregation. (Figure 3). Additionally, live cell imaging suggests that FAF2_PS_ interacts with tau seeds when they first enter the cell and prevents further growth in a manner augmented by polySer targeting (Figure 3). We hypothesize that limiting aggregate growth at a tau seed can also be mediated – at least in part – by a ubiquitination-dependent proteasomal degradation of tau and may be further driven by an additional direct effect on tau fibrillization.

Aligned with this mechanism of action, we observe that FAF2_PS_ is a potent suppressor of tau aggregation, although it cannot measurably reverse existing aggregates (Figure S4). Therefore, a cell expressing FAF2_PS_, when encountering a tau seed, can sequester that seed and prevent incorporation of additional tau. However, in a cell with higher tau aggregate burden, subsequent expression of FAF2_PS_ can promote degradation of tau via the proteasome but appears unable to outcompete the rates of tau aggregate growth in our experimental paradigm. These findings underscore the importance of early intervention with FAF2_PS_ and additional studies are required to identify the precise timing required for efficacy of FAF2_PS_ if delivered at later stages of disease in tau transgenic models.

One intriguing aspect of our study is the discovery that FAF2_PS_ can limit tau aggregation independently of VCP. This finding is strongly supported by our observations showing that FAF2_PS_ inhibition of tau aggregation remains unaffected by VCP knockdown, chemical inhibition, or point mutations in the FAF2 UBX domain that abolish VCP binding (Figure 3). Notably, when compared to several other VCP adaptor proteins, only UBXD1 and FAF2 showed suppressive activities towards tau when overexpressed underlining the distinct mechanisms of action these proteins may have towards tau and that there is not a generalized effect of VCP adaptors on tau aggregation. Taken together, these observations point to a VCP independent function of FAF2, potentially involving interaction with other yet to be identified proteins to limit tau aggregation.

Collectively, we identify polySer as an effective targeting strategy – and FAF2 as a suppressor of tau aggregation that is effective in both cell and animal models of tau pathology. Importantly, we and others have previously shown that the polySer containing protein SRRM2 is mislocalized to tau aggregates in tau transgenic mice as well as in corticobasal degeneration and Alzheimer’s disease patient tissue indicating conserved associations between polySer and tau.^15,45^ Future work investigating the mechanism driving polySer and tau associations will allow for fine-tuning of targeting functions and potential separation of the pro-pathogenic effects of polySer alone. Additionally, further evaluation of the suppressive activity of FAF2 in tau models across different treatment paradigms will provide crucial insight into potential therapeutic utility.

### Resource Availability

#### Lead contact

Requests for further resources should be directed to and will be fulfilled by the lead contact, Roy Parker (Roy.Parker@Colorado.edu)

#### Materials availability

All unique/stable reagents generated in this study are available from the Lead Contact with a completed Materials Transfer Agreement.

#### Data and code availability

Data reported in this paper will be shared by the lead contact upon request. This paper does not report original code. Any additional information required to reanalyze the data reported in this paper is available from the lead contact upon request. The mass spectrometry proteomics data have been deposited to the ProteomeXchange Consortium via the PRIDE^46^ partner repository with the dataset identifier PXD066746 and 10.6019/PXD066746.

## STAR Methods

### EXPERIMENTAL MODELS

#### Cell Lines

HEK293T tau biosensor female cells stably expressing the 4R repeat domain of tau (K18) with the P301S mutation fused to CFP and YFP were purchased from ATCC (CRL-3275).^21^ HEK293T WT female cells were purchased from ATCC (CRL-3216). As previously described, cells were grown in 10% FBS, 0.2% penicillin-streptomycin at 37°C with 5% carbon dioxide.

#### Drosophila models

Fly crosses and experiments were performed at 21°C and 60% humidity with a 12h light-dark cycle. The GMR-GAL4 (BDSC_1104) retinal driver line was obtained from the Bloomington Drosophila Stock Center (Bloomington, IN). The UAS human Tau 2N4R line was a generous gift from G. Tear (King’s College London, UK).^30^ To generate transgenic lines, the ORFs of GFP, Drosophila FAF2-GFP and Drosophila FAF2_PS_-GFP were synthesized (Twist) and cloned into the pJFRC-MUH plasmid (Addgene #26213).^47^ Transgenic flies were subsequently created using the attP40 landing site (NCBS fly facility) and analyzed at one-day old.

#### Mouse animal procedures

Mice were housed in climate-controlled vivarium with 12:12: Light:Dark cycles. Food and water were provided ad libitum. All procedures with mice were performed in accordance with the National Institutes of Health Guide on the Care and Use of Animals and approved by the Institutional Animal Care and Use Committee of University of Colorado Boulder. Female and male mice were used across each treatment group and aggregated data is presented as gender-specific differences were not observed. PS19 tau transgenic mice on a C57BL/6-congenic background were obtained from Jackson mice (Strain #024841) and crossed with C57BL/6J (Strain #000664) to obtain WT or heterozygous experimental animals. Genotyping was performed using DNA extracted from tails and with primers listed in the Key Resources Table. Animals were tested in behavioral assays from four to ten-months-old and tissue was collected and analyzed at six or ten-months-old.

### METHOD DETAILS

#### DNA Constructs

Fusion proteins and controls were cloned using In-Fusion Cloning Kit (Takara) into a pcDNA3.1 plasmid backbone with a CMV promoter (Invitrogen) and modified with a puromycin selection marker. To generate FAF2 VCP-binding deficient mutant, multiple rounds of site directed mutagenesis were performed with QuikChange II Site-directed mutagenesis kit (Agilent). Gene fragments for FAF2 deletion mutants or fragments were synthesized (Twist) then cloned into the pcDNA3.1 plasmid backbone.

#### Cell culture treatments

For tau seeding, clarified brain homogenate from Tg(Thy1-MAPT*P301S)2541 tau transgenic mice^48^ was prepared as previously described^15,16^. Tau brain homogenate was transfected into cells with Lipofectamine3000 (Invitrogen). For flow cytometry experiments tau brain homogenate was seeded at a final concentration of 0.5 ng/μl. For fluorescence imaging experiments tau brain homogenate was seeded at a final concentration of 1.75 ng/μl.

For re-infection experiments HEK tau biosensor cells were plated in 6-well format, transfected with plasmid (2.5μg/well) and seeded with clarified tau brain homogenate (0.5ng/μl) with 24 hours between treatments and collection. Collected cell pellets were resuspended in PBS supplemented with protease inhibitor (Roche) and phosphatase inhibitor (Roche) and lysed with a 25G needle prior to a 1000xg spin for 3 minutes to remove cell debris. The supernatant was quantified with Bradford assay and used for seeding experiments at a final concentration of 1μg/24-well prior to flow cytometry.

For compound treatments stock concentrations were made to allow for a final DMSO concentration of 1%. Compounds were added at the following concentrations: TAK-243 [0.2 μM] (MedChem Express), DBeQ [5 μM] (Fisher), CB-5083 [2.5 μM] (MedChem Express), NMS-873 [2 μM] (MedChem Express), MG132 [5 μM] (Sigma) and BafA1 [50 nM] (Cel Signaling).

For siRNA treatments, HEK293T biosensor cells were plated in a 6-well format and transfected with 25pmol siRNA per well with Lipofectamine RNAiMAX (Invitrogen) the following day. 24-hours post siRNA treatment, cells were replated in 24-well format for plasmid transfection and tau seeding. For validation of siRNA knockdowns, HEK293T biosensor cells were plated in a 6-well format and transfected with 25pmol siRNA per well the following day and collected for analysis 48 hours later.

For labeling of Halo proteins in cells TMRDirect (for flow cytometry experiments in tau biosensor cells), Janelia Fluor 503 (for flow cytometry experiments in HEK293T WT cells) and Janelia Fluor 646 (for fluorescence imaging) Halo ligands [200nM] (Promega) were added to culture media 24 hours prior to analysis.

#### Flow cytometry

Flow cytometry was performed as previously reported.^16^ Briefly, HEK293T tau biosensor cells were transfected with 500ng of plasmid and seeded with clarified tau brain homogenate (0.5 ng/μl). 24 hours post-seeding, cells were trypsinized, washed with PBS, and filtered with 50μm nylon mesh filters prior to cell sorting. Sorting was performed with a BD FACSCelesta^™^ Cell Analyzer using the following filter sets: 561–585 (Halo), 405–450 (CFP), and 405–525 (FRET). Analysis was performed using FlowJo. Gating was performed in sequential steps, first sorting for cells, single cells, then (when applicable) gating based on Halo expression was performed. Lastly, gating for FRET+ cells was performed based on mock seeded cells to set a false FRET percentage at 1 as previously detailed.^49^ Integrated FRET Density (IFD) was calculated as a product of the percentage of FRET-positive cells and median fluorescence intensity.

#### Immunoprecipitation

Halo immunoprecipitation was performed with Halo-Trap Agarose beads (ChromoTek) according to manufacturer’s instructions. Briefly, samples were lysed in buffer containing 0.5% NP-40 and immunoprecipitation was performed using beads pre-blocked with 3% BSA in buffer containing 0.1% NP-40 supplemented with protease and phosphatase inhibitors (Roche).

#### Western blot

For Western blotting, cell pellets were lysed in 2X SDS loading buffer, passed through a 25G syringe, and boiled. Protein extracts were run on 4–12 or 4–20% pre-cast Tris-Glycine gels (Thermo Fisher). Gels were then transferred to nitrocellulose membranes using the iBlot 2 Gel Transfer Device (Invitrogen). After transfer, membranes were blocked in 5% milk or BSA in Tris-buffered Saline with 0.1% Tween (TBS-T) for 1 hour, incubated with primary antibodies in TBS-T for 2 hours at room temperature, washed 3 × 10 minutes with TBS-T, incubated with secondary antibodies in TBS-T for 1 hour at room temperature, then washed 6 × 5 minutes with TBS-T before developing with Clarity Western ECL Substrate (Bio-Rad). Blots were imaged with iBright Imaging System (Thermo Fisher).

#### Fluorescence imaging of cultured cells

At the time of collection cells were fixed for 15 minutes in 4% paraformaldehyde in PBS, then permeabilized with 0.5% Triton-X for 10 minutes. Cells were then blocked in 3% BSA/0.2% sodium azide for 1 hour prior to staining with DAPI for 1 hour. After 3x5 minute washes, cells were then mounted with ProLong Glass (Thermo Fisher).

#### Immunofluorescence

For staining of brain sections, tissue was post-fixed for 24 hours with 4% methanol-free paraformaldehyde in PBS then protected with 30% sucrose prior to embedding in optimal cutting temperature (O.C.T.) compound and flash freezing. 30μm sagittal sections were prepared using a cryostat and O.C.T. compound removed with PBS prior to blocking with 10% donkey serum (Millipore) in TBS-T (0.4% Triton-X). Staining with primary and secondary antibodies in blocking buffer was performed before mounting with FluoromountG (Electron Microscopy Sciences).

#### Recombinant tau purification, labeling and fibrillization

A pET29b bacterial expression plasmid for 2N4R P301S, I260C, C291A, C322A hTau (modified from Addgene #108867 to include C-terminal 6xHis tag and P301S mutation) was transformed in Rosetta 2(DE3) pLysS E. coli (Sigma). One liter LB cultures were inoculated with overnight cultures and grown to O.D 600. Protein expression was induced with 200 μM IPTG for 4 hours at 37°C. Bacterial cell pellets were harvested at 4000xg and stored at −80°C.

Bacterial cell pellets were lysed in Lysis Buffer (50 mM Tris pH 7.5, 500 mM NaCl, 30 mM imidazole pH 8.0, 1 mM DTT) by sonication. The lysate was clarified via centrifugation at 35000xg for 30 minutes then filtered through 2.7 μm syringe filters and loaded onto pre-equilibrated Ni-NTA resin. The column was washed with 5 column volume (CV) of Lysis Buffer, 10 CV of Wash Buffer 1 (50 mM Tris pH 7.5, 200 mM NaCl, 30 mM imidazole pH 8.0, 1 mM DTT), and 10 CV of Wash Buffer 2 (50 mM Tris pH 7.5, 1M NaCl, 30 mM imidazole pH 8.0, 1 mM DTT). Column was incubated in 6 CV of Elution Buffer (50 mM Tris pH 7.5, 200 mM NaCl, 300 mM imidazole pH 8.0, 1 mM DTT). All buffers were supplemented with protease inhibitor (Roche). Eluted protein was dialyzed for three hours at room temperature into Buffer A (50 mM MES pH 6.0, 50 mM NaCl, 1 mM DTT), followed by overnight dialysis.

Next, protein was purified on an SP cation exchange column (Cytiva) using an AKTA FPLC eluting with a gradient of Buffer B (50 mM MES pH 6.0, 1M NaCl, 1 mM DTT). Tau containing fractions were pooled and concentrated to less than 1 mL and loaded onto an S200 16/600 size exclusion chromatography column (Cytiva). Protein was eluted with 1.5 CV of Maleimide Elution Buffer (1x PBS pH 7.4, 1 mM TCEP), fractions pooled and concentrated to less than 500 μL.

For labeling, a three times molar excess of Cy3-Maleimide dye was added to 500 μL of purified tau for two hours at room temperature then dialyzed overnight at 4°C into Fibrilization Buffer (1x PBS pH 7.4, 1 mM DTT).

Tau fibers were prepared using 5 μM Cy3-Maleimide labeled tau incubated with 400 ng/μL of polyU RNA (Sigma-Aldrich), in fibrilization buffer supplemented with freshly prepared 1 mM DTT. The reaction was shaken at 400 RPM in an Eppendorf thermomixer for 24 hours. Fibers were purified from non-fibrilized tau via centrifugation at 100k × g for 30 minutes, washed with PBS and pelleted again at 100k × g for 30 minutes. The final protein pellet was resuspended in PBS and stored at −80°C prior to transfection.

#### Drosophila phenotyping

For the phenotypic analysis, eye phenotypes were photographed and scored on day 0–1 post eclosion according to a scoring system adapted from Pandey et. al.^34^ All scoring was done by individuals blind to fly genotype. Morphological aberrations were divided into 5 categories consisting of discolouration, ommatidia phenotypes, shrinkage/narrowing of the eye’s surface area, collapse of the eye’s convex surface, and necrotic lesions. Flies were given one point for discolouration, one point for disorganization of the ommatidial array, and two points for ommatidial fusion. For the eye shrinkage, eye collapse, and necrotic lesion categories flies were scored between 1 and 3 depending on the severity of these aberrations. Subsequently, individual flies could score between 0 and 12 with higher scores indicating more severe phenotypes. Eye image stacks were captured using a Leica M205 FCA stereomicroscope and a Leica DMC 6200 digital camera with a 10x lens and a stack distance of 18μm.

#### Drosophila western blots

40 fly heads of 1-day old flies per genotype were collected on ice and transferred into 40μl pre-cooled extraction buffer (20mM HEPES, pH 7.5, 100mM KCl, 5% glycerol, 10mM EDTA, 0.1% Triton, 1mM dithiothreitol, 0.5mM phenylmethylsulfonyl fluoride, 20mg/ml aprotinin, 5mg/ml leupeptin, 5mg/ml pepstatin A). Heads were ground with a pestle for 30s then briefly centrifuged at 4°C a total of three times, before being centrifuged for 5 min at 4°C. The supernatant was collected and the volume was adjusted to 40μl with extraction buffer. 4μl of 10X Sample Reduction Agent (Invitrogen, B0009) and 10μl of 4X LDS Sample Buffer (Invitrogen, B0007) were added and samples were heated to 70°C for 10 min.

Lysates amounting to roughly 10 heads per sample were run on a pre-cast 10% Bis-Tris gel and subsequently transferred using the iBlot 2 Gel Transfer Device (Invitrogen). Blots were blocked for an hour in 5% milk in PBS and incubated overnight with primary antibodies in 5% milk at a 1:1000 dilution. They were then washed for 3 × 10 minutes with Tris-buffered saline with 0.1% Tween (TBS-T) and incubated with secondary antibodies in 5% milk for 1 hour at room temperature at a 1:5000 dilution. Blots were washed a further 3 × 10 min with TBS-T before being developed with Pierce ECL Western Blotting Substrate (Thermo Fisher) and imaged using an iBright Imaging System (Thermo Fisher).

#### AAV9 production and delivery

The ORFs of FAF2 and FAF2_PS_ were cloned into the pAAV-CMV-eGFP plasmid (Addgene #105530). AAV9-GFP was obtained from Addgene (105530-AAV9). For FAF2 and FAF2_PS_, endo-free plasmids were prepped with ZymoPURE Midiprep plasmid kit (Zymo Research) and custom AAV9 were generated by Vector BioLabs. AAV9 were assessed for purity by silver stain and titer determined by quantitative PCR. AAV9 were delivered by a single injection to the right lateral ventricle at a dose of 1×10^11^ genome copies per pup at P1 in a PBS solution containing FastGreen dye (Sigma) as previously described.^34^

#### Rotarod assay

Mice were tested on the accelerating rotarod assay as previously described.^50^ Briefly, animals were trained one week prior to first testing at four months of age and subsequent testing was performed monthly. A 60 second warm up was performed before 3 trials which consisted of 5 minutes of acceleration from 4 to 40 rotations per minute with a minimum of 10 minutes between trials. The average of 3 trials was plotted for each animal at each timepoint. Trials were ended when the mouse fell from the rotarod or rounded the testing rod twice without moving.

#### Elevated plus maze assay

Animals were subject to 1 hour of acclimation in testing room while singly housed with 55dB white noise prior to testing. Subsequently, mice were placed on the elevated plus maze with two open and two closed arms for a total of 5 minutes while being monitored with EthoVision XT video tracking software. The percentage of time spent on the closed arms was calculated relative to the total time of testing in assay.

#### Fear conditioning assay

Fear conditioning was performed as previously described.^42,43^ For fear conditioning assay, animals were moved to testing room and singly housed with 55dB white noise for 1 hour prior to testing. For testing, animals were placed in isolation cubicles (30” W × 17.75” D × 18.5”H) (Coulbourn) and monitored with FreezeFrame software (Actimetrics). For training, animals were subject to two pairings of a tone (30 seconds, 85-dB white noise) and foot-shock (2 seconds, 0.5mA) in a 5 minute session. Tone was played from 120–150 seconds and 210–240 seconds followed by foot-shock from 148–150 seconds and 238–240 seconds during session. During training session house lights were turned on and peppermint odor was present. 24 hours after training, mice were once again acclimated for 1 hour to the testing room. On Day 2, animals were subject to both cued and contextual testing randomized for order of assays performed with a 1 hour acclimation between repeat testing. For context testing, animals were returned to the same isolation cubicles under the same conditions for 5 minutes of testing with no tone or foot-shock. For cued testing, animals were placed in randomized isolation cubicles with the house lights off, red light on, infrared light on, white acrylic floor over shock grid, plastic inserts with distinct display patterns over test cage walls and vanilla odor. Animals were subject to a 5 minute test under these conditions with tone but no foot shock. For context testing the average time spent freezing across the 5 minute testing period is reported. For cued testing, an average of the percent time freezing during the two cued stimulus periods (120–150 seconds and 210–240 seconds) during the 5 minute testing period is reported. For the time period prior to cued stimulus used as a measure of baseline freezing, an average of the percent time freezing prior to tone (0–120 seconds) during the cued test is reported.

#### RNA isolation and quantitative reverse-transcriptase PCR

For RNA analysis, total RNA from mouse spinal cord tissue was isolated using TRIzol^^™^^ Reagent (Invitrogen) according to the manufacturer’s instructions followed by treatment with TURBO^^™^^ DNAse (Thermo Fisher Scientific). cDNA was synthesized with RNA to cDNA EcoDry Premix kit (Takara) with random hexamer and oligodT primers. RT-qPCR was done with iQ SYBR Green Supermix (Bio-Rad) with primers listed in Key resources table.

#### Sarkosyl fractionation

Sarkosyl fractionation was performed as previously described.^51^ In brief, brain tissue was homogenized in a 1:10 weight to volume ratio of 10mM Tris-HCl (pH 7.4), 0.8M NaCl and 1mM EDTA buffer supplemented with protease and phosphatase inhibitors (Roche) using a dounce homogenizer. Lysate was spun at 21,000g for 10 minutes at 4C, then the supernatant was incubated with 1% sarkosyl for 1 hour before two 186,000g spins for 1 hour at 4C with a PBS wash between spins. The final pellet was resuspended with PBS.

#### Live cell imaging

For live cell imaging HEK293 tau biosensor cells were seeded in a poly-L-lysine coated 24 well glass bottom plate with #1.5 cover glass at a density of 2.5×10^5^ cells/mL. Halo plasmids (500ng/well) were transfected with Lipofectamine3000 24 hours after plating. 23 hours after transfection, media was exchanged to Fluorobrite DMEM supplemented with 10% FBS and Hoechst 33342 [1μg/mL] (Thermo Fisher) and JF646 Halo ligand [200μM] were added 1 hour prior to imaging. Seeding with CY3-labeled tau seeds was performed immediately prior to imaging and 24 hours post-transfection at a final concentration of 0.05 μM. Images were acquired on a Nikon CSU-W1 SoRa spinning disk confocal microscope with a 20X objective (NA 0.75), using SoRa 2.8X zoom and 10 minute time interval for 18 hours.

#### Mass spectrometry

Brain tissue was lysed in lysis buffer [5% SDS, 1% NaDOC, 0.1M Tris pH 8.5, 10mM TCEP, 40mM 2-Chloroacetamide] supplemented with protease and phosphatase inhibitors at a volume of 10ul lysis buffer per 1 mg of tissue. Tissue was disrupted using a pestle, then passing through a 21G, then 25G syringe. Samples were then boiled for 10 minutes and passed through 27G syringe. Tissue lysates were digested using the SP3 method.^52^ Briefly, 500 μg carboxylate-functionalized speedbeads (Cytiva Life Sciences) were added to tissue lysates. Acetonitrile was added to 80% (v/v) to bind proteins to the beads, then washed twice with 80% (v/v) ethanol and twice with 100% acetonitrile. Proteins were digested with Lys-C/Trypsin (Promega) in 50 mM Tris-HCl buffer, pH 8.5, and incubated at 37˚C overnight. Tryptic peptides were cleaned up using a 1cc (10mg) HLB Oasis cartridges (Waters) according to the manufactures instructions and dried in a speedvac vacuum centrifuge. Peptides were labeled with TMT-Pro reagents (Thermo Scientific) according to the manufacturer’s instructions. Multiplexed samples were immediately cleaned up with HLB Oasis cartridge and dried in a speedvac vacuum centrifuge. To reduce sample complexity, multiplexed peptides were fractionated using a high pH reversed-phase C18 UPLC with a 0.5 mm × 145 mm custom packed Reprosil Saphir rpC18 1.5 μm 100Å (Dr. Maisch Gmbh) column with mobile phases 0.1% (v/v) aqueous ammonia, pH 9.5 in water and acetonitrile (ACN). Peptides were gradient eluted at 20 μL/minute from 2 to 50% ACN in 50 minutes concatenating for a total of 12 fractions using a Waters M-class UPLC (Waters). Peptide fractions were then lyophilized in a speedvac vacuum centrifuge and stored at −20°C until analysis. Fractionated peptides were suspended in 3% (v/v) ACN, 0.1% (v/v) trifluoroacetic acid (TFA) and directly injected onto a reversed-phase C18 1.7 μm, 130 Å, 75 mm × 250 mm M-class column (Waters), using a Vanquish Neo nanoUPLC coupled to an Orbitrap Exploris 480 (Thermo Scientific). Multiplexed peptides were eluted into the mass spectrometers at 300 nL/minute with a gradient from 4% to 20% ACN in 120 minutes then to 40% ACN in 5 minutes. Precursor mass spectra (MS1) were acquired at a resolution of 120,000 from 350 to 1500 m/z with Standard AGC Target and Auto Maximum injection time. Precursor peptide ion isolation width for MS2 fragment scans was 0.7 m/z, and a cycle time of 3 seconds. MS2 spectra were acquired at 30,000 resolution using TurboTMT with HCD at 32% and 200% Normalized AGC Target and Auto maximum injection time was used. Dynamic exclusion was set for 30 seconds. Rawfiles were searched against the Mus musculus database (UP000000589) using MaxQuant v.2.6.3.0. Cysteine carbamidomethylation was considered a fixed modification, while methionine oxidation and protein N-terminal acetylation were searched as variable modifications. All peptide and protein identifications were thresholded at a 1% false discovery rate (FDR). TMT reporter ion intensities were Cyclic loess normalized and log2 fold changes and p-values were calculated with limma (Bioconductor.com) using an R-script.

### QUANTIFICATION AND STATISTICAL ANALYSIS

#### Confocal microscopy and image quantification

For fluorescence imaging in cells, images were acquired on a Nikon CSU-W1 SoRa spinning disk confocal microscope with a 100X (NA 1.45) objective. To quantify the fold enrichment of proteins in tau aggregates, a CellProfiler pipeline was generated to segment the cytoplasm and nuclei of individual cells. Next, cells that were positively transfected with Halo constructs were filtered based on intensity measurements. From this pool of Halo+ cells, cytoplasmic tau aggregates were then identified and segmented. Mean intensity measurements were taken and the fold enrichment reported for each cell as the mean intensity of Halo signal within tau aggregates / mean intensity of the remainder of the cytoplasm.

For immunofluorescence in tissues, images of the hippocampus were acquired on a Nikon spinning disk confocal microscope with a 10X (NA 0.45) objective in a 3x3 grid with 3μm z-steps using stitching and overlap to obtain composite images. For Iba1 quantification maximum projections of sections were manually annotated to mask for the hippocampus based on NeuN signal, then processed with CellProfiler to identify microglia based on Iba1 staining, respectively. The percent area coverage of each cell type was determined by dividing the total Iba1 positive area by the area of the hippocampus for each section. For phosphorylated tau quantification maximum projections of sections were manually annotated to mask for the hippocampus based on NeuN signal, then processed with CellProfiler to set a threshold for phosphorylated tau positivity. The positive area was then divided by the area of the hippocampus for each section to determine the percent area coverage. Images of the cerebellum were acquired with a 10X (NA 0.45) objective in a 4x4 grid. Regions of interest were manually selected and area quantified with NIS-Elements AR (Nikon) software.

#### Data representation and statistical analysis

For bar graphs, data represents mean, SEM and individual data points of replicates. For boxplots data represents median, mean (+), interquartile range, minimum and maximum unless otherwise specified. Statistical tests and replicates are reported in figure legends. Line graphs represent mean and SEM for each timepoint. In figures, statistical significance is reported as follows (*) p<0.05, (**) p<0.01, (***) p<0.001, (****) p<0.0001 for the defined p-values. Statistical analysis was performed with GraphPad Prism 10 (v10.2.2).

## Supplementary Material

1

2

3 **Data S1.** High resolution Western blot figures, related to Figures 1–7

4 **Table S1.** Proteomics of brain extracts from AAV-injected animals, related to Figure 7

5 **Table S2.** Primers for RT-qPCR analysis, related to Figure 5

6 **Video S1.** Tau aggregation with Halo expression, related to Figure 4

7 **Video S2.** Tau aggregation with polySer expression, related to Figure 4

8 **Video S3.** Tau aggregation with targeted FAF2 expression, related to Figure 4

9 **Video S4.** Tau aggregation with FAF2 expression, related to Figure 4

**Document S1.** Figures S1–S13

## Figures and Tables

**Figure 8.**
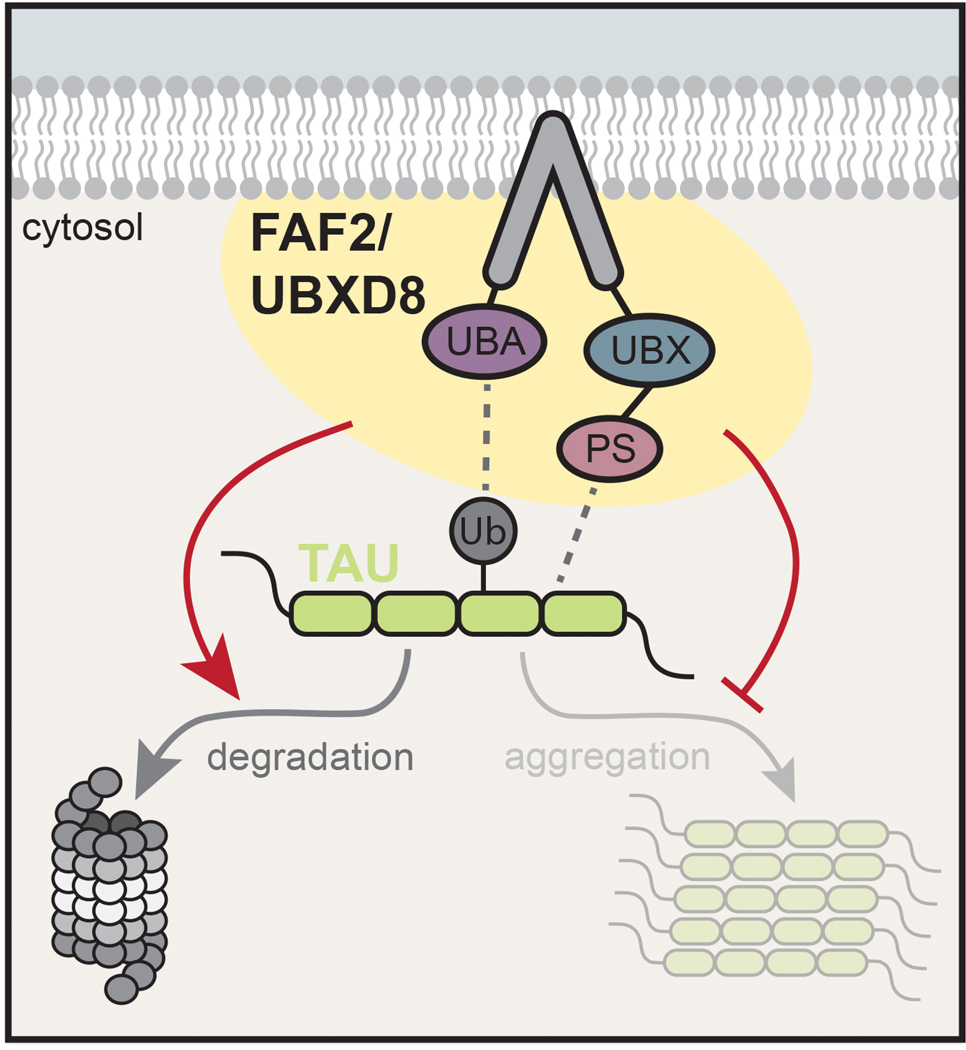
Model of polyserine-targeted FAF2 effects on tau levels and aggregation. Addition of a polySer motif promotes association of FAF2 with tau where it can prevent aggregation and promote turnover via the proteasome through functions of the UBA, membrane, and UBX domains.

**Table:** Key resources table

REAGENT or RESOURCE	SOURCE	IDENTIFIER
**Antibodies**		
Rabbit anti-Halo	Promega	Cat#G9281; RRID:AB_713650
Rabbit anti-GFP	Cell Signaling	Cat#2555S; RRID:AB_10692764
GAPDH-HRP	Santa Cruz	Cat#sc-47724 HRP; RRID:AB_627678
Rabbit anti-VCP	Cell Signaling Technology	Cat#2648; RRID:AB_2214632
Mouse anti-Ubiquitin	Cell Signaling Technology	Cat#3936S; RRID:AB_331292
Rabbit anti-p62	MBL International	Cat#PM045; RRID:AB_1279301
Rabbit anti-LC3B	Novus Biologicals	Cat#NB600–1384; RRID:AB_669581
Chicken anti-GFP	Aves	Cat#GFP-1020; RRID:AB_10000240
Guinea pig anti-NeuN	Synaptic Systems	Cat#266 004; RRID:AB_2619988
Mouse anti-GAPDH	Millipore Sigma	Cat#MAB374; RRID:AB_2107445
Rabbit anti-tau (K18)	Abcam	Cat#ab218314; RRID:AB_3068614
Mouse anti-p-tau (AT8)	Thermo Fisher Scientific	Cat#MN1020; RRID:AB_223647
Mouse anti-p-tau (AT180)	Thermo Fisher Scientific	Cat#MN1040; RRID:AB_223649
Rabbit anti-p-tau (AH36)	StressMarq Biosciences	Cat#SMC-601; RRID:AB_2820300
Mouse anti-Pcp2	Santa Cruz	Cat#sc-137064; RRID:AB_2158439
Rabbit anti-Iba1	Wako	Cat#019–19741; RRID:AB_839504
Anti-rabbit HRP	Cell Signaling	Cat#7074S; AB_2099233
Anti-mouse HRP	Cell Signaling	Cat#7076S; RRID:AB_330924
488 anti-chicken	Jackson ImmunoResearch Labs	Cat#703–545-155; RRID:AB_2340375
CY3 anti-rabbit	Jackson ImmunoResearch Labs	Cat#711–165-152; AB_2307443
CY3 anti-mouse	Jackson ImmunoResearch Labs	Cat#715–165-150; RRID:AB_2340813
CY5 anti-guinea pig	Jackson ImmunoResearch Labs	Cat#706–175-148; RRID:AB_2340462
**Bacterial and virus strains**		
Rosetta 2(DE3)pLysS Competent Cells	Sigma-Aldrich	Cat#71403
AAV9-eGFP	pAAV.CMV.PI.EGFP. WPRE.bGH was a gift from James M. Wilson (Addgene viral prep # 105530-AAV9)	Cat#105530-AAV9
AAV9-FAF2-eGFP	Vector BioLabs; This paper	Custom AAV
AAV9-FAF2_PS_-eGFP	Vector BioLabs; This paper	Custom AAV
**Biological samples**		
Clarified brain homogenate from Tg(Thy1-MAPT*P301S)2541 tau transgenic mice	Lester et al.^15^	N/A
**Chemicals, peptides, and recombinant proteins**		
DAPI	Invitrogen	Cat#62248
Hoechst 33342	Thermo Fisher Scientific	Cat#62249
TAK-243	MedChem Express	Cat#HY-100487
DBeQ	Thermo Fisher Scientific	Cat#44–171-0
CB-5083	MedChem Express	Cat#HY-12861
NMS-873	MedChem Express	Cat#HY-15713
VER-155008	Tocris	Cat#3803
MAL3–101	MedChem Express	Cat#HY-124805
MG132	Sigma-Aldrich	Cat#M7449
BafA1	Cell Signaling	Cat#54645
TMRDirect	Promega	Cat#G2991
Janelia Fluor 503	Promega	Cat#HT1010
Janelia Fluor 646	Promega	Cat#GA1121
cOmplete Protease inhibitor cocktail, EDTA-free	Roche	Cat#11836170001
Phosstop Phosphatase inhibitor tablets	Roche	Cat#4906837001
Lipofectamine3000	Invitrogen	Cat#L3000001
Lipofectamine RNAiMAX	Invitrogen	Cat#13778075
ProLong Glass Antifade Mountant	Thermo Fisher Scientific	Cat#P36982
Methanol-free Formaldehyde	Thermo Fisher Scientific	Cat#28908
FluoromountG	Electron Microscopy Sciences	Cat#17984–25
FastGreen	Sigma-Aldrich	Cat#F7258
TRIzol Reagent	Invitrogen	Cat#15596018
TURBO DNAse	Thermo Fisher Scientific	Cat#AM2238
Donkey serum	EMD Millipore	Cat#S30-M
**Critical commercial assays**		
In-Fusion Snap Assembly Master Mix	Takara	Cat#638947
QuikChange II Site-directed mutagenesis kit	Agilent Technologies	Cat#200521
Halo-Trap Agarose beads	ChromoTek	Cat#ota-20
Clarity Western ECL Substrate	Bio-Rad	Cat#1705061
Pierce ECL Western Blotting Substrate	Thermo Fisher Scientific	Cat#32106
ZymoPURE Midiprep plasmid kit	Zymo Research	Cat#D4201
RNA to cDNA EcoDry Premix kit	Takara	Cat#639547
iQ SYBR Green Supermix	Bio-Rad	Cat#1708880
**Deposited data**		
Raw proteomics data	This paper	ProteomeXchange Consortium via the PRIDE partner repository; Dataset identifier: 10.6019/PXD066746
**Experimental models: Cell lines**		
Tau RD P301S FRET Biosensor	ATCC	Cat#CRL-3275; RRID:CVCL_DA04
HEK293T	ATCC	Cat#CRL-3216; RRID:CVCL_0063
**Experimental models: Organisms/strains**		
*D. melanogaster*: GMR-GAL4	Bloomington Drosophila Stock Center	RRID:BDSC_1104
*D. melanogaster*: UAS human Tau 2N4R Line	Gift from Guy Tear (King’s College London, UK); Povellato et al.^30^	N/A
Mouse: C57BL/6J (WT)	The Jackson Laboratory	Cat#000664; RRID:IMSR_JAX:000664
Mouse: B6.Cg-Tg(Prnp-MAPT*P301S)PS19Vle/J (PS19)	The Jackson Laboratory	Cat#024841; RRID:IMSR_JAX:024841
**Oligonucleotides**		
PS19 Genotyping Primers Common Forward (5’-3’): TTGAAGTTGGGTTATCAATTTGG	Integrated DNA Technologies	Related to RRID:IMSR_JAX:024841
PS19 Genotyping Primers WT Reverse (5’-3’): TTCTTGGAACACAAACCATTTC	Integrated DNA Technologies	Related to RRID:IMSR_JAX:024841
PS19 Genotyping Primers Mut Reverse (5’-3’): AAATTCCTCAGCAACTGTGGT	Integrated DNA Technologies	Related to RRID:IMSR_JAX:024841
Negative control siRNA	Thermo Fisher Scientific	Cat#4390843
VCP siRNA (1)	Thermo Fisher Scientific	Cat#s14765
VCP siRNA (2)	Thermo Fisher Scientific	Cat#s14767
**Recombinant DNA**		
pcDNA3.1 Mammalian Expression Plasmid	Invitrogen	Cat#V79020
pET29b Tau Cys Bacterial Expression Plasmid	pET29b Tau Cys was a gift from Gabriele Kaminski Schierle (Addgene plasmid # 108867)	Cat#108867; RRID:Addgene_108 867
pJFRC-MUH Plasmid	Addgene; Pfeiffer et al.^47^	Cat#26213; RRID:Addgene_26213
pAAV-CMV-PI-eGFP-WPRE-bGH Plasmid	pAAV.CMV.PI.EGFP. WPRE.bGH was a gift from James M. Wilson (Addgene plasmid # 105530)	Cat#105530; RRID:Addgene_105530
**Software and algorithms**		
NIS-Elements	Nikon	RRID:SCR_014329
CellProfiler Image Analysis Software Version 4.2.1	CellProfiler	RRID:SCR_007358
Prism 10	GraphPad	https://www.graphpad.com/features
FreezeFrame 5	Actimetrics	https://actimetrics.com/products/freezeframe/
**Other**		
BD FACSCelestaTM Cell Analyzer	BD; University of Colorado Boulder Flow Cytometry Shared Core Facility	RRID:SCR_019309
4–12% Tris-Glycine gels	Thermo Fisher Scientific	Cat#XP04120BOX
4–20% Tris-Glycine gels	Thermo Fisher Scientific	Cat#XP04205BOX
iBlot 2 Transfer Device	Thermo Fisher Scientific	Cat#IB21001
iBright Imaging System	Invitrogen	Model CL1500
M205 FCA stereomicroscope	Leica	https://www.leica-microsystems.com/products/light-microscopes/stereomicroscopes/p/leica-m205-fca/
CSU-W1 SoRa spinning disk confocal microscope	Nikon	https://www.microscope.healthcare.nikon.com/products/confocal-microscopes/csu-series/csu-w1-sora

## Highlights:

- Polyserine can be broadly utilized to target fusion proteins to tau aggregates
- FAF2/UBXD8 prevents tau aggregation via UBA, hairpin and UBX domains
- Polyserine-targeted FAF2/UBXD8 selectively reduces tau levels through the proteasome
- FAF2/UBXD8 suppression of tau pathology in animal models is enhanced by polyserine
