# Supplementary figures and images for: Polyserine-mediated targeting of FAF2/UBXD8 ameliorates tau aggregation
Source: Neuron. Author manuscript; Available in PMC 2025 Sep 11. (PMC12422715; doi:10.1016/j.neuron.2025.08.002)

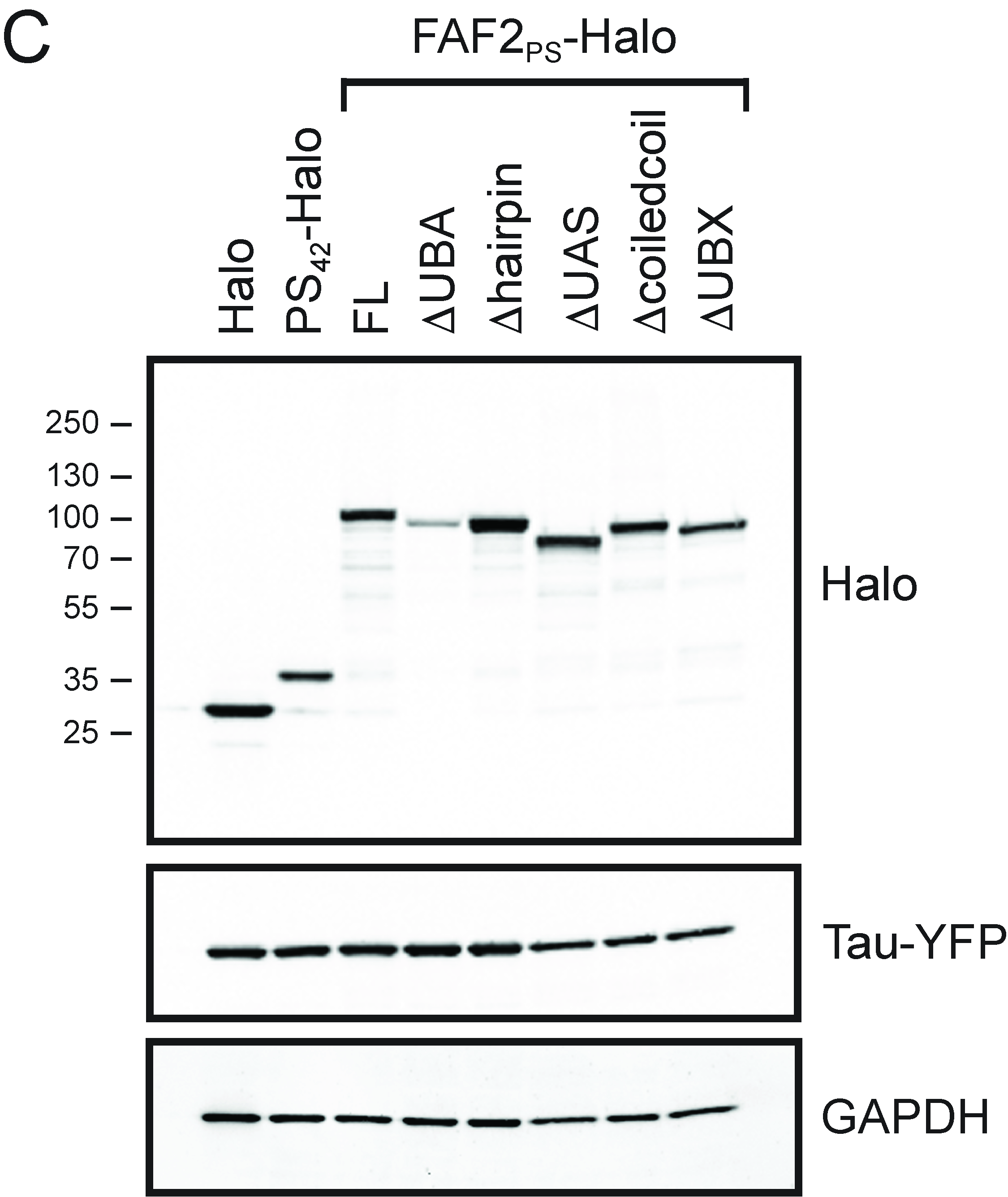

Supplement: 1 [file NIHMS2103916-supplement-1.zip › Data S1 High Resolution Western Blot Figures/Figure S5C.tif]

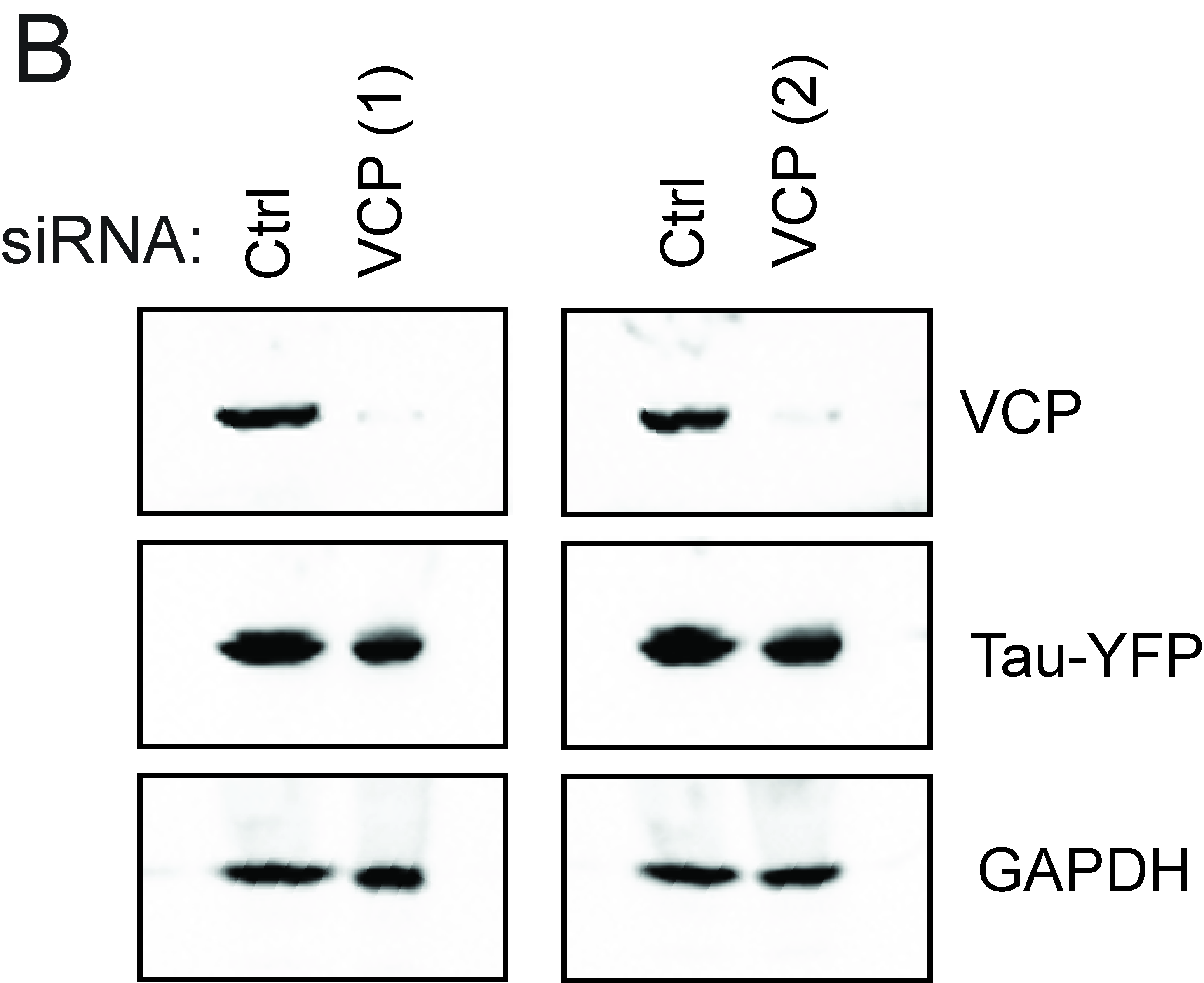

Supplement: 1 [file NIHMS2103916-supplement-1.zip › Data S1 High Resolution Western Blot Figures/Figure S6B.tif]

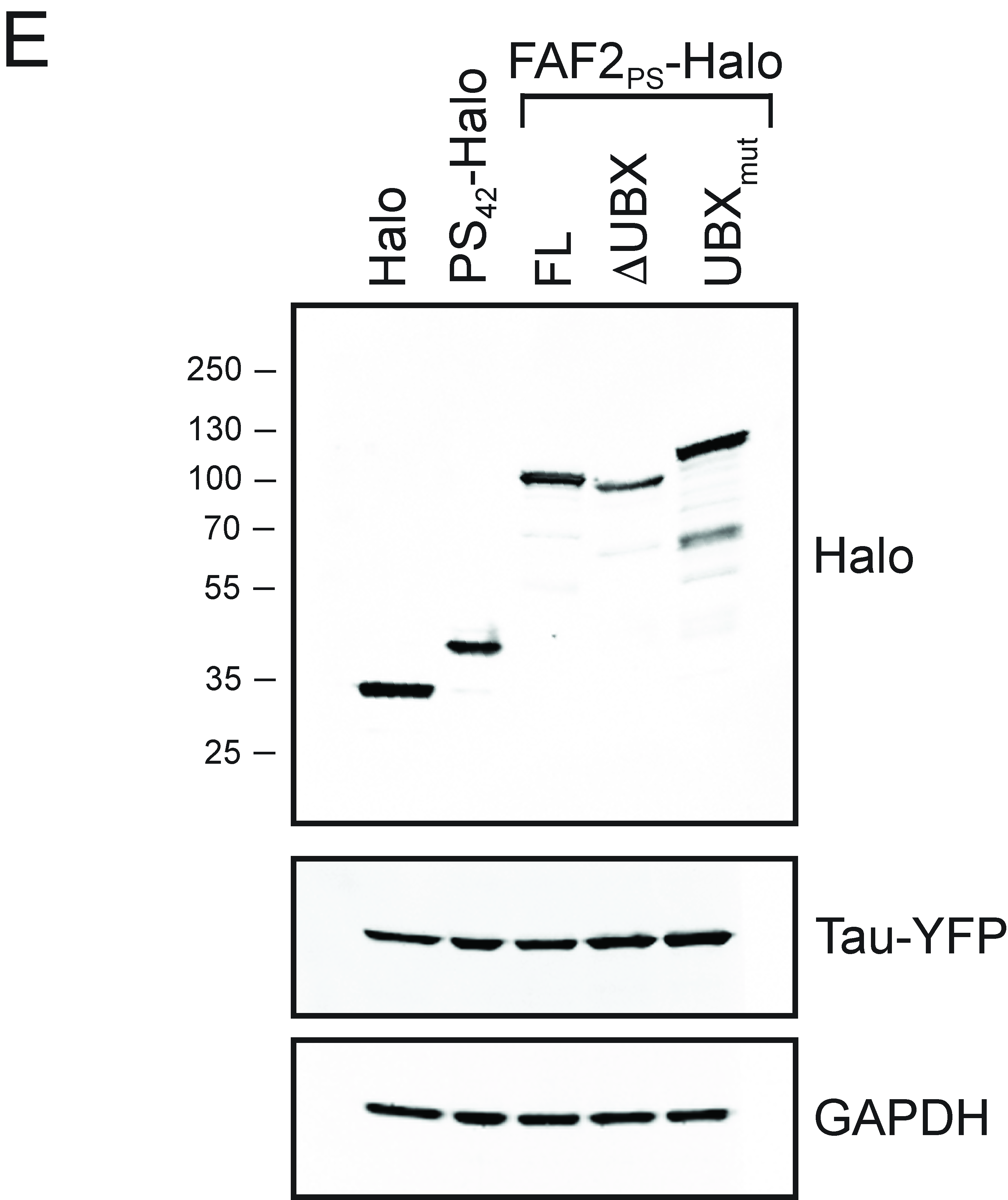

Supplement: 1 [file NIHMS2103916-supplement-1.zip › Data S1 High Resolution Western Blot Figures/Figure S6E.tif]

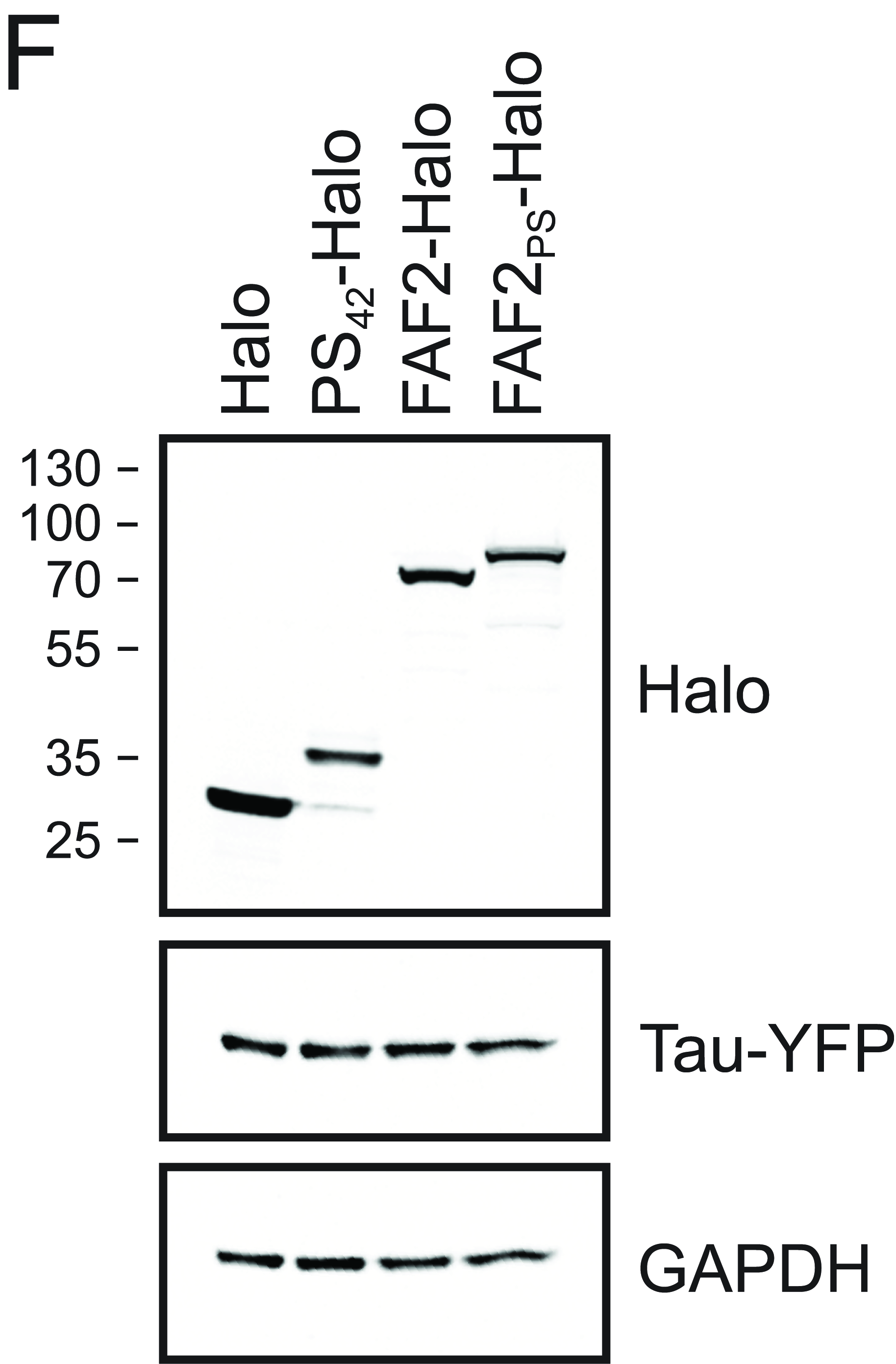

Supplement: 1 [file NIHMS2103916-supplement-1.zip › Data S1 High Resolution Western Blot Figures/Figure S3.tif]

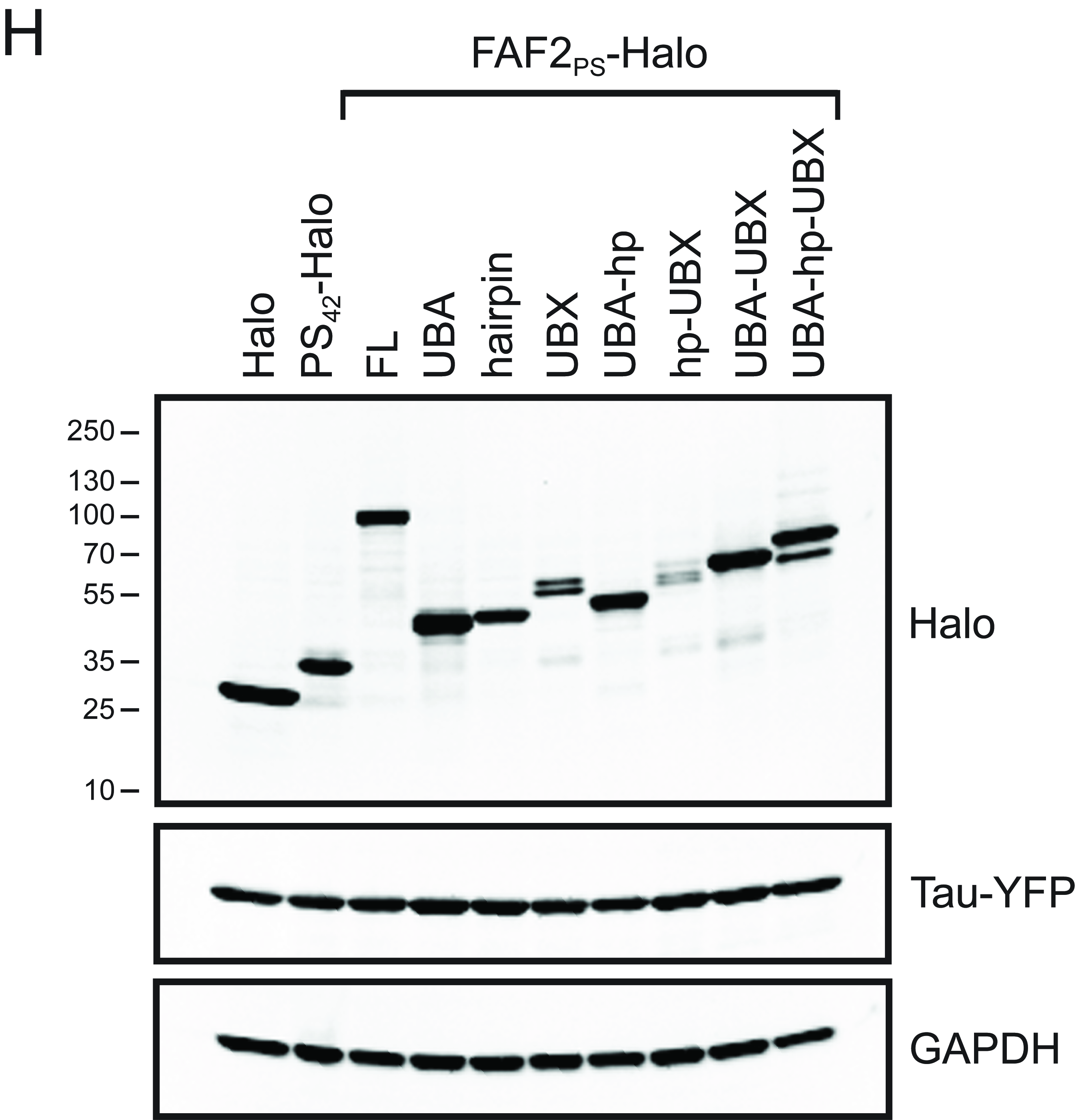

Supplement: 1 [file NIHMS2103916-supplement-1.zip › Data S1 High Resolution Western Blot Figures/Figure S5H.tif]

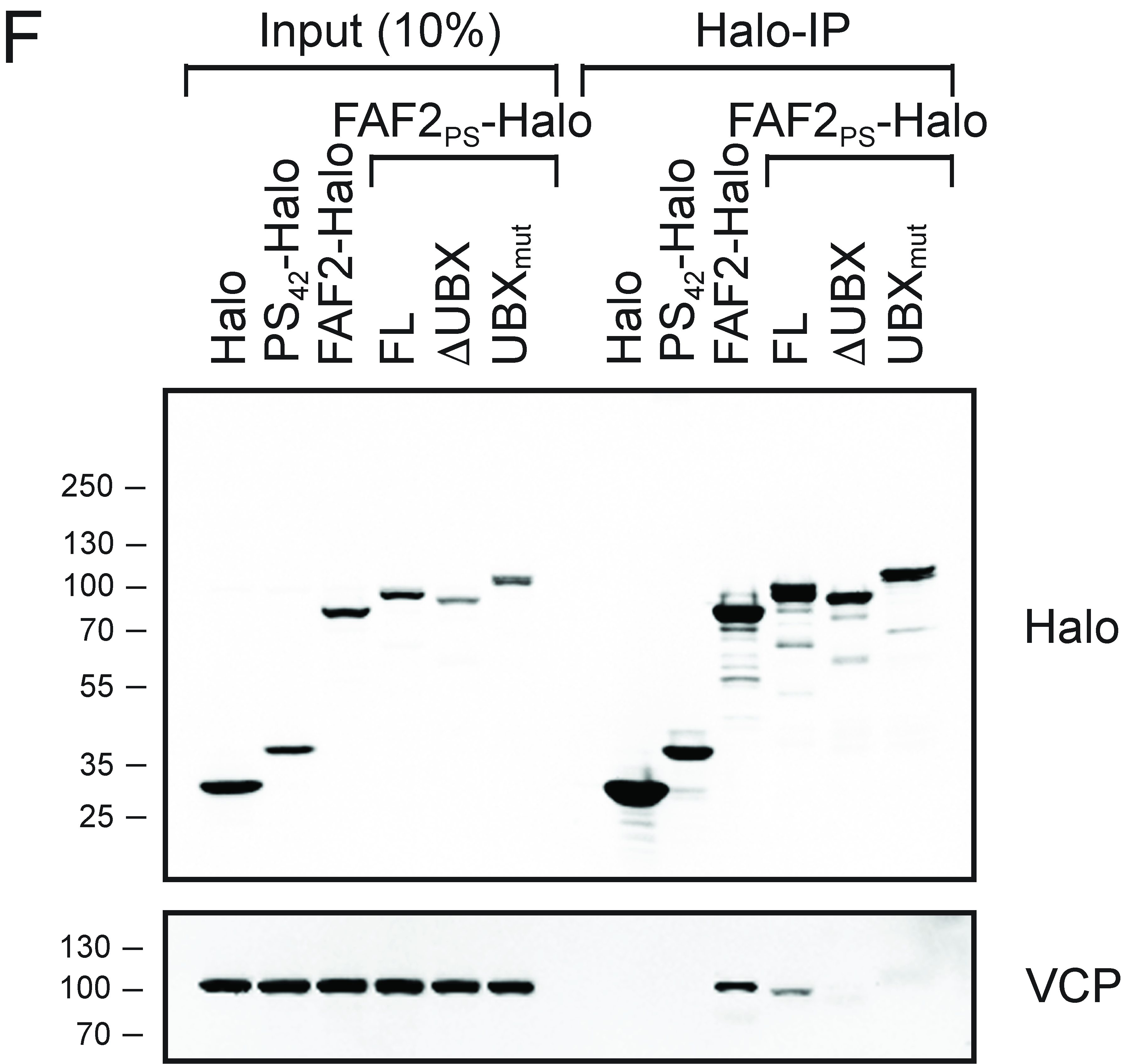

Supplement: 1 [file NIHMS2103916-supplement-1.zip › Data S1 High Resolution Western Blot Figures/Figure S6F.tif]

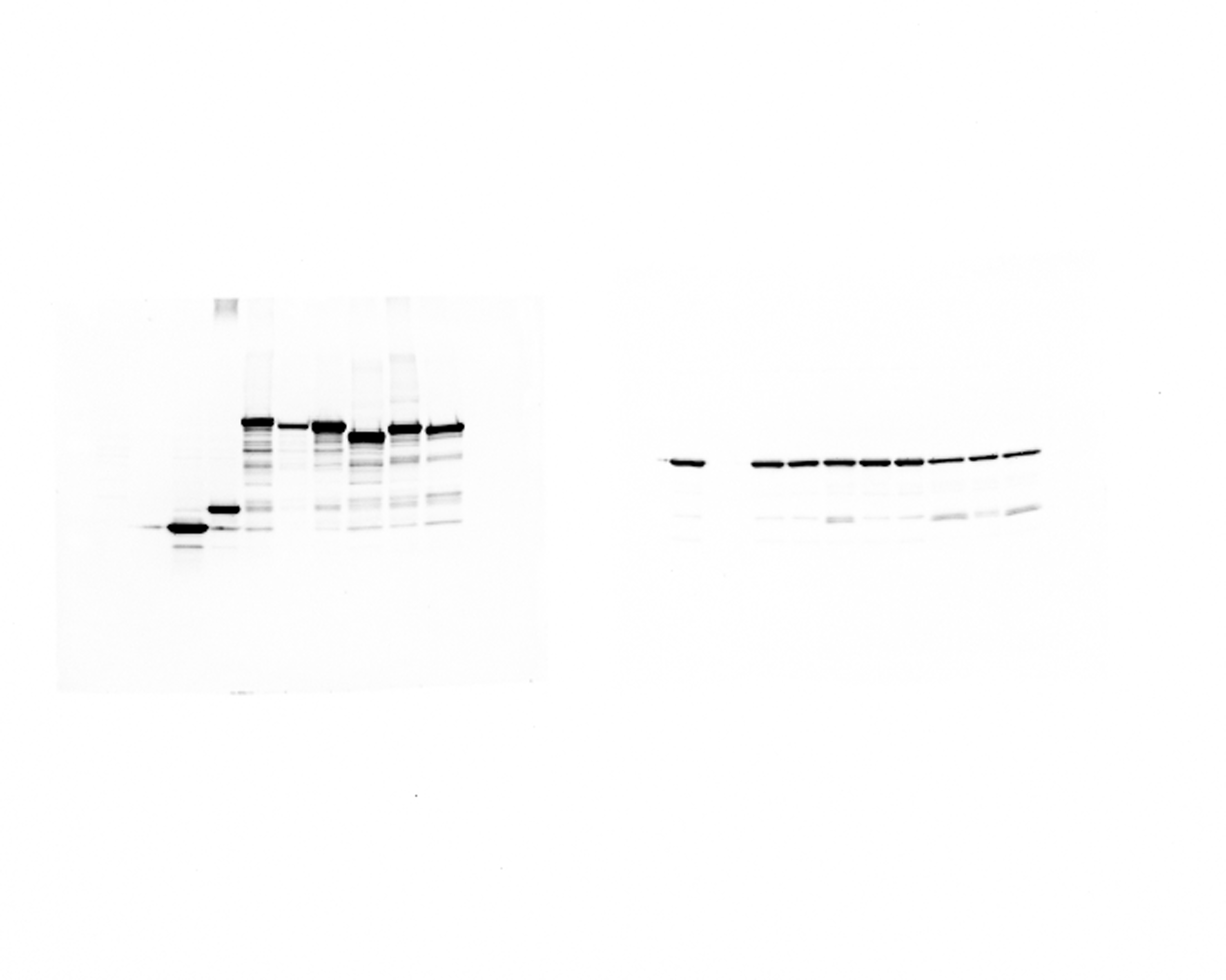

Supplement: 2 [file NIHMS2103916-supplement-2.zip › Figure S5C Raw WB Images/Figure S5C Tau-YFP.tif]

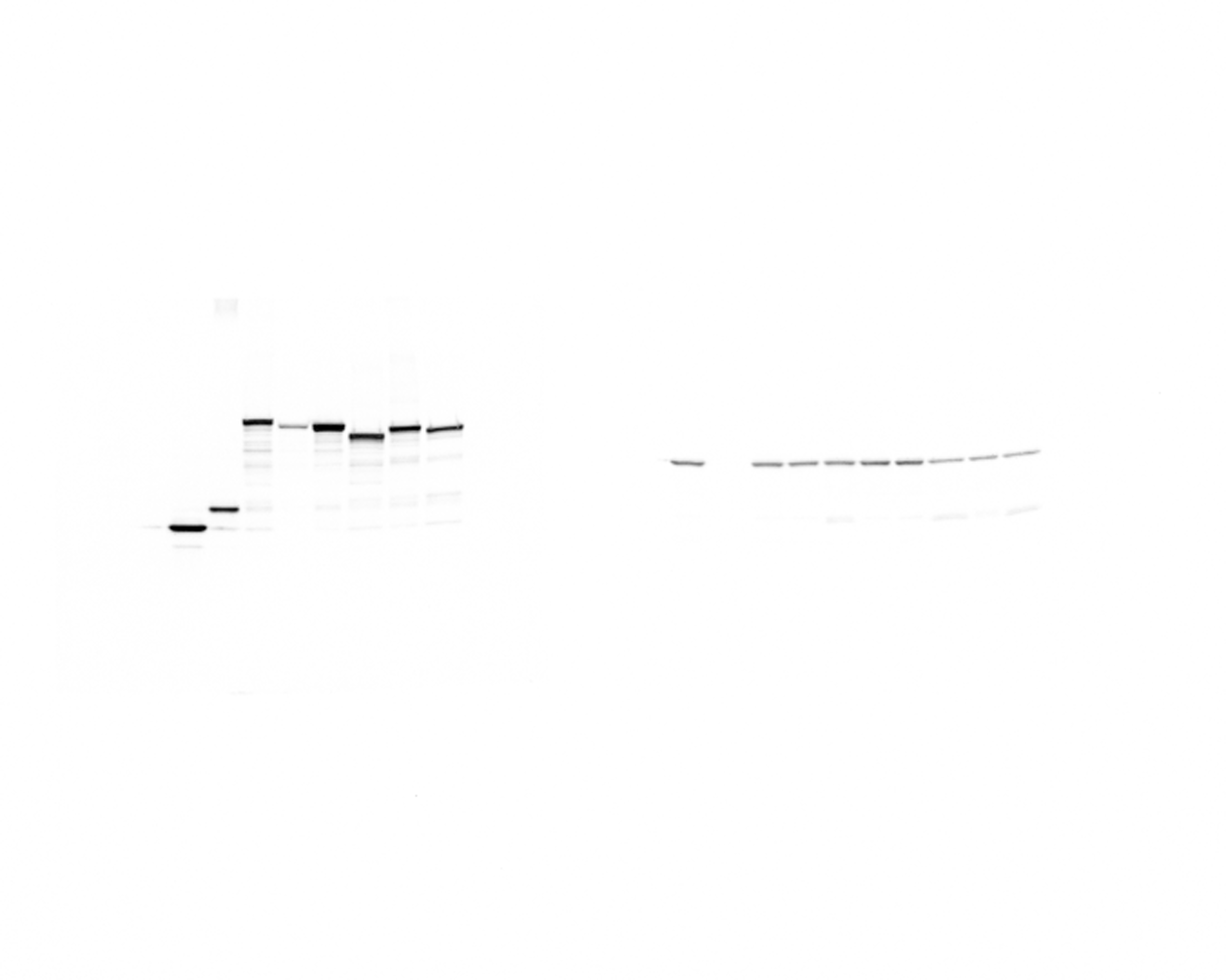

Supplement: 2 [file NIHMS2103916-supplement-2.zip › Figure S5C Raw WB Images/Figure S5C Halo.tif]

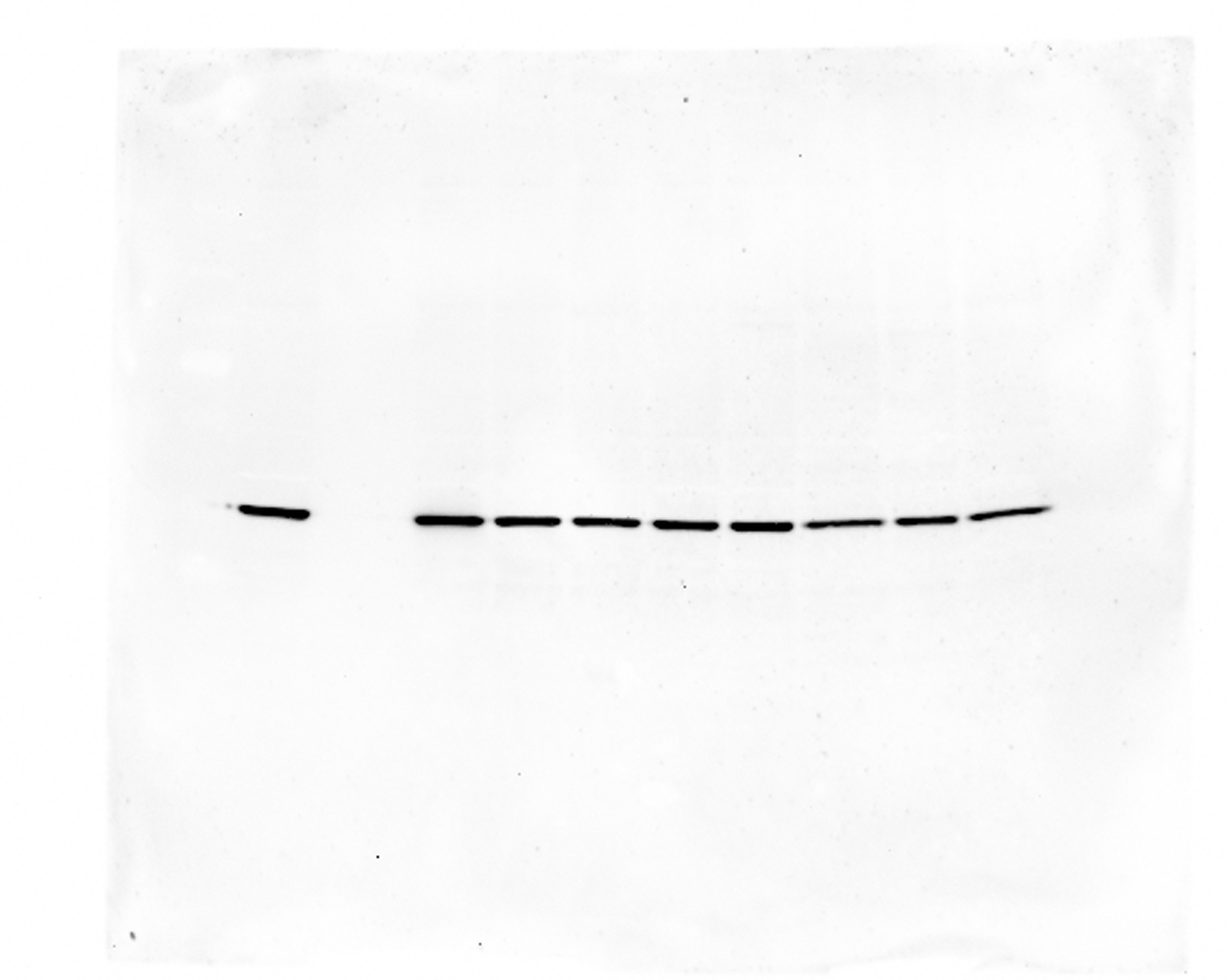

Supplement: 2 [file NIHMS2103916-supplement-2.zip › Figure S5C Raw WB Images/Figure S5C GAPDH.tif]
