# Supplementary material for: Polyserine-mediated targeting of FAF2/UBXD8 ameliorates tau aggregation
Source: Neuron. Author manuscript; Available in PMC 2025 Sep 11. (PMC12422715; doi:10.1016/j.neuron.2025.08.002)
Supplement: 3 — Data S1. High resolution Western blot figures, related to Figures 1–7 [file NIHMS2103916-supplement-3.pdf]

Supplemental Information

This PDF file includes:

Figures S1-S12

A

SRRM2-Ct Targeting Sequence (PS_{SRRM2}):

QSRCLIAQTTPVAGSQSLSSGAVATTTSSAGDHNGMLSVPAPGVPHSDVGEPPASTGAQQP-
SALAALQPAKERRSSSSSSSSSSSSSSSSSSSSSSSSSGSSSSDSEGSSLPVQPEVALKRVP
SPTPAPKEAVREGRPPEPTPAKRKRRSSSSSSSSSSSSSSSSSSSSSSSSSSSSSSSSSSSS
SSSSSSPSPAKPGPQALPKPASPKKPPPGERRSRSPRKPIDSLRDSRSLSYSPVERRRPSPQ
PSPRDQQSSSSERGSRRGQRGDSRSPSHKRRRETPSPRPMRHRS

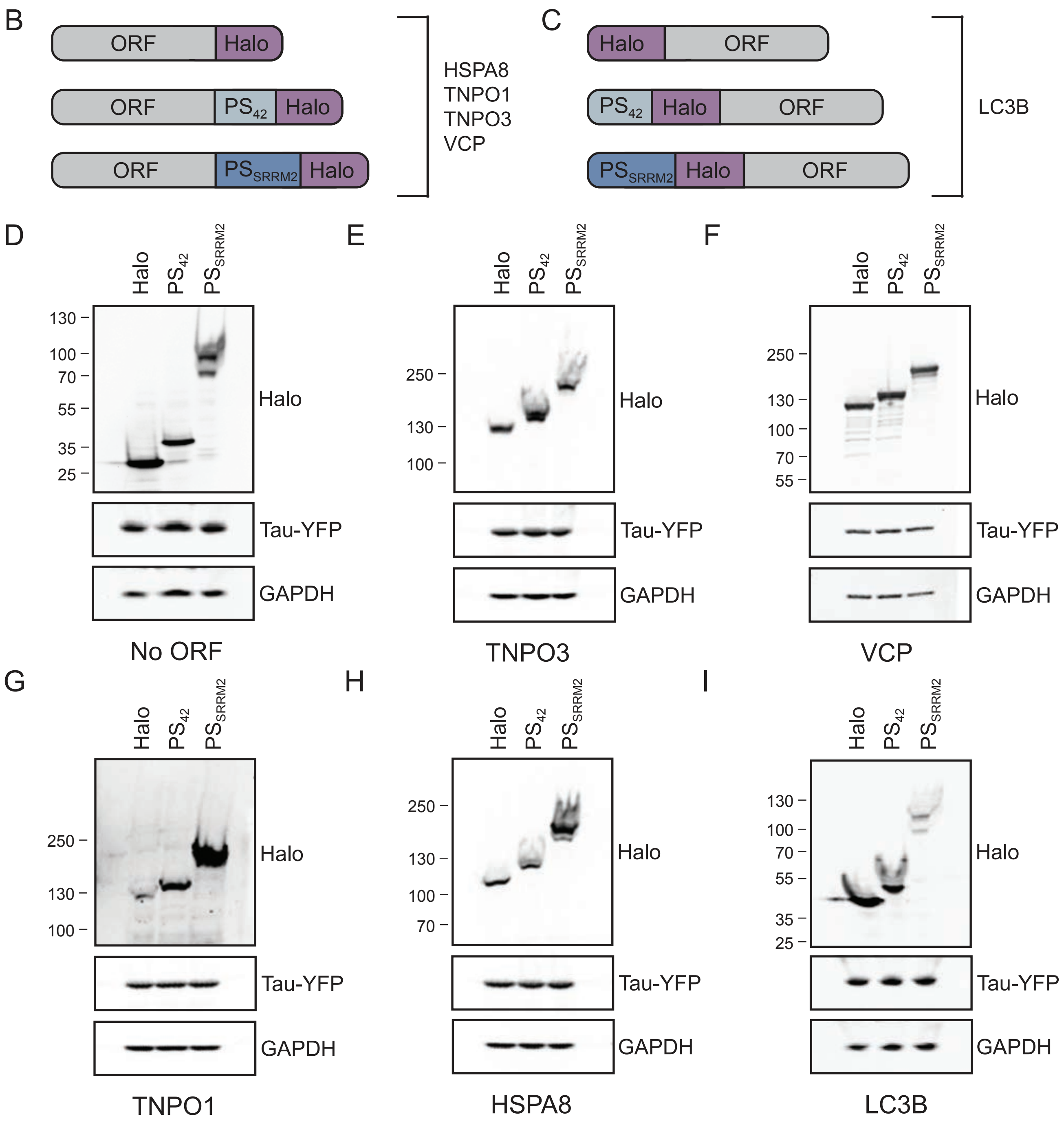

Figure S1

Figure S1. Polyserine fusion proteins do not modulate tau levels, related to Figure 1. (**A**) Amino acid sequence of the SRRM2-Ct targeting region (PS_{SRRM2}) which contains two polySer stretches (blue). (**B**) Schematic of constructs with C-terminal polySer targeting motifs and Halo tags. (**C**) Schematic of constructs with N-terminal polySer targeting motifs and Halo tags. (**D-I**) Western blot for Halo, tau-YFP and GAPDH as a loading control of extracts from HEK293T tau biosensor cells transfected with constructs expressing Halo, 42-polySer-Halo (PS_{42}) and SRRM2-Ct (PS_{SRRM2}) fused proteins with either no additional ORF (D) or TNPO3 (E), VCP (F), TNPO1 (G), HSPA8 (H), and LC3B (I) as depicted in (B) and (C).

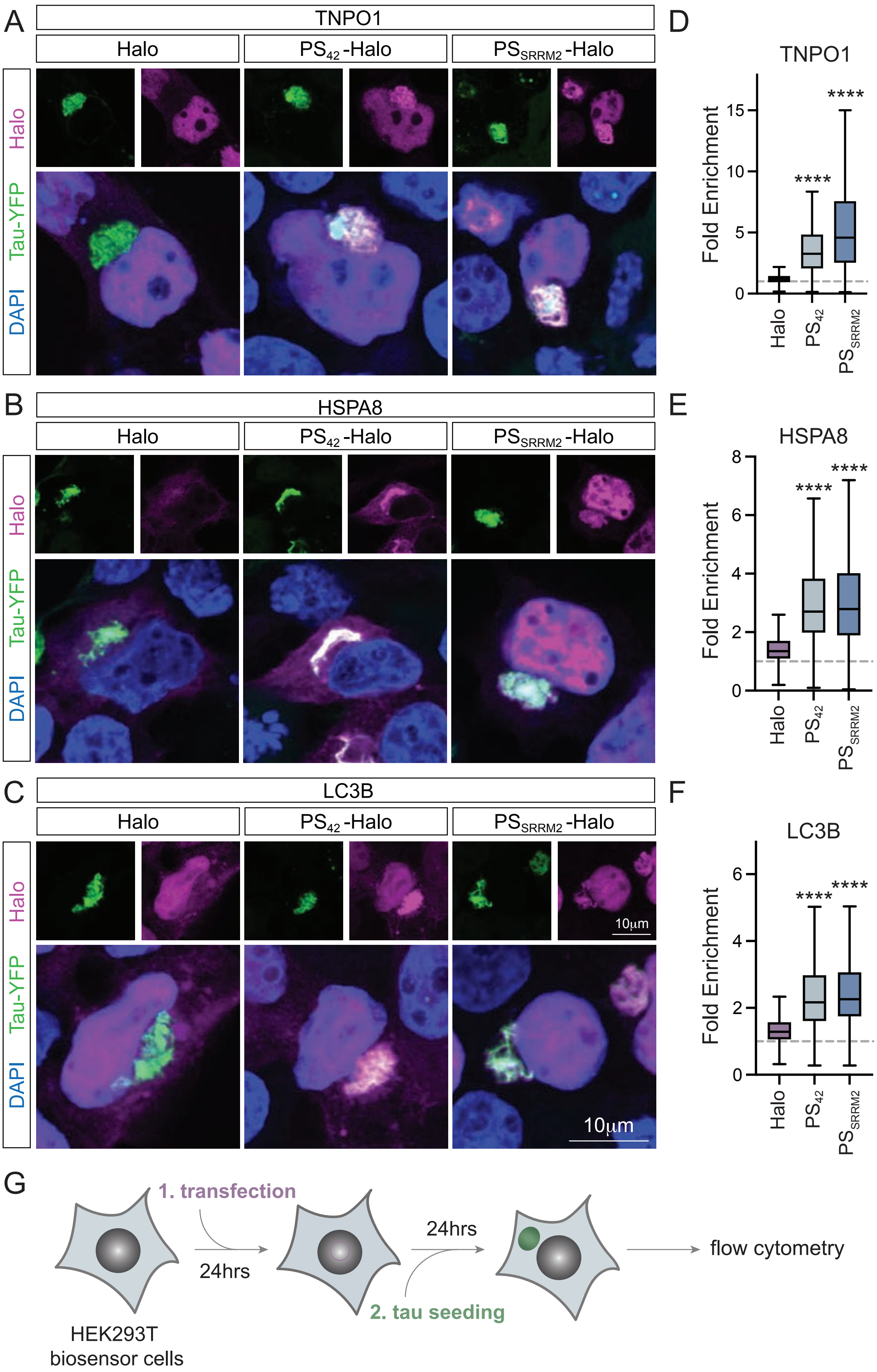
A
TNPO1
Halo
PS42-Halo
PSSRRM2-Halo
Halo
Tau-YFP
DAPI
D
TNPO1
Fold Enrichment
****
****
B
HSPA8
E
C
LC3B
F
10µm
G
1. transfection
24hrs
24hrs
2. tau seeding
flow cytometry
HEK293T
biosensor cells

Figure S2

Figure S2. Polyserine targeting increases enrichment of fusion proteins in tau aggregates, related to Figure 1. (**A-C**) Fluorescence imaging of DAPI (blue), tau-YFP (green) and Halo (magenta) in HEK293T tau biosensor cells transfected with Halo, 42-polySer-Halo (PS_{42}) and SRRM2-Ct (PS_{SRRM2}) constructs fused to TNPO1 (A), HSPA8 (B), or LC3B (C) (**D-F**) Quantification of the fold enrichment of Halo signal in cytoplasmic tau aggregates relative to the remainder of the cytoplasm in cells as in (A-C). Boxplot represents median, interquartile range, and whiskers determined by Tukey method. Data represents fold enrichment determined for (TNPO1: Halo n=559, PS_{42} n=294, PS_{SRRM2} n=363; HSPA8: Halo n=619, PS_{42} n=510, PS_{SRRM2} n=510; LC3B: Halo n=328, PS_{42} n=1428, PS_{SRRM2} n=1243) individual cells quantified from n=3 biological replicates. Statistics performed with one-way ANOVA and Dunnett's multiple comparisons test. (**G**) Schematic of flow cytometry experiments with plasmid transfection and tau seeding for Figure 1G and Figure 2A, D, E, G, and I.

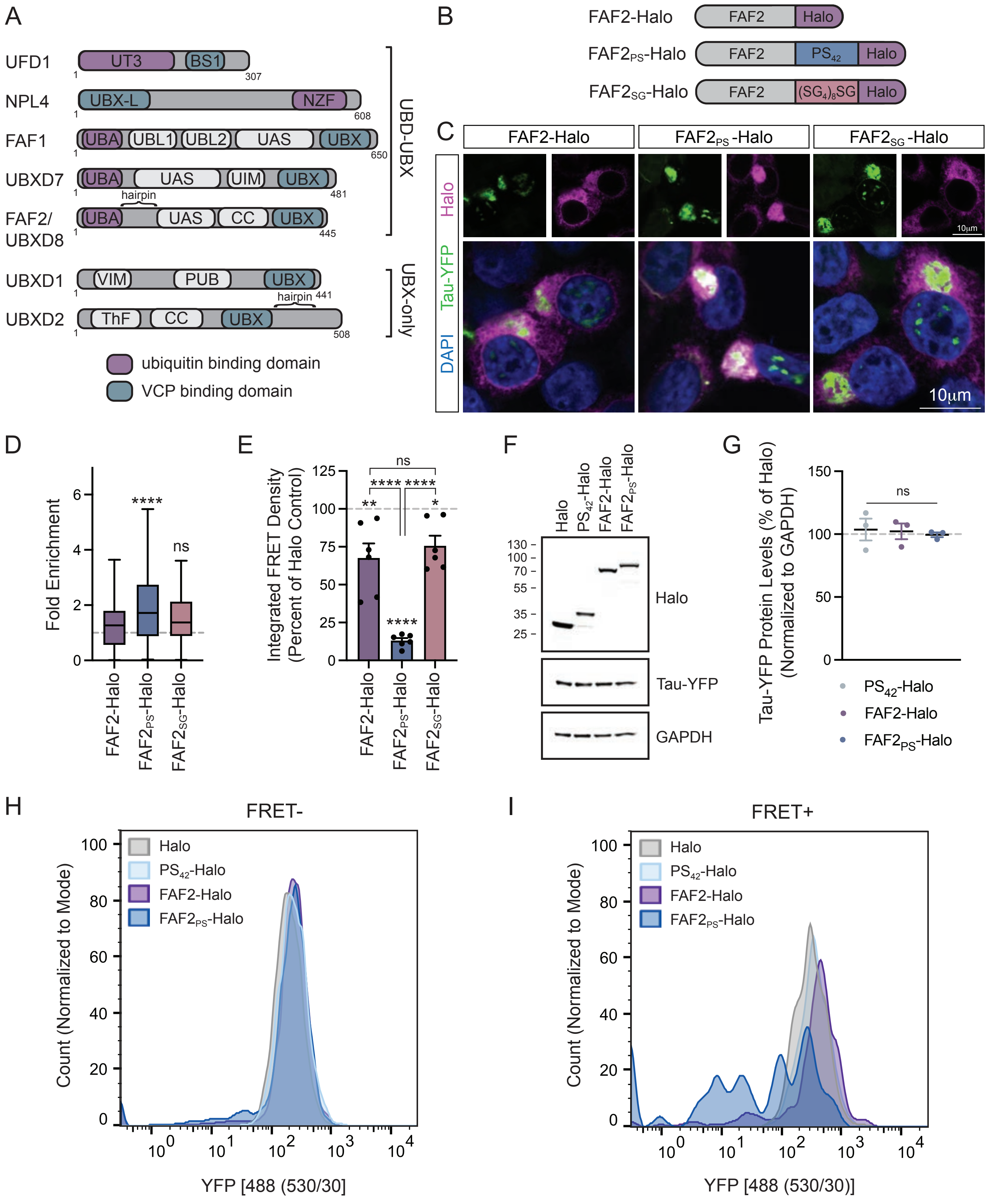
A
UFD1
UT3
BS1
NPL4
UBX-L
NZF
FAF1
UBA
UBL1
UBL2
UAS
UBX
UBXD7
UIM
hairpin
FAF2/
UBXD8
CC
UBD-UBX
UBXD1
VIM
PUB
UBXD2
ThF
UBX-only
ubiquitin binding domain
VCP binding domain
B
FAF2-Halo
FAF2PS-Halo
FAF2SG-Halo
C
Halo
Tau-YFP
DAPI
10µm
D
Fold Enrichment
****
ns
E
Integrated FRET Density
(Percent of Halo Control)
F
PS42-Halo
Tau-YFP
GAPDH
G
Tau-YFP Protein Levels (% of Halo)
(Normalized to GAPDH)
H
FRET-
Count (Normalized to Mode)
YFP [488 (530/30]
I
FRET+
YFP [488 (530/30)]

Figure S3

Figure S3. Polyserine targeting of FAF2 selectively reduces tau in cells with aggregates, related to Figure 2. (**A**) Schematic of VCP adaptor proteins tested for effects on tau aggregation. (**B**) Schematic of Halo-tagged FAF2 constructs with or without 42-polySer ($FAF2_{PS}$) or an equivalent length serine-glycine linker made up of a repeating 'SGGGG' sequence for 42 total amino acids ($FAF2_{SG}$). (**C**) Fluorescence imaging of DAPI (blue), tau-YFP (green) and Halo (magenta) in HEK293T tau biosensor cells transfected with FAF2-Halo, $FAF2_{PS}$-Halo and $FAF2_{SG}$-Halo. (**D**) Quantification of the fold enrichment of Halo signal in cytoplasmic tau aggregates relative to the remainder of the cytoplasm in cells as in (C). Boxplot represents median, interquartile range, and whiskers determined by Tukey method. Data represents fold enrichment determined for individual cells (FAF2-Halo n=290; $FAF2_{PS}$-Halo n=203, $FAF2_{SG}$-Halo n=306) quantified from 3 biological replicates. Statistics performed with one-way ANOVA and Tukey's multiple comparisons test. (**E**) Integrated FRET density of the top 10% of HEK293T tau biosensor cells pre-transfected with Halo, FAF2-Halo, $FAF2_{PS}$-Halo and $FAF2_{SG}$-Halo seeded with clarified tau brain homogenate and normalized to Halo. Data represent mean, SEM and individual values of each replicate. Statistics performed with one-way ANOVA and Tukey's multiple comparisons test. n=6. (**F**) Representative Western blot for Halo, tau-YFP, and GAPDH in HEK293T tau biosensor cells transfected with Halo, PS_{42}-Halo, FAF2-Halo and $FAF2_{PS}$-Halo constructs. (**G**) Quantification of tau levels as a percent relative to Halo-transfected controls, measured by Western blot and normalized to GAPDH as in (B). Data represent mean, SEM and individual values of each replicate. Statistics performed with one-way ANOVA and Tukey's multiple comparisons test. n=3. (**H**) Representative distribution of tau-YFP signal in the top 10% of Halo expressing cells that are FRET- transfected with Halo, PS_{42}-Halo, FAF2-Halo and $FAF2_{PS}$-Halo relative to mode. (**I**) Representative distribution of Tau-YFP signal in the top 10% of Halo expressing cells that are FRET+ transfected with constructs as in (F).

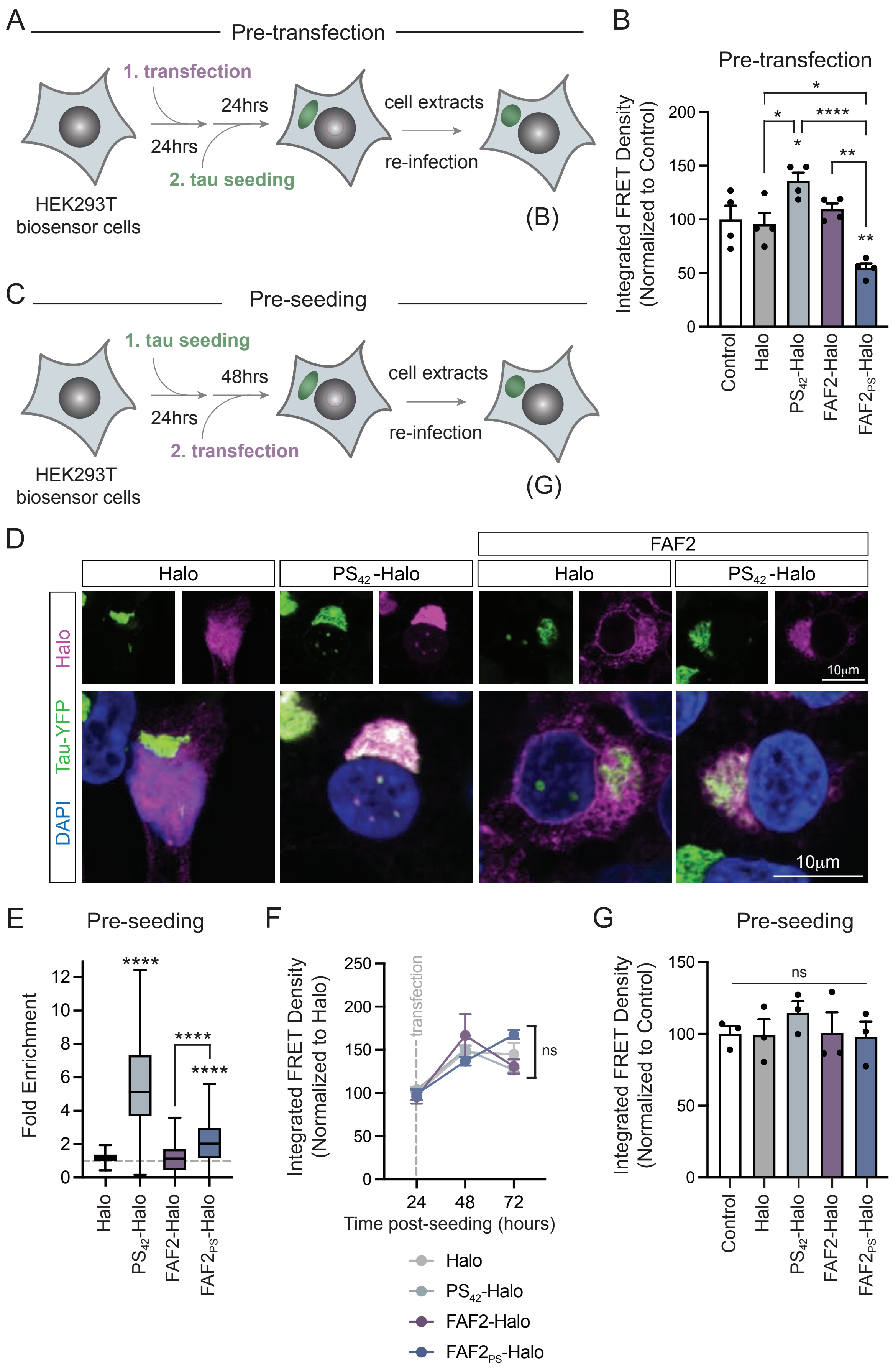

A
Pre-transfection
1. transfection
24hrs
24hrs
2. tau seeding
HEK293T
biosensor cells
cell extracts
re-infection
(B)
B
Pre-transfection
Integrated FRET Density (Normalized to Control)
Control
Halo
PS42-Halo
FAF2-Halo
FAF2PS-Halo
C
Pre-seeding
1. tau seeding
48hrs
24hrs
2. transfection
HEK293T
biosensor cells
cell extracts
re-infection
(G)
D
FAF2
Halo
PS42-Halo
Halo
PS42-Halo
Halo
Tau-YFP
DAPI
10µm
10µm
E
Pre-seeding
Fold Enrichment
Halo
PS42-Halo
FAF2-Halo
FAF2PS-Halo
F
Integrated FRET Density (Normalized to Halo)
transfection
ns
Time post-seeding (hours)
Halo
PS42-Halo
FAF2-Halo
FAF2PS-Halo
G
Pre-seeding
Integrated FRET Density (Normalized to Control)
ns
Control
Halo
PS42-Halo
FAF2-Halo
FAF2PS-Halo

Figure S4

Figure S4. Polyserine targeted FAF2 suppresses tau aggregate formation without increasing seeding capacity, related to Figure 2. (**A**) Schematic of experimental timeline for (B). (**B**) Integrated FRET density of HEK293T tau biosensor cells seeded with cell extracts from HEK293T tau biosensor cells pre-transfected with denoted constructs for 24 hours, seeded with clarified tau brain homogenate, and collected 24 hours post-seeding as in (A). Data normalized to a non-transfected control. Data represent mean, SEM and individual values of each replicate. Statistics performed with one-way ANOVA and Tukey's multiple comparisons test. n=4. (**C**) Schematic of experimental timeline for (D-G). (**D**) Fluorescence imaging of DAPI (blue), tau-YFP (green) and Halo (magenta) in HEK293T tau biosensor cells transfected with clarified tau brain homogenate for 24-hours, then transfected with Halo, PS_{42}-Halo and untargeted FAF2-Halo or polySer targeted $FAF2_{PS}$-Halo, then fixed for analysis 24-hours after plasmid transfection. (**E**) Quantification of the fold enrichment of Halo signal in cytoplasmic tau aggregates in HEK293T tau biosensor cells transfected with Halo, PS_{42}-Halo, FAF2-Halo and $FAF2_{PS}$-Halo which were pre-seeded with clarified tau brain homogenate. Boxplot represents median, interquartile range, and whiskers determined by Tukey method. Data represents fold enrichment determined for (Halo n=291, PS42-Halo n=315, FAF2-Halo n=414, $FAF2_{PS}$-Halo n=541) individual cells quantified from n=3 biological replicates. Statistics performed with one-way ANOVA and Tukey's multiple comparisons test. (**F**) Integrated FRET density of the top 10% of Halo expressing HEK293T tau biosensor cells seeded with clarified tau brain homogenate and after 24 hours transfected with Halo, PS_{42}-Halo, FAF2-Halo and $FAF2_{PS}$-Halo constructs at 24 and 48 hours post-seeding. Data represent mean and SEM. Statistics performed with two-way ANOVA and Dunnett's multiple comparisons test. n=3. (**G**) Integrated FRET density of HEK293T tau biosensor cells seeded with cell extracts from HEK293T tau biosensor cells seeded with clarified tau brain homogenate for 24 hours, then transfected with denoted construct and collected 48 hours post-seeding as detailed in (C). Data represent mean, SEM and individual values of each replicate. Statistics performed with one-way ANOVA. n=3.

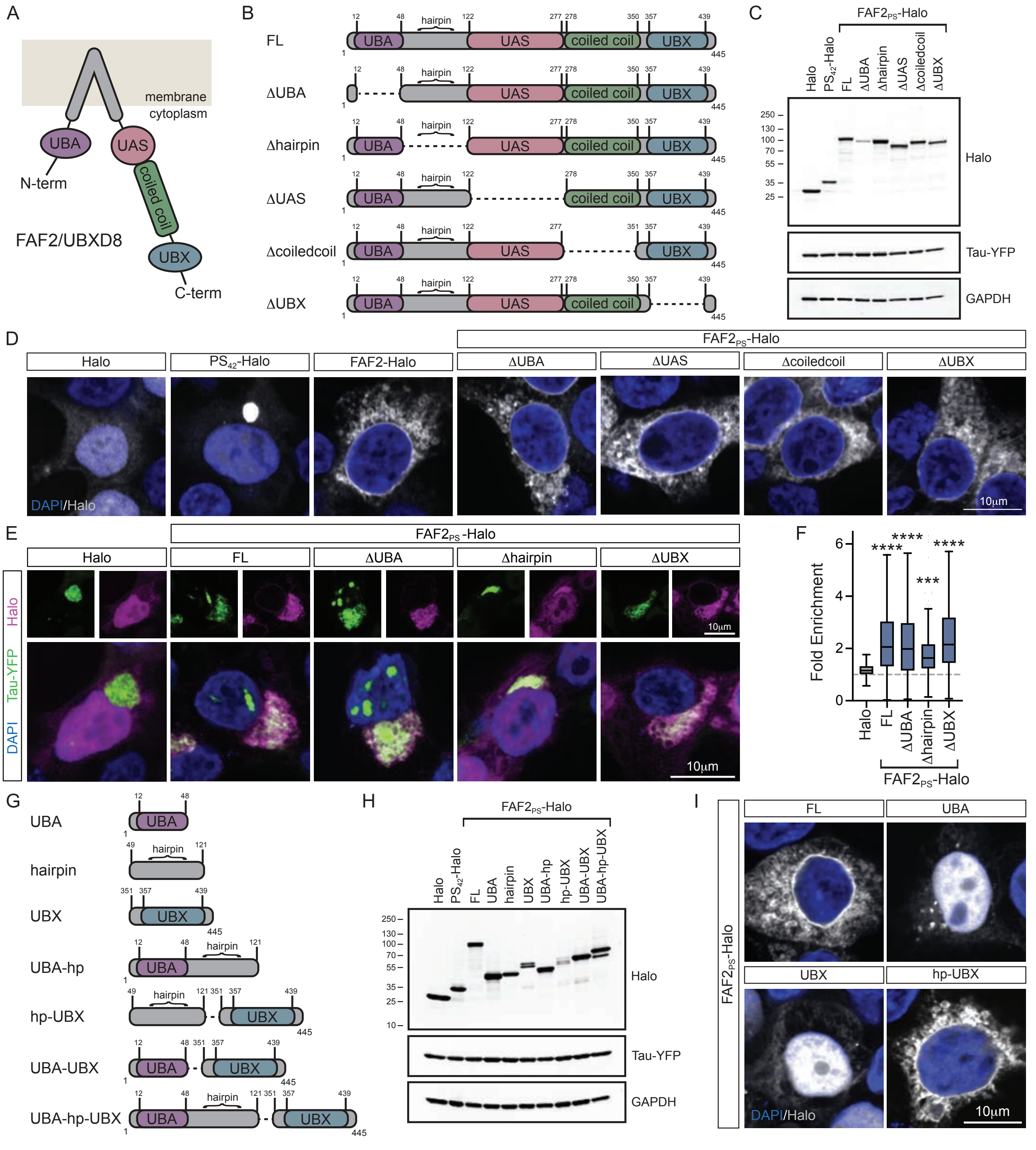
A
membrane
cytoplasm
UBA
UAS
coiled coil
N-term
FAF2/UBXD8
UBX
C-term
B
FL
ΔUBA
Δhairpin
ΔUAS
Δcoiledcoil
ΔUBX
hairpin
UBA
UAS
coiled coil
UBX
C
FAF2PS-Halo
Halo
PS42-Halo
FL
ΔUBA
Δhairpin
ΔUAS
Δcoiledcoil
ΔUBX
Halo
Tau-YFP
GAPDH
D
FAF2PS-Halo
Halo
PS42-Halo
FAF2-Halo
ΔUBA
ΔUAS
Δcoiledcoil
ΔUBX
DAPI/Halo
10μm
E
FAF2PS-Halo
Halo
FL
ΔUBA
Δhairpin
ΔUBX
Halo
Tau-YFP
DAPI
10μm
F
Fold Enrichment
FAF2PS-Halo
G
UBA
hairpin
UBX
UBA-hp
hp-UBX
UBA-UBX
UBA-hp-UBX
H
FAF2PS-Halo
Halo
PS42-Halo
FL
UBA
hairpin
UBX
UBA-hp
hp-UBX
UBA-UBX
UBA-hp-UBX
Halo
Tau-YFP
GAPDH
I
FAF2PS-Halo
FL
UBA
UBX
hp-UBX
DAPI/Halo
10μm

Figure S5

Figure S5. Characterization of FAF2 deletion mutants and minimal constructs, related to Figure 2. (**A**) Schematic of FAF2 domains and insertion at membranes. (**B**) Schematic detailing FAF2 deletion mutants. (**C**) Western blot of Halo, tau-YFP and GAPDH in HEK293T biosensor cells transfected with Halo, PS_{42}-Halo or FAF2 FL or deletion constructs. (**D**) Fluorescence imaging of DAPI (blue) and Halo (grey) in HEK293T tau biosensor cells transfected with controls or FAF2 deletion mutants (see accompanying Figure 2F). (**E**) Fluorescence imaging of DAPI (blue), Tau-YFP (green) and Halo (magenta) in HEK293T tau biosensor cells transfected with Halo or polyserine-targeted FAF2 full length (FL) or deletion mutants (ΔUBA, Δhairpin and ΔUBX) and seeded with clarified tau brain homogenate. (**F**) Quantification of the fold enrichment of Halo signal in cytoplasmic tau aggregates in HEK293T tau biosensor cells transfected with Halo or polyserine-targeted FAF2 full length (FL) or deletion mutants (ΔUBA, Δhairpin and ΔUBX) seeded with clarified tau brain homogenate. Boxplot represents median, interquartile range, and whiskers determined by Tukey method. Data represents fold enrichment determined for (Halo n=382, $FAF2_{PS}$-Halo (FL) n=457, $FAF2_{PS}$-Halo(ΔUBA) n=946, $FAF2_{PS}$-Halo(Δhairpin) n=421, $FAF2_{PS}$-Halo(ΔUBX) n=440 individual cells quantified from n=3 biological replicates. Statistics performed with one-way ANOVA and Dunnett's multiple comparisons test. (**G**) Schematic detailing FAF2 fragments expressing individual UBA, hairpin, or UBX domains, or combinations of these three domains. (**H**) Western blot of Halo, tau-YFP and GAPDH in HEK293T biosensor cells transfected with Halo, PS_{42}-Halo or polyserine-targeted FAF2 full-length (FL) or fragments as described in (G). (**I**) Fluorescence imaging of DAPI (blue) and Halo (grey) in HEK293T tau biosensor cells transfected with controls or polyserine-targeted FAF2 full-length (FL) or fragments as detailed in (G) (see accompanying Figure 2H).

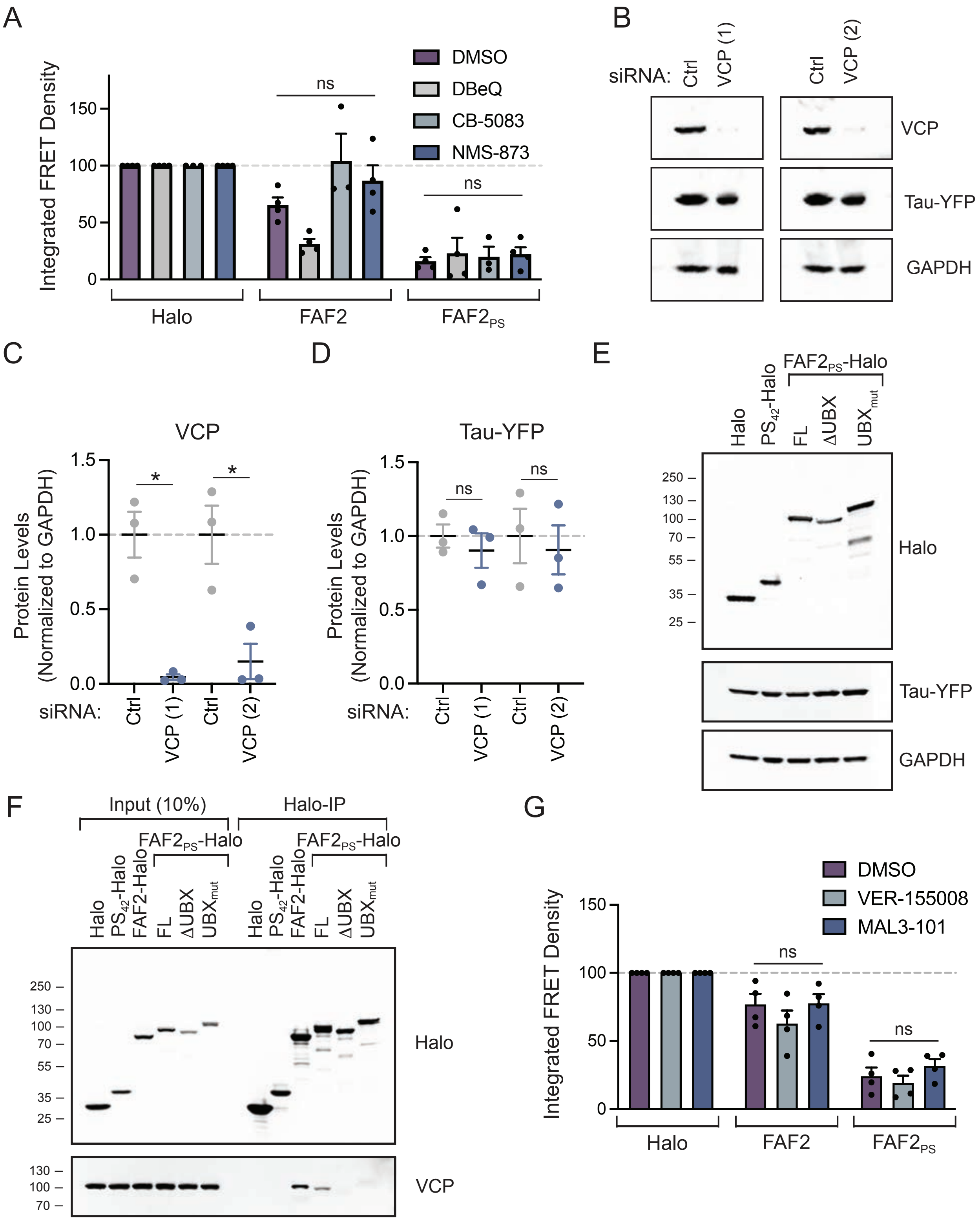

A
Integrated FRET Density
DMSO
DBeQ
CB-5083
NMS-873
ns
Halo
FAF2
FAF2PS
B
siRNA:
Ctrl
VCP (1)
VCP (2)
VCP
Tau-YFP
GAPDH
C
VCP
Protein Levels (Normalized to GAPDH)
D
Tau-YFP
E
Halo
PS42-Halo
FAF2PS-Halo
FL
ΔUBX
UBXmut
F
Input (10%)
Halo-IP
FAF2-Halo
G
VER-155008
MAL3-101

Figure S6

Figure S6. Polyserine targeted FAF2 suppresses tau aggregation independent of VCP, related to Figure 3. (**A**) Integrated FRET density of the top 10% of Halo expressing HEK293T tau biosensor cells pre-transfected with untargeted Halo-tagged FAF2 or polySer targeted FAF2, seeded with clarified tau brain homogenate, treated with DMSO or VCP inhibitors (DBeQ [5 μM], CB-5083 [2.5 μM], NMS-873 [2 μM]) and normalized to Halo control. Data represent mean, SEM and individual values of each replicate. Statistics performed with one-way ANOVA. n=4. (**B**) Representative Western blot of VCP, tau-YFP and GAPDH in HEK293T biosensor cells transfected with control or two unique VCP siRNAs 48 hours post-transfection. (**C**) Quantification of VCP protein levels following transfection of control, VCP (1) or VCP (2) siRNAs as in (A). Data represent mean, SEM and individual values of each replicate. Statistics performed with unpaired t-test. n=3. (**D**) Quantification of tau protein levels following transfection of control, VCP (1) or VCP (2) siRNAs as in (B). Data represent mean, SEM and individual values of each replicate. Statistics performed with unpaired t-test. n=3. (**E**) Western blot of Halo, tau-YFP and GAPDH in HEK293T biosensor cells transfected with Halo, PS_{42}-Halo and FL, UBX deletion mutant (ΔUBX), or UBX VCP-binding deficient mutant (UBX_{mut}) $FAF2_{PS}$-Halo. (**F**) Western blot of Halo and VCP from input and Halo immunoprecipitated samples of cells expressing Halo, PS_{42}-Halo or $FAF2_{PS}$-Halo full-length (FL), UBX deletion (ΔUBX) or UBX mutation (UBX_{mut}). (**G**) Integrated FRET density of the top 10% of Halo expressing HEK293T tau biosensor cells pre-transfected with untargeted Halo-tagged FAF2 or polySer targeted FAF2, seeded with clarified tau brain homogenate, treated with DMSO or Hsp70 inhibitors (VER-155008 [10 μM], MAL3-101 [5 μM]) and normalized to Halo control. Data represent mean, SEM and individual values of each replicate. Statistics performed with one-way ANOVA. n=4.

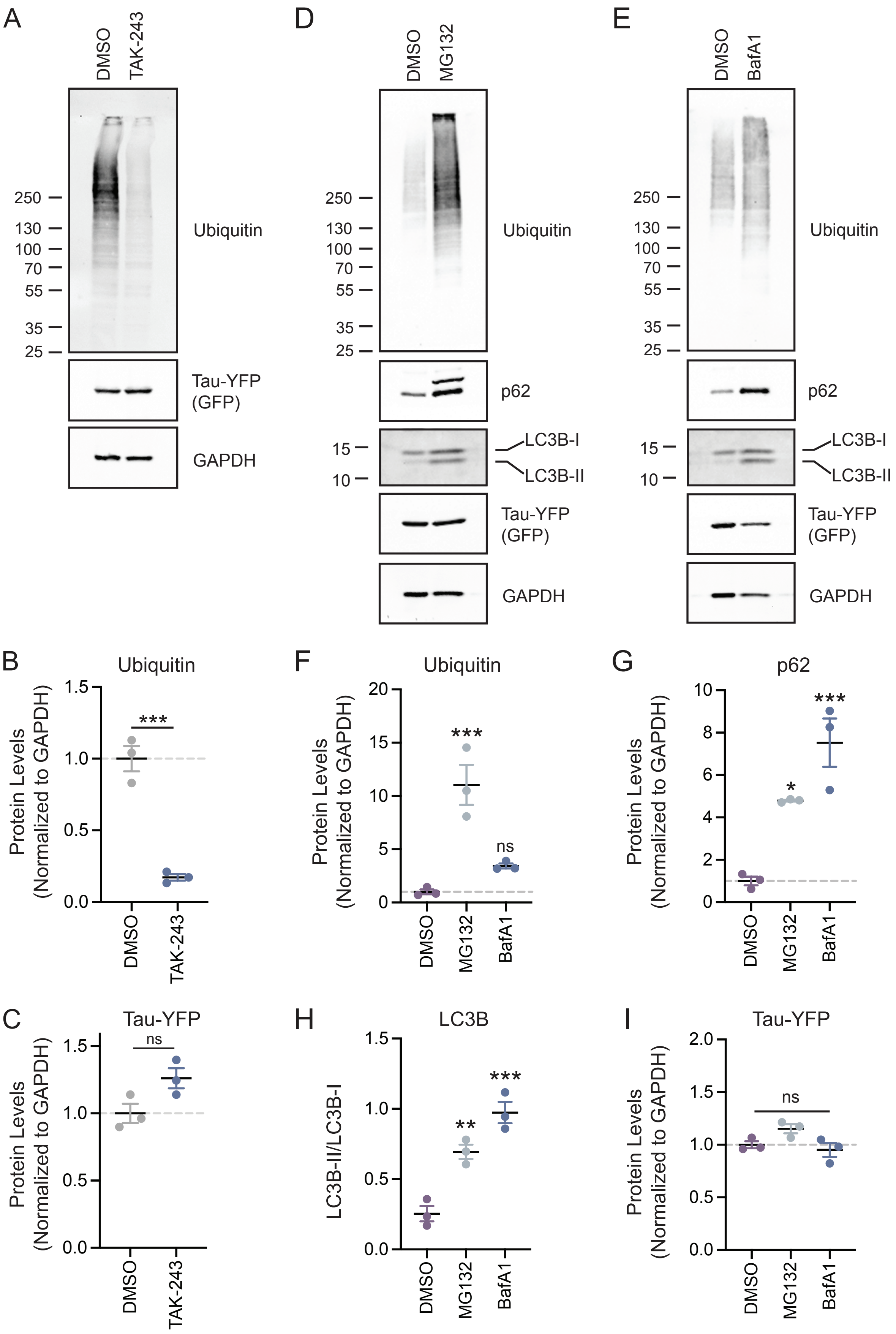
A
DMSO
TAK-243
250
130
100
70
55
35
25
Ubiquitin
Tau-YFP (GFP)
GAPDH
D
DMSO
MG132
Ubiquitin
p62
LC3B-I
LC3B-II
15
10
E
DMSO
BafA1
B
Ubiquitin
Protein Levels (Normalized to GAPDH)
***
C
Tau-YFP
ns
F
ns
G
p62
*
H
LC3B
LC3B-II/LC3B-I
**
I
Tau-YFP

Figure S7

Figure S7. Validation of ubiquitin ligase, autophagy and proteasomal inhibition, related to Figure 3. (**A**) Representative Western blot of ubiquitin, tau-YFP and GAPDH in HEK293T biosensor cells treated with DMSO or TAK-243 [0.2 μM] for 24 hours. (**B**) Quantification of ubiquitin levels following treatment with DMSO or TAK-243 as in (A). Data represent mean, SEM and individual values of each replicate. Statistics performed with unpaired t-test. n=3. (**C**) Quantification of tau-YFP levels following treatment with DMSO or TAK-243 as in (A). Data represent mean, SEM and individual values of each replicate. Statistics performed with unpaired t-test. n=3. (**D**) Representative Western blot of ubiquitin, p62 LC3B, Tau-YFP and GAPDH in HEK293T cells treated with DMSO or MG132 [5 μM]. (**E**) Representative Western blot of ubiquitin, p62 LC3B, tau-YFP and GAPDH in HEK293T tau biosensor cells treated with DMSO or BafA1 [50 nM]. (**F**) Quantification of ubiquitin protein levels in HEK293T tau biosensor cells treated with DMSO, MG132 [5 μM] and BafA1 [50 nM] normalized to GAPDH. Data represent mean, SEM and individual values of each replicate. Statistics performed with one way ANOVA and Dunnett multiple comparisons test. n=3. (**G**) Quantification of p62 protein levels in HEK293T tau biosensor cells treated with DMSO, MG132 [5 μM] and BafA1 [50 nM] normalized to GAPDH. Data represent mean, SEM and individual values of each replicate. Statistics performed with one way ANOVA and Dunnett multiple comparisons test. n=3. (**H**) Quantification of the ratio of LC3B-I and -II protein levels in HEK293T tau biosensor cells treated with DMSO, MG132 [5 μM] and BafA1 [50 nM]. Data represent mean, SEM and individual values of each replicate. Statistics performed with one way ANOVA and Dunnett multiple comparisons test. n=3. (**I**) Quantification of tau-YFP protein levels in HEK293T tau biosensor cells treated with DMSO, MG132 [5 μM] and BafA1 [50 nM] normalized to GAPDH. Data represent mean, SEM and individual values of each replicate. Statistics performed with one way ANOVA and Dunnett multiple comparisons test. n=3.

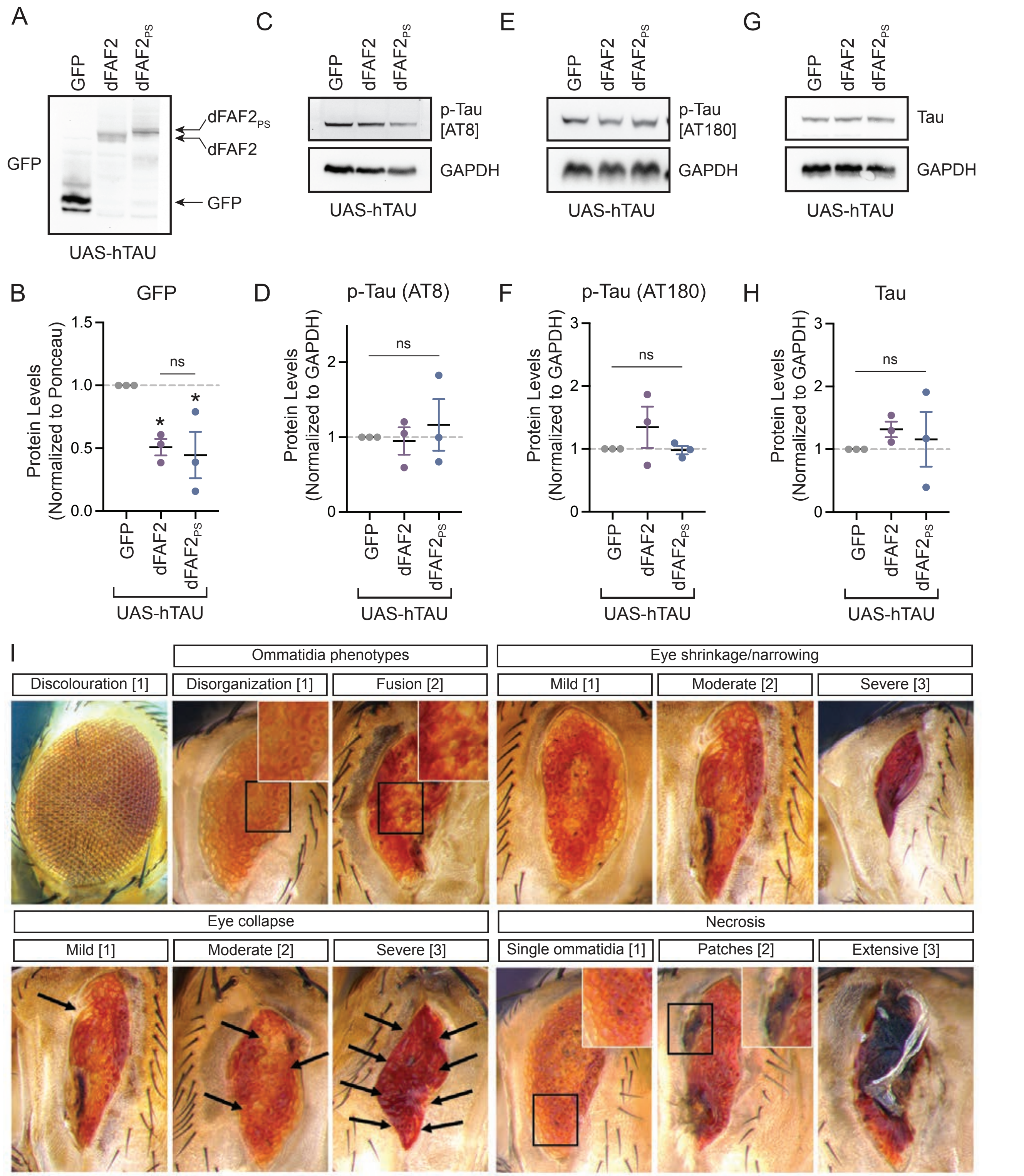
A
GFP
dFAF2
dFAF2PS
GFP
dFAF2PS
dFAF2
GFP
UAS-hTAU
C
p-Tau [AT8]
GAPDH
UAS-hTAU
E
p-Tau [AT180]
GAPDH
UAS-hTAU
G
Tau
GAPDH
UAS-hTAU
B
GFP
Protein Levels (Normalized to Ponceau)
ns
UAS-hTAU
D
p-Tau (AT8)
Protein Levels (Normalized to GAPDH)
ns
UAS-hTAU
F
p-Tau (AT180)
Protein Levels (Normalized to GAPDH)
ns
UAS-hTAU
H
Tau
Protein Levels (Normalized to GAPDH)
ns
UAS-hTAU
I
Ommatidia phenotypes
Eye shrinkage/narrowing
Discolouration [1]
Disorganization [1]
Fusion [2]
Mild [1]
Moderate [2]
Severe [3]
Eye collapse
Necrosis
Mild [1]
Moderate [2]
Severe [3]
Single ommatidia [1]
Patches [2]
Extensive [3]

Figure S8

Figure S8. Validation of transgene expression and scoring system for Drosophila tau-induced degeneration, related to Figure 5. (**A**) Representative Western blot of GFP in fly head lysates (10 heads per lane) from one-day old flies ectopically expressing human 2N4R tau and either GFP, FAF2-GFP or $FAF2_{PS}$-GFP in the eye via the Gal4-UAS system and GMR-Gal4. (**B**) Quantification of GFP levels normalized to PonceauS staining of total protein levels from groups as in (A). Data represent mean, SEM and individual values of each replicate. Statistics performed with one-way ANOVA with Tukey's multiple comparisons test. n=3. (**C**) Representative Western blot of phosphorylated (AT8) tau (p-tau) and GAPDH as a loading control in fly head lysates as in (A). (**D**) Quantification of p-tau levels normalized to GAPDH from groups as in (C). Data represent mean, SEM and individual values of each replicate. Statistics performed with one-way ANOVA with Tukey's multiple comparisons test. n=3. (**E**) Representative Western blot of phosphorylated (AT180) tau (p-tau) and GAPDH as a loading control in fly head lysates as in (A). (**F**) Quantification of p-tau levels normalized to GAPDH from groups as in (E). Data represent mean, SEM and individual values of each replicate. Statistics performed with one-way ANOVA with Tukey's multiple comparisons test. n=3. (**G**) Representative Western blot of total tau and GAPDH as a loading control in fly head lysates as in (A). (**H**) Quantification of tau levels normalized to GAPDH from groups as in (G). Data represent mean, SEM and individual values of each replicate. Statistics performed with one-way ANOVA with Tukey's multiple comparisons test. n=3. (**I**) Representative images of the set of pre-defined anatomical features which were used to assess degeneration. Points assigned to each feature are shown in brackets. Phenotype scores for each eye imaged were determined by the summation of values for each phenotype displayed. Individual eyes could be scored between 0 and 12 with higher scores being indicative of a more severe degenerative phenotype.

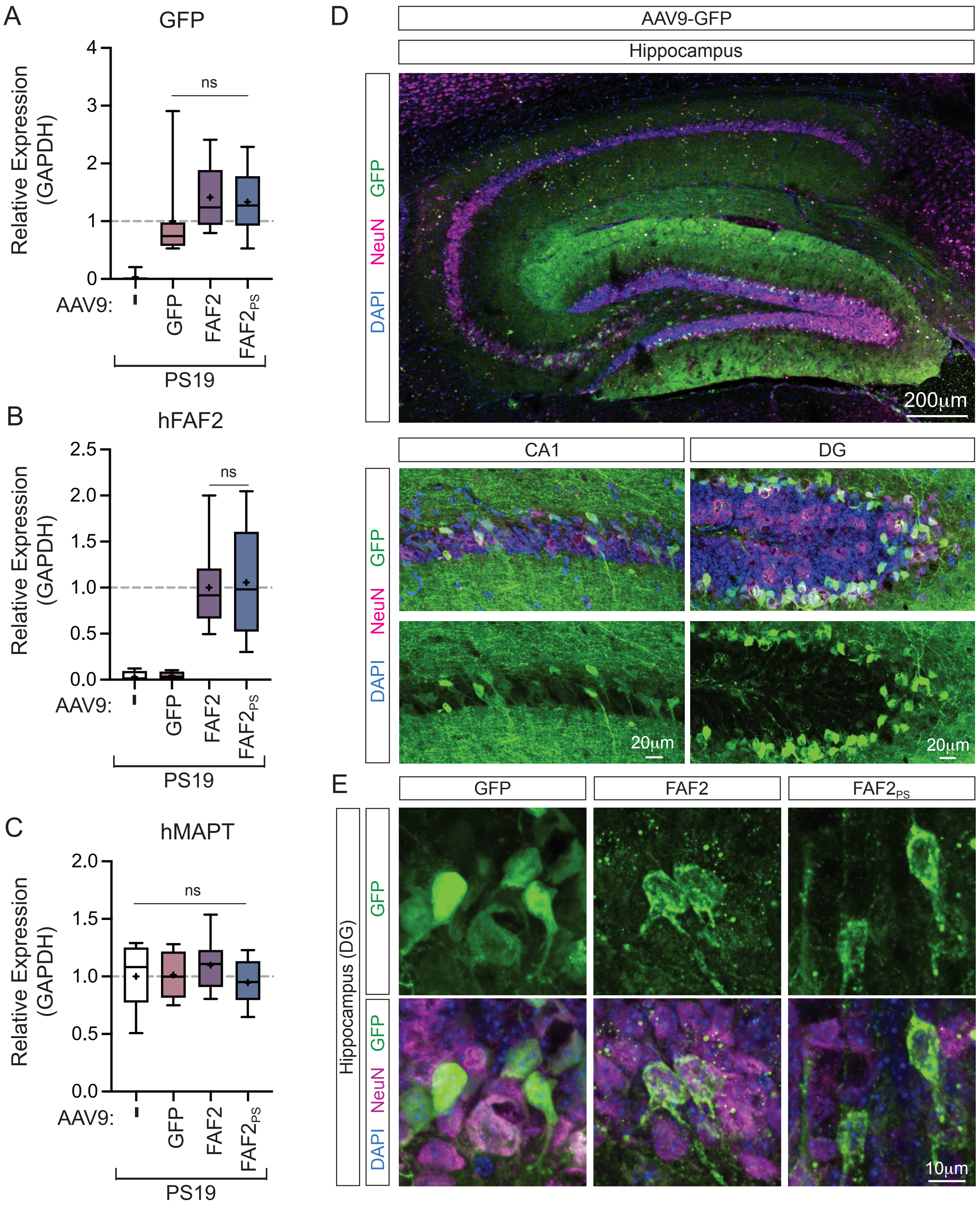
A
GFP
Relative Expression (GAPDH)
ns
AAV9:
GFP
FAF2
FAF2PS
PS19
B
hFAF2
C
hMAPT
D
AAV9-GFP
Hippocampus
DAPI NeuN GFP
200µm
CA1
DG
20µm
E
FAF2
Hippocampus (DG)
10µm

Figure S9

Figure S9. AAV9-mediated delivery of targeted FAF2 and controls in mice, related to Figures 5-7. (**A**) RT-qPCR for GFP mRNA expression in the spinal cord of 6-month-old PS19 animals uninjected (-) or injected with denoted AAV9. Boxplot represents median, mean (+), interquartile range, minimum and maximum. Statistics performed with one-way ANOVA and Tukey's multiple comparisons test. Uninjected n=11; GFP n=8, FAF2 and $FAF2_{PS}$ n=9. (**B**) RT-qPCR for human FAF2 mRNA expression in the spinal cord of 6-month-old PS19 animals uninjected (-) or injected with denoted AAV9. Boxplot represents median, mean (+), interquartile range, minimum and maximum. Statistics performed with one-way ANOVA and Tukey's multiple comparisons test. Uninjected n=11; GFP n=8, FAF2 and $FAF2_{PS}$ n=9. (**C**) RT-qPCR for human MAPT mRNA expression in the spinal cord of 6-month-old PS19 animals uninjected (-) or injected with denoted AAV9. Boxplot represents median, mean (+), interquartile range, minimum and maximum. Statistics performed with one-way ANOVA and Tukey's multiple comparisons test. Uninjected n=11; GFP n=8, FAF2 and $FAF2_{PS}$ n=9. (**D**) Immunostaining of DAPI (blue), NeuN (magenta) and GFP (green) in 6 month hippocampus of a WT AAV9-GFP injected animal. CA1 and DG hippocampal regions are shown larger and with GFP split channel. (**E**) Immunostaining of GFP (green), NeuN (magenta) and DAPI (blue) in the DG hippocampal region of 6-month-old PS19 mice injected with AAV9 expressing GFP, FAF2-GFP or $FAF2_{PS}$-GFP.

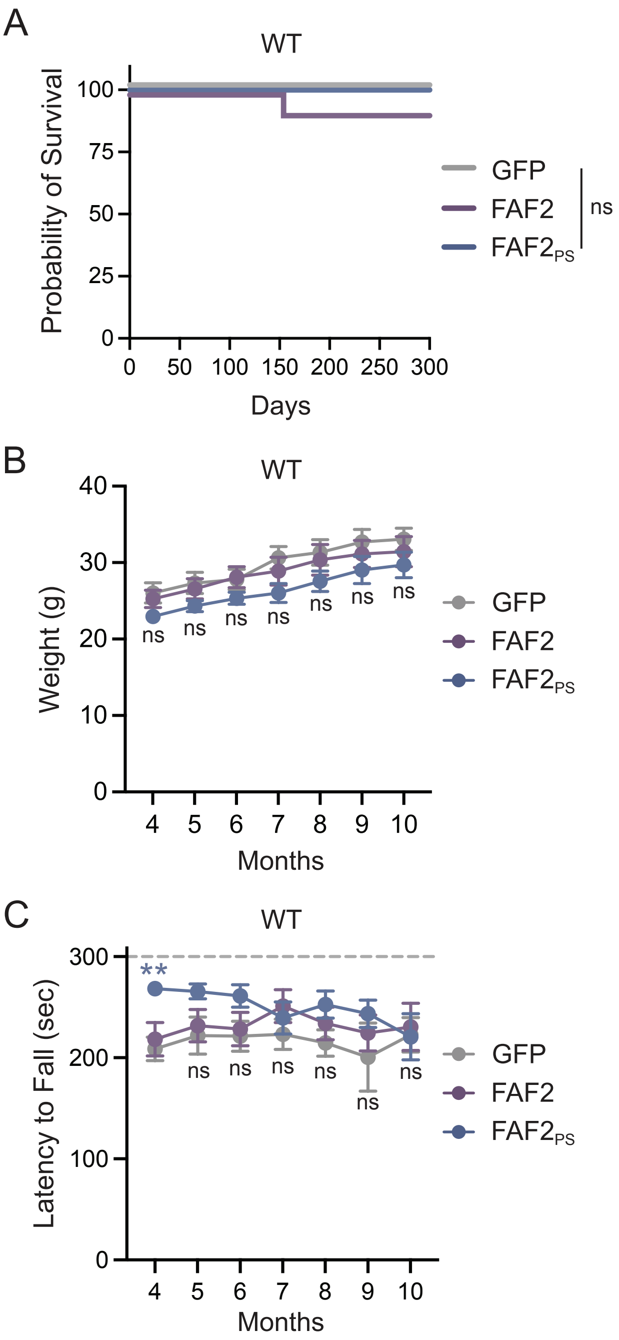
A
WT
Probability of Survival
Days
GFP
FAF2
FAF2PS
ns
B
WT
Weight (g)
Months
C
WT
Latency to Fall (sec)
Months
**
D
PS19
Probability of Survival
Days
E
PS19
Weight (g)
Months
F
PS19
Latency to Fall (sec)
Months
*

Figure S10

Figure S10. AAV9-delivery of targeted FAF2 does not alter survival or weight in WT or PS19 mice, related to Figures 5-7. (**A**) Kaplan-Meier survival curve for WT mice injected with AAV9 for GFP (n=14), FAF2-GFP (n=12) or $FAF2_{PS}$-GFP (n=12). Statistics performed with Mantel-Cox test. (**B**) Weight of WT mice injected with AAV9 for GFP, FAF2-GFP or $FAF2_{PS}$-GFP. Data represent mean and SEM. Statistics performed with two-way ANOVA and Tukey's multiple comparisons test. (4-6 months: GFP n=14, FAF2 n=12, $FAF2_{PS}$ n=12; 7-10 months GFP n=7, FAF2 n=8, $FAF2_{PS}$ n=8). (**C**) Latency to fall on rotarod for WT mice injected with AAV9 for GFP, FAF2-GFP or $FAF2_{PS}$-GFP. Data represent mean and SEM. Statistics performed with two-way ANOVA and Tukey's multiple comparisons test. (4-6 months: GFP n=14, FAF2 n=12, $FAF2_{PS}$ n=12 ; 7-10 months GFP n=7, FAF2 n=8, $FAF2_{PS}$ n=8). (**D**) Kaplan-Meier survival curve for PS19 mice injected with AAV9 for GFP (n=24), FAF2-GFP (n=24) or $FAF2_{PS}$-GFP (n=26). Statistics performed with Mantel-Cox test. (**E**) Weight of PS19 mice injected with AAV9 for GFP, FAF2-GFP or $FAF2_{PS}$-GFP. Data represent mean and SEM. Statistics performed with two-way ANOVA and Tukey's multiple comparisons test. (4-6 months: GFP n=19, FAF2 n=24, $FAF2_{PS}$ n=21 ; 7-10 months GFP n=11, FAF2 n=10, $FAF2_{PS}$ n=12). (**F**) Latency to fall on rotarod for PS19 mice injected with AAV9 for GFP, FAF2-GFP or $FAF2_{PS}$-GFP. Data represent mean and SEM. Statistics performed with two-way ANOVA and Tukey's multiple comparisons test (4-6 months: GFP n=19, FAF2 n=24, $FAF2_{PS}$ n=21 ; 7-10 months GFP n=11, FAF2 n=10, $FAF2_{PS}$ n=12).

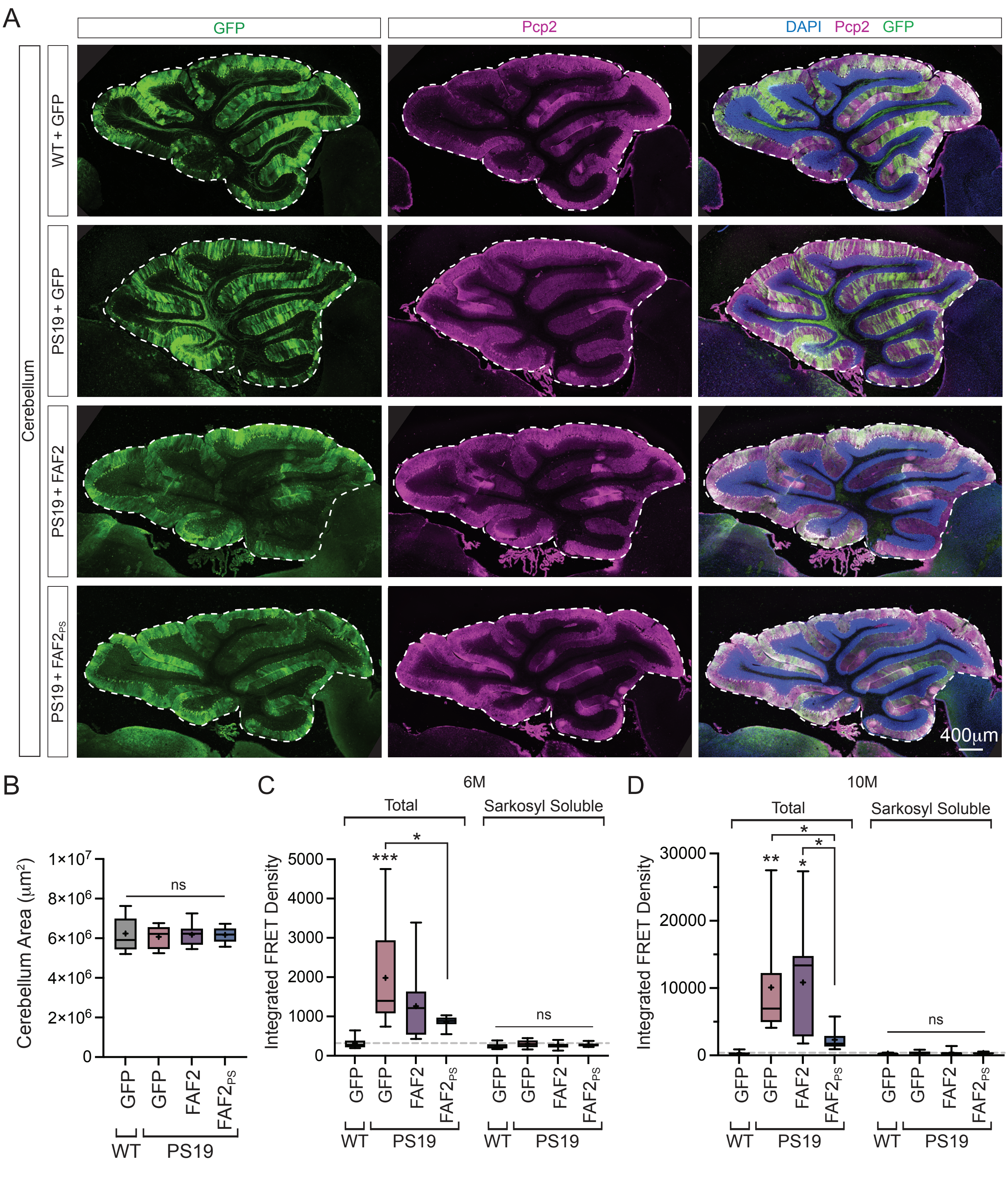

A
GFP
Pcp2
DAPI Pcp2 GFP
Cerebellum
WT + GFP
PS19 + GFP
PS19 + FAF2
PS19 + FAF2PS
400μm
B
Cerebellum Area (μm²)
ns
GFP
GFP
FAF2
FAF2PS
WT
PS19
C
6M
Total
Sarkosyl Soluble
Integrated FRET Density
***
*
ns
GFP
GFP
FAF2
FAF2PS
WT
PS19
D
10M
Total
Sarkosyl Soluble
Integrated FRET Density
**
*
*
*
ns
GFP
GFP
FAF2
FAF2PS
WT
PS19

Figure S11

Figure S11. AAV9-delivery of FAF2 does not have toxic effects in the cerebellum and reduces seeding capacity in tau transgenic mice, related to Figures 5-7. (**A**) Immunostaining of DAPI (blue), GFP (green) and Pcp2 (magenta) in the cerebellum of 6-month-old WT or PS19 mice injected with AAV9 for GFP, FAF2-GFP or $FAF2_{PS}$-GFP. (**B**) Quantification of the cross-sectional area of the cerebellum as in (A). Boxplot represents median, mean (+), interquartile range, minimum and maximum. Statistics performed with one-way ANOVA and Tukey's multiple comparisons test. n=9 sections from n=3 animals per group. (**C**) Integrated FRET density determined by flow cytometry following transfection of tau biosensor cells with total or sarkosyl soluble brain extracts from 6-month-old WT or PS19 mice either uninjected or injected with AAV9 expressing GFP, FAF2-GFP or $FAF2_{PS}$-GFP. Boxplot represents median, mean (+), interquartile range, minimum and maximum. Statistics performed for either total or sarkosyl soluble with one-way ANOVA and Tukey's multiple comparisons test. n=9 animals per group. (**D**) Integrated FRET density determined by flow cytometry following transfection of tau biosensor cells with total or sarkosyl soluble brain extracts from 10-month-old WT or PS19 mice either uninjected or injected with AAV9 expressing GFP, FAF2-GFP or $FAF2_{PS}$-GFP. Boxplot represents median, mean (+), interquartile range, minimum and maximum. Statistics performed for either total or sarkosyl soluble with one-way ANOVA and Tukey's multiple comparisons test. n=9 animals per group.

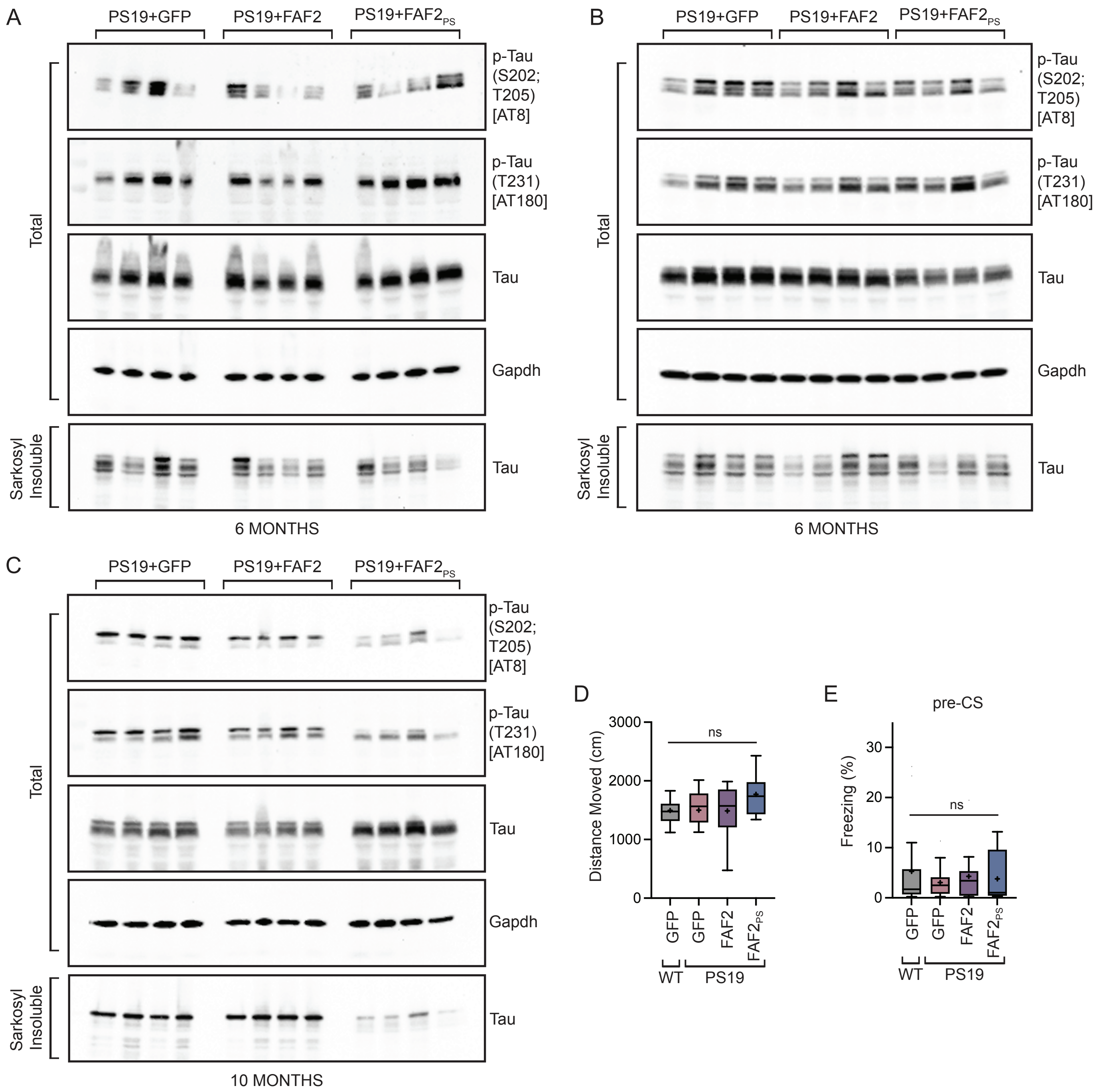
A
PS19+GFP
PS19+FAF2
PS19+FAF2PS
p-Tau (S202; T205) [AT8]
p-Tau (T231) [AT180]
Tau
Gapdh
Total
Sarkosyl Insoluble
Tau
6 MONTHS
B
PS19+GFP
PS19+FAF2
PS19+FAF2PS
p-Tau (S202; T205) [AT8]
p-Tau (T231) [AT180]
Tau
Gapdh
Total
Sarkosyl Insoluble
Tau
6 MONTHS
C
PS19+GFP
PS19+FAF2
PS19+FAF2PS
p-Tau (S202; T205) [AT8]
p-Tau (T231) [AT180]
Tau
Gapdh
Total
Sarkosyl Insoluble
Tau
10 MONTHS
D
Distance Moved (cm)
3000
2000
1000
0
ns
GFP
GFP
FAF2
FAF2PS
WT
PS19
E
pre-CS
Freezing (%)
30
20
10
0
ns
GFP
GFP
FAF2
FAF2PS
WT
PS19

Figure S12

Figure S12. Polyserine targeted FAF2 suppresses markers of tau pathology in transgenic mice, related to Figures 6-7. (**A**) Western blot of phospho-tau (AT8 and AT180), total tau and GAPDH in total brain extracts and total tau in sarkosyl insoluble extracts from AAV9-GFP. AAV9-FAF2 and AAV9-FAF2$_{PS}$ injected PS19 mice at 6-months of age. n=4 animals per group. See quantification in Figure 6F-I. (**B**) Western blot of phospho-tau (AT8 and AT180), total tau and GAPDH in total brain extracts and total tau in sarkosyl insoluble extracts from AAV9-GFP. AAV9-FAF2 and AAV9-FAF2$_{PS}$ injected PS19 mice at 6-months of age. n=4 animals per group. See quantification in Figure 6F-I. (**C**) Western blot of phospho-tau (AT8 and AT180), total tau and GAPDH in total brain extracts and total tau in sarkosyl insoluble extracts from AAV9-GFP. AAV9-FAF2 and AAV9-FAF2$_{PS}$ injected PS19 mice at 10-months of age. n=4 animals per group. See quantification in Figure 6F-I. (**D**) Distance moved during elevated plus maze assay for 10-month-old WT or PS19 animals treated with AAV-GFP, AAV-FAF2 or AAV-FAF2$_{PS}$. Boxplot represents median, mean (+), interquartile range, and whiskers determined by Tukey method. Statistics performed with one-way ANOVA. WT+GFP n=16, PS19+GFP n=18, PS19+FAF2 n=11, PS19+FAF2$_{PS}$ n=12. (**E**) Average percent of time spent freezing during the time prior to cued stimulus (pre-CS) for 10-month-old WT or PS19 animals treated with AAV-GFP, AAV-FAF2 or AAV-FAF2$_{PS}$ following conditioning in fear conditioning assay. Boxplot represents median, mean (+), interquartile range, and whiskers determined by Tukey method. Statistics performed with one-way ANOVA and Tukey's multiple comparisons test. WT+GFP n=18, PS19+GFP n=19, PS19+FAF2 n=11, PS19+FAF2$_{PS}$ n=11.
